# Synthesis, Analytical Characterization, and Human CB_1_ Receptor Binding Studies of the Chloroindole Analogues of the Synthetic Cannabinoid MDMB-CHMICA

**DOI:** 10.3390/biom14111414

**Published:** 2024-11-06

**Authors:** Sascha Münster-Müller, Steven Hansen, Tobias Lucas, Arianna Giorgetti, Lukas Mogler, Svenja Fischmann, Folker Westphal, Volker Auwärter, Michael Pütz, Till Opatz

**Affiliations:** 1 Federal Criminal Police Office, Forensic Science Institute, Äppelallee 45, 65203 Wiesbaden, Germany; smuenster@eu.sterigenics.com (S.M.-M.); michael.puetz@bka.bund.de (M.P.); 2 Joint Mass Spectrometry Centre, Institute of Chemistry, Chair of Analytical Chemistry, University of Rostock, 18057 Rostock, Germany; 3 Department of Chemistry, Johannes Gutenberg University Mainz, Duesbergweg 10–14, 55128 Mainz, Germany; steven.hansen@sgs.com (S.H.); tobiaslucas.chem@gmail.com (T.L.); 4 Unit of Legal Medicine, Department of Medical and Surgical Sciences, University of Bologna, Via Irnerio 49, 40126 Bologna, Italy; arianna.giorgetti@unibo.it; 5 Institute of Forensic Medicine, Forensic Toxicology, Medical Center, University of Freiburg, Albertstr. 9, 79104 Freiburg, Germany; lukas.mogler@lonza.com (L.M.); volker.auwaerter@uniklinik-freiburg.de (V.A.); 6 Faculty of Medicine, University of Freiburg, Breisacher Str. 153, 79110 Freiburg, Germany; 7 State Bureau of Criminal Investigation Schleswig-Holstein, Forensic Science Institute, Mühlenweg 166, 24116 Kiel, Germany; svenja.fischmann@projekt-adebar.eu (S.F.); folker.dr.westphal@polizei.landsh.de (F.W.)

**Keywords:** new psychoactive substances (NPSs), MDMB-CHMICA, cannabimimetics, analytical characterization, pharmacological evaluation

## Abstract

Synthetic cannabinoids (SCs) are one of the largest groups of new psychoactive substances (NPSs). However, the relationship between their chemical structure and the affinity to human CB_1_ receptors (hCB_1_), which mediates their psychotropic activity, is not well understood. Herein, the synthesis of the 2-, 4-, 5-, 6- and 7-chloroindole analogues of the synthetic cannabimimetic MDMB-CHMICA, along with their analytical characterization via ultraviolet–visible (UV/VIS), infrared (IR), nuclear magnetic resonance (NMR) spectroscopy, and mass spectrometry, is described. Furthermore, all five derivatives of MDMB-CHMICA were analyzed for their hCB_1_ binding affinities. Chlorination at position 4 and 5 of the indole core reduced the binding affinity compared to MDMB-CHMICA, while the test compounds chlorinated in positions 2, 6, and 7 largely retained their binding affinities relative to the non-chlorinated parent compound.

## 1. Introduction

Since their first detection in “Spice” in 2008, synthetic cannabinoids have become one of the largest sub-groups of new psychoactive substances and are marketed in many countries as undisclosed components of seemingly harmless herbal blends, sometimes advertised as “legal” substitutes for marijuana, yet fatalities have resulted from their recreational use [1]. The term NPS encompasses different groups of substances, which mainly consist of synthetic derivatives and analogues designed to mimic the effect of traditional psychoactive drugs. Similarly, synthetic cannabinoids mimic the effect of cannabis by binding to the human cannabinoid receptor CB_1_ (hCB_1_), mainly located in the central nervous system (CNS), and, to a lesser extent, to hCB_2_. Although originally developed, at least in part, as highly specific medications to modulate the immune system and for their potential benefits, their medical use has been strongly limited by their toxicity [2]. Indeed, compared to Δ^9^-tetrahydrocannabinol (THC), the main psychoactive constituent of natural cannabis, they generally display greater binding affinity to hCB_1_ and act as full agonists, resulting in severe adverse effects including nausea, vomiting, cardiovascular abnormalities, particularly tachycardia, anxiety, agitation, psychosis, respiratory depression, and even death [3,4]. The binding affinity of synthetic cannabinoids to hCB_1_ has significant pharmacological and clinical implications, since it might be of interest in terms of the development of medicinal drugs, but also because it is thought to contribute to their severe health effects and high potential for abuse [5]. Therefore, it has been studied to assess the properties of synthetic cannabinoids that have already been introduced to the NPS market and to predict undiscovered structural or substructural modifications [6,7].

Until December 2022, a total of 930 NPSs have been monitored by the European Monitoring Centre for Drugs and Drug Addiction (EMCDDA), 245 of which are represented by synthetic cannabinoids [8]. From a German perspective, this flood of substances posed a major challenge for legislation. The implementation of single substances to the Narcotics Act is a rather time-consuming process and only the most prevalent and harmful substances are considered for classification as a narcotic drug. Since November 2016, the new psychoactive substance law (NpSG) [9] was put into force in Germany, regulating the trade, marketing, manufacture, transfer (import, export, or transit), and acquisition of NPSs, in particular synthetic cannabinoids. The intention was to regulate the general structural classes of already existing NPSs instead of single compounds, but also to stay one step ahead of the producers and regulate all the closely related derivatives of NPSs that have not yet appeared on the market. Thenceforward, several synthetic cannabinoids were classified as NPSs by a generic definition targeting different core structures, side chains, linkers, and linker residues, and several amendments of the NpSG became effective, as synthetic cannabinoids bearing non-regulated structural elements kept appearing on the market. Examples include γ-carboline-1-ones like CUMYL-PEGACLONE and its derivatives, which contain a previously unreported core structure [10,11,12]. As a reaction, the generic definition of the NpSG was extended to these new substructures in July 2019. As a condition for the inclusion of certain substructures into the NpSG, it must be proven that this specific structural element has a high prevalence in the form of corresponding synthetic cannabinoids on the market, or that a new substructure could produce synthetic cannabinoids that are highly effective and thus are potential candidates for future generations of these compounds. Currently, the generic definition of indole substructures only comprises substitution with hydrogen, fluorine, chlorine, bromine, iodine, methyl, methoxy, and nitro groups for the indole positions 2, 5, 6, and 7. Substitutions in indole positions 2, 6, and 7 with methyl, fluorine, and bromine have been tested using the displacement of the radioligand [^3^H]-WIN 55212-2 and have shown to retain high receptor binding affinities. Fluoro, chloro, methyl, and bromo substitutions in the 5-position were instead detrimental to binding and functional activity [13,14]. Substitutions in the 4-position of the indole, also displacing [^3^H]-WIN 55212-2, are not yet regulated by the NpSG [13]. More recently, it was shown that C-5 halogenation of the indole group (Br and I) resulted in a decreased affinity for the human CB_1_ receptor (hCB_1_) [15], yet 5-chlorinated indazole-type SCs have been recently reported [16].

So far, the majority of synthetic cannabinoids feature a bicyclic indole or indazole core. Since a recent ban entered into force in China, a variety of new synthetic cannabinoids have emerged on the NPS market in an attempt to evade the legislation. These carry new substructural features, e.g., a new acetamide linker connected to an indole core, as in the case of CH-PIATA [15]. Presumably, as a result of the use of common building blocks, the core structures of synthetic cannabinoids notified to the EMCDDA (recently reorganized to EUDA) were more rarely substituted in comparison to other substructures [17]. However, brominated indazole core structures appeared in synthetic cannabinoids around 2021, as in 5-bromo-1-butyl-*N*-(1-carbamoyl-2,2-dimethyl-propyl)-indazole-3-carboxamide (semisystematic name: ADMB-5′Br-BINACA) and 5-bromo-*N*-(1-carbamoyl-2,2-dimethyl-propyl)-1*H*-indazole-3-carboxamide (semisystematic name: ADMB-5′Br-INACA). This demonstrated that new, unregulated derivatives could appear on the NPS market by a similar substitution of rather old core substructures. One possibility to circumvent the NPS legislation would be the substitution of hydrogen with chlorine in the 2- and 4-position of the indole core. However, the impact of similar substitutions on synthetic cannabinoids affinity, and thus their pharmacological properties and potential for abuse, have not been elucidated yet.

Interestingly, reduced activity at the hCB_1_ was shown by removing the bromine substitution from the core of ADMB-5′Br-INACA [18], but data on the binding affinity of synthetic cannabinoids after the substitution of hydrogen with halogens at the core are still limited. MDMB-CHMICA is a potent synthetic cannabinoid featuring an indole core that appeared in Europe around 2014 and has since become one of the most prevalent synthetic cannabinoids [19]. The compound was linked to a high number of intoxications and fatalities [20] and was detected with low concentrations in biological fluids (in the lower nanomolar range). Accordingly, MDMB-CHMICA has shown high potency at the hCB_1_ receptor [21] and is one of the synthetic cannabinoids with the highest binding affinity [22]. Given the prevalence in intoxication cases and its high affinity, MDMB-CHMICA could serve as a basis to explore the impact of minor structural modifications, such as the chloro-substitution at the indole core, on the pharmacological properties.

The present study aimed to assess the binding affinities of five chloroindole analogues of MDMB-CHMICA (**1**) in order to evaluate whether similar derivatives of synthetic cannabinoids with an indole core can be potential candidates for new compounds to be introduced to the market.

**Figure 1:**
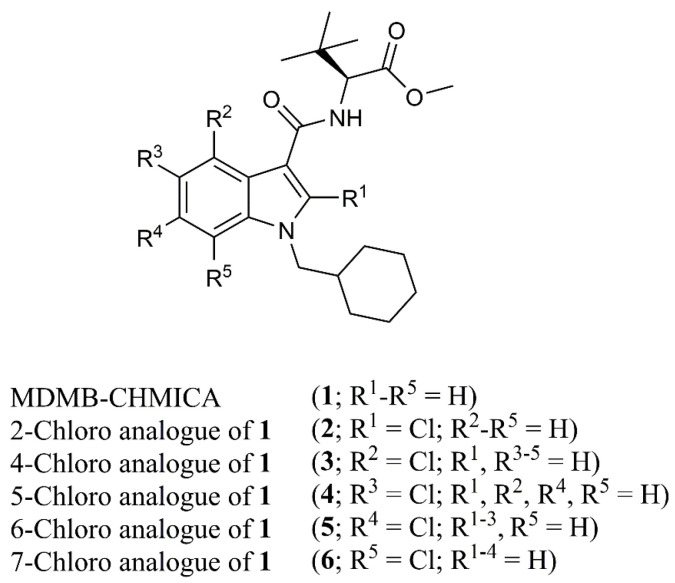
Structural formula for MDMB-CHMICA and its five chloroindole derivatives.

To this end, five chloroindole analogues of **1**, 2-Cl- (**2**), 4-Cl- (**3**), 5-Cl- (**4**), 6-Cl- (**5**), and 7-Cl-MDMB-CHMICA (**6**) (Figure 1) were synthesized, characterized by UV/VIS, IR, NMR spectroscopy and mass spectrometry. Particular attention was given to the discriminatory power of each technique, similar to studies from the group of Clark et al. [23,24]. Additionally, the hCB_1_ receptor affinity of the five compounds was tested in order to obtain relevant information for forensic toxicology, which is needed to understand which compounds are more likely to become widespread on the market and in pharmaceutical disciplines.

## 2. Materials and Methods

UHPLC measurements were conducted on a ternary system UltiMate 3000 by Dionex (Thermo Scientific, Waltham, MA, USA), consisting of a pump module, an autosampler, and a column compartment. Separation was achieved on a Kinetex C18 (2.6 μm, 100 Å, 100 × 2 mm) column from Phenomenex (Aschaffenburg, Germany) at 40 °C. Each analysis was carried out with a binary mobile phase consisting of eluent A (98.9% water, 1% acetonitrile, 0.1% formic acid, 2 mM ammonium formate) and eluent B (1% water, 98.9% acetonitrile, 0.1% formic acid, 2 mM ammonium formate). The elution program was as follows: 80% A/20% B (1 min hold), followed by 40% A/60% B (1–2.5 min) to 35% A/65% B (2.5–4 min, 1.5 min hold) and up to 1% A/99% B (5.5–8 min, 2 min hold). In the end, an equilibration step was introduced with 80% A/20% B (10–10.2 min, 1.8 min hold), giving an overall runtime of 12 min at a flow rate of 0.5 mL/min. The injection volume was set to 5 µL. For mass spectrometry, a Bruker amaZon Speed ion trap MS (Billerica, MA, USA) was operated in ultra-scan mode from 70 to 600 *m*/*z* with 32,500 *m*/*z*/s. The dry gas flow rate was set to 10 L/min at a temperature of 320 °C. UV/VIS spectra were recorded via a Shimadzu Prominence UFLC system with SPD-M20A detector.

HR-ESI-MS was performed on a Q-ToF-Ultima 3 instrument (Waters, Milford, MA, USA) with a LockSpray^TM^ interface and a suitable external calibrant.

NMR spectra were recorded on a Bruker Avance-II 400 (400 MHz for ^1^H NMR and 100.6 MHz for ^13^C NMR, including 2D NMR) using a 5 mm probe and standard pulse sequences. Chemical shifts are reported as parts per million (ppm) downfield from tetramethyl silane and are referenced to the respective residual solvent signal: CDCl_3_ (^1^H: δ = 7.26 ppm; ^13^C: δ = 77.16 ppm); DMSO-*d*_6_ (^1^H: δ = 2.50 ppm; ^13^C: δ = 39.52 ppm); Acetone-*d*_6_ (^1^H: δ = 2.05 ppm; ^13^C: δ = 206.68 ppm, 29.92 ppm).

GC-MS was recorded using a Trace GC Ultra coupled to an ITQ 1100 ion trap with a TriPlus autosampler (Thermo Scientific, Waltham, MA, USA). Injection volume was set to 1 µL in split mode (split flow 50 mL min^−1^) with an inlet temperature of 200 °C. Separation was achieved on a TG-5MS column (30 m × 0.25 mm i.d., 0.25 µm film thickness) with He as carrier gas and the following temperature gradient program: 60 °C for 1 min, ramped to 320 °C with a rate of 10 °C min^−1^, followed by a hold of 13 min. Ionization of eluting substances was achieved via electron impact ionization (EI) at 225 °C with 70 eV. The MS was operated in full scan mode from *m*/*z* 29–600 with scan times of 0.57 s.

Additionally, GC-MS was recorded using a Trace GC 1310 coupled to a Finnigan TSQ 8000 Evo triple-stage quadrupole mass spectrometer with a TriPlus autosampler (Thermo Scientific, Waltham, MA, USA). Injection volume was set to 1 µL in split mode with an inlet temperature of 280 °C. Separation was achieved on a DB-1 column (30 m × 0.25 mm i.d., 0.25 µm film thickness) with He as carrier gas and the following temperature gradient program: 80 °C for 2 min, ramped to 280 °C with a rate of 15 °C min^−1^, followed by a hold of 20 min. Ionization of eluting substances was achieved via electron impact ionization (EI) at 175 °C with 70 eV. The MS was operated in full scan mode from *m*/*z* 29–600 with scan times of 1 s.

As GC-solid phase-IR-system, an Agilent GC 7890B (Waldbronn, Germany) with probe sampler Agilent G4567A and a DiscovIR-GC™ (Spectra Analysis, Marlborough, MA, USA) was used. The column eluent was cryogenically accumulated on a spirally rotating ZnSe disc cooled by liquid nitrogen. IR spectra were recorded through the IR-transparent ZnSe disc using a nitrogen-cooled MCT detector. GC parameters: injection in splitless mode with an injection port temperature set at 240 °C and a DB-1 fused silica capillary column (30 m × 0.32 mm i.d., 0.25 µm film thickness). The carrier gas was helium with a flow rate of 2.5 mL/min and the oven temperature program was as follows: 80 °C for 2 min, ramped to 290 °C at 20 °C/min, and held at for 20 min. The transfer line was heated at 280 °C. Infrared spectroscopy conditions: oven temperature, restrictor temperature, disc temperature, and Dewar cap temperatures were 280 °C, 280 °C, −40 °C, and 35 °C, respectively. The vacuum was 0.2 mTorr, disc speed 3 mm/s, spiral separation was 1 mm, wavelength resolution 4 cm^−1^, and IR range 650–4000 cm^−1^. Acquisition time was 0.6 s/file with 64 scans/spectrum. Data were processed using GRAMS/AI Ver. 9.1 (Grams Spectroscopy Software Suite, Thermo Fisher Scientific, Dreieich, Germany) followed by implementation of the OMNIC Software, Ver. 7.4.127 (Thermo Electron Corporation, Dreieich, Germany).

ATR-IR spectra were recorded on a Tensor 27-FT-IR spectrometer (Bruker) equipped with a diamond ATR unit.

Melting point (MP) ranges were determined on a Krüss Optronic electrothermal apparatus.

Preparative normal-phase separations were performed on silica (Acros Organics, 35–70 µm) using manual flash chromatography. The eluents were purchased in technical quality and were purified by distillation prior to use.

### 2.1. General Chemical Synthesis Details

Chemicals were purchased from commercial suppliers (Sigma-Aldrich, Fisher Scientific, TCI) and were used without further purification. All air- and moisture-sensitive reactions were carried out in oven-dried glassware under an inert atmosphere of nitrogen using standard Schlenk techniques. Removal of solvents was performed using a rotary evaporator (40 °C bath temperature) and a membrane pump. The used solvents were dried and purified by standard procedures. For this purpose, dichloromethane (DCM) was distilled from CaH_2_. Dimethylformamide (DMF) was purchased in pre-dried quality from Acros Organics and stored over molecular sieves (4 Å). All other chemicals were purchased from commercial suppliers and used without further purification unless mentioned otherwise. For analytical TLC, silica-coated aluminum plates (E. Merck, silica 60 F_254_) and aluminum oxide plates (Macherey-Nagel, N/UV_245_, ALOX) were used.

### 2.2. Binding Affinities to hCB_1_ Receptors and Functional Properties

To determine receptor binding affinity to the hCB_1_ receptor for the test compounds, the previously described competitive ligand binding assay was performed using the radiolabeled hCB_1_ receptor agonist [^3^H]-CP-55,940 (Perkin Elmer, Waltham, MA, USA) [25]. Tris(hydroxymethyl)aminomethane hydrochloride (TRIS HCl), bovine serum albumin (BSA), and dimethyl sulfoxide (DMSO) were bought from Sigma Aldrich (Steinheim, Germany). Deionized water was prepared using a Medica^®^ Pro deionizer from ELGA (Celle, Germany). As a source for hCB_1_ receptors, membrane preparations of HEK293-EBNA (Perkin Elmer, Waltham, MA, USA) cells were used. The purities of the test compounds were greater than 98% (NMR), with the sole exception of compound **5**, the purity of which was 93% (NMR). Stock solutions of the test compounds were prepared in DMSO (10^−4^ M) and further diluted with deionized water to the final concentration (between 10^−12^ and 10^−5^ M). The in vitro assay was performed as described previously [25], in a final volume of 200 µL containing 2 nM concentration of radioligand, 8 µg of hCB_1_ membrane protein/well (0.04 pmol hCB_1_ receptor), 50 mM TRIS HCl, 3 mM MgCl_2_, 0.1% BSA in deionized water (pH 7.4 adjusted with 10 M NaOH), and respective concentration of the compound dilution. To determine nonspecific binding (NB, baseline), a concentration of 10^−5^ M cold CP-55,940 (Sigma-Aldrich, cold ligand) was measured instead of the test compound. Total binding (TB, maximal response) was determined using assay buffer instead of a cold agonist. To reach equilibrium, the mixture was incubated for 1 h at 37 °C. The binding reaction was stopped by rapid filtration and washing the filter plate, including six washing steps with 100 µL ice-cold assay buffer, using Millipore MultiScreenTM 96-well Vacuum Manifold (Merck, Darmstadt, Germany). Prior to filtration, the 96-well multiscreen filter plates (1.2/0.5 µm) were presoaked in 90 µL of 0.1% aqueous PEI for 60 min at room temperature (20 °C) and washed twice with 100 µL assay buffer. Afterwards, the filters were transferred to scintillation vials and dried at 50 °C for 30 min. Subsequently, 3 mL scintillation fluid (UltimaGoldTM, Perkin Elmer) was added and filters were gently shaken for 30 min. The bound radioactivity was determined by liquid scintillation counting (LSC-Analyzer Tri-Carb 2100TR, Perkin Elmer).

Experiments were performed in triplicates. MDMB-CHMICA, JWH-018 (naphthalen-1-yl(1-pentyl-1*H*-indol-3-yl)methanone) and CP 55,940 from Sigma Aldrich (Steinheim, Germany) were tested additionally. IC_50_ values were determined at the turning point of the sigmoidal graph (semilogarithmic scale of the horizontal axis), generated using a one-site fit log IC_50_ function of the statistical software. The K_i_ values (equilibrium dissociation constant in M) were calculated, considering the concentration and a K_D_ value of 0.05 nM ([^3^H]-CP-55,940 at hCB_1_) [26], using a one-site fit K_i_ function, which does not need to apply the Cheng–Prusoff correction [27], with the specific K_D_. The K_D_ value is a constant and supported for the specific hCB_1_ membrane preparations (HEK293-EBNA) by Perkin Elmer with 0.05 nM. Statistical analysis of significance was performed using the extra sum-of-squares F test on the logK_i_ values (setting a *p* < 0.005 for significance) and using ANOVA, followed by Bonferroni’s post hoc analysis [28]. For the latter, a *p*-value less than 0.05 was considered to be significant. Data were analyzed using GraphPad Prism (Version 7.00, GraphPad Software, La Jolla, CA, USA).

## 3. Results and Discussion

**Scheme 1:**
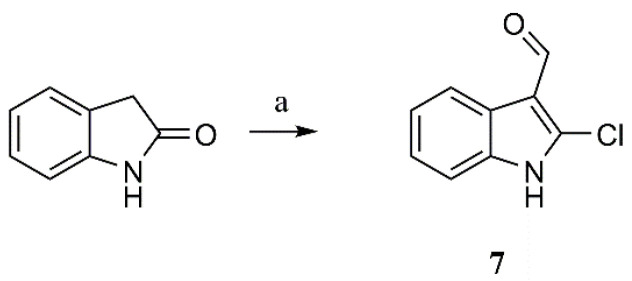
Synthesis of 2-chloroindole-3-carbaldehyde from oxindole. Reagents and conditions: (a) POCl_3_, DMF, DCM, H_2_O.

Two general procedures were used to synthesize compounds **2**–**6**. Chlorinated indole-3-carbaldehydes were N-alkylated, oxidized to the carboxylic acids, and then subjected to an amide coupling with *tert*-leucine methyl ester (TLME). Alternatively, chloroindoles were N-alkylated, trifluoroacetylated in 3-position, subjected to a haloform reaction, and the resulting carboxylic acids were coupled as described above. The precursor 2-chloroindole-3-carbaldehyde **7** was synthesized from oxindole in a separate synthesis as shown in Scheme 1.

**Scheme 2:**
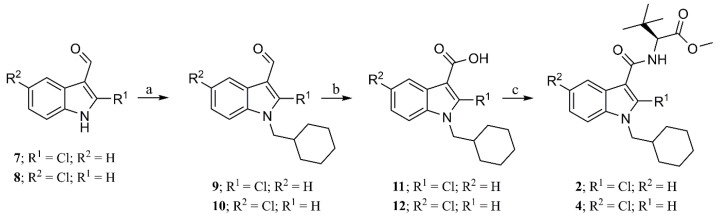
Synthesis of the 2-chloro- and 5-chloroindole analogue of MDMB-CHMICA. Reagents and conditions: (a) (i) NaH (60%), DMF; (ii) (bromomethyl)cyclohexane, DMF; (b) KMnO_4_, acetone; (c) (i) Hünig’s base, HATU, DMF; (ii) DMF, TLME. The numbers indicate the positions on the indole skeleton.

**Scheme 3:**
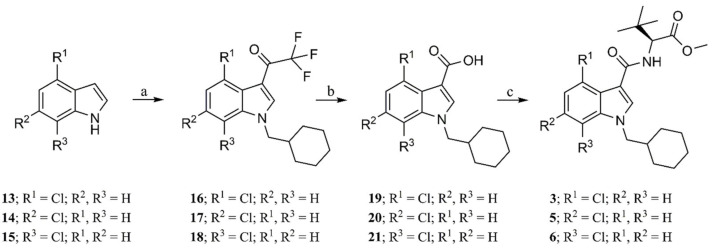
Synthesis of the 4-chloro, 6-chloro and 7-chloro indole analogue of MDMB-CHMICA. Reagents and conditions: (a) (i) NaH (60% in mineral oil), (bromomethyl)cyclohexane, DMF; (ii) (CF_3_CO)_2_O, DMF; (b) KOH, MeOH, PhMe; (c) (i) Hünig’s base, HATU, DMF; (ii) DMF, TLME. The numbers indicate the positions on the indole skeleton.

Scheme 2 shows the individual steps for the synthesis of the 2-chloro- and 5-chloroindole derivatives **2** and **4** starting from the respective 3-carbaldehydes **7** (prepared as described above) and **8** (commercial). In the first step, the respective chloroindole-3-carboxaldeyde was alkylated with (bromomethyl)cyclohexane to give **9** and **10**, followed by the oxidation of the aldehydes to the respective carboxylic acids **11** and **12**. In the last step, the carboxylic acid was activated via HATU and treated with *tert*-leucine methyl ester to afford **2** and **4**. In Scheme 3, the general procedure for **3**, **5,** and **6** is shown. In a one-pot synthesis starting from the respective chloroindoles **13**, **14,** and **15** (all commercial), N-alkylation with (bromomethyl)cyclohexane is performed, followed by conversion into 3-trifluoroacetylindoles by trifluoroacetic anhydride, yielding ketones **16**, **17,** and **18**. Subsequently, the 3-trifluoroacetylindoles were hydrolyzed to the respective carboxylic acids **19**, **20,** and **21**, which were activated with HATU and reacted with tert-leucine methyl ester to afford the amides **3**, **5,** and **6**. The analytical characterization of **1** was previously reported by Banister et al. [29]. Compound **2** was found as a synthesis impurity in the seized samples of **1**. Münster-Müller et al. [30] already reported the corresponding NMR and high-resolution MS^3^ data; however, no further characterization via common analytical tools like GC or UV/VIS was available.

### 3.1. Characterization via NMR

**Figure 2:**
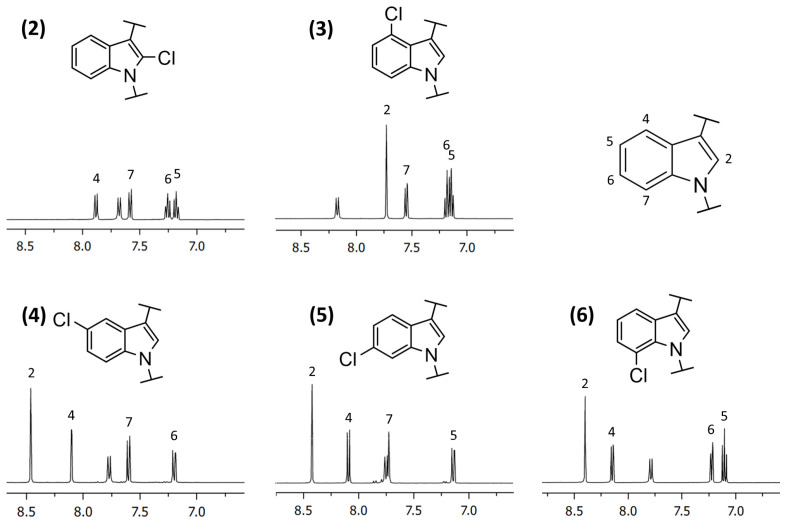
Expansion of the aromatic region of the ^1^H-NMR spectra (400 MHz, DMSO-d_6_) for the five chloroindole isomers of MDMB-CHMICA. The numbers indicate the positions on the indole skeleton of the protons causing the signals. The unlabeled doublet signals are NH resonances.

All five isomers were characterized via ^1^H and ^13^C NMR (incl. 2D) spectroscopy. The respective spectra can be found in the Supplementary Materials. Figure 2 shows excerpts of the most discriminative areas of 8.5 to 7 ppm of the ^1^H-NMR spectrum for all isomers (reported relative to tetramethylsilane) as the spectrum in the range of 7 to 0 ppm was similar for all isomers. The absence or presence of singlets in the aromatic region of the spectra are the most discriminating indicators for the substitution of hydrogen with chlorine on different positions of the indole.

Only in the spectrum of compound **2**, the singlet of 2-H is absent and the remaining signals appeared as two doublets and two apparent triplets, indicating the vicinal coupling of protons in close proximity, which is only possible for those protons on the indole positions 4 to 7. Compounds **4** and **5** can be distinguished from **3** and **6** by the absence of aromatic resonances with two large coupling constants. To unambiguously distinguish between **4** and **5** or between **3** and **6**, HMBC spectra were acquired to analyze long-range couplings. A possible indication to distinguish between compounds **3** and **6** in the ^1^H-NMR spectra could be the shielding of the 2-proton (shifted upfield) visible for **3**, when the chlorine is attached to the 4-position of the indole. This effect was not observed when the chlorine was attached to the 7-position (**6**).

### 3.2. Characterization via GC-EI-MS and GC-sIR

**Figure 3:**
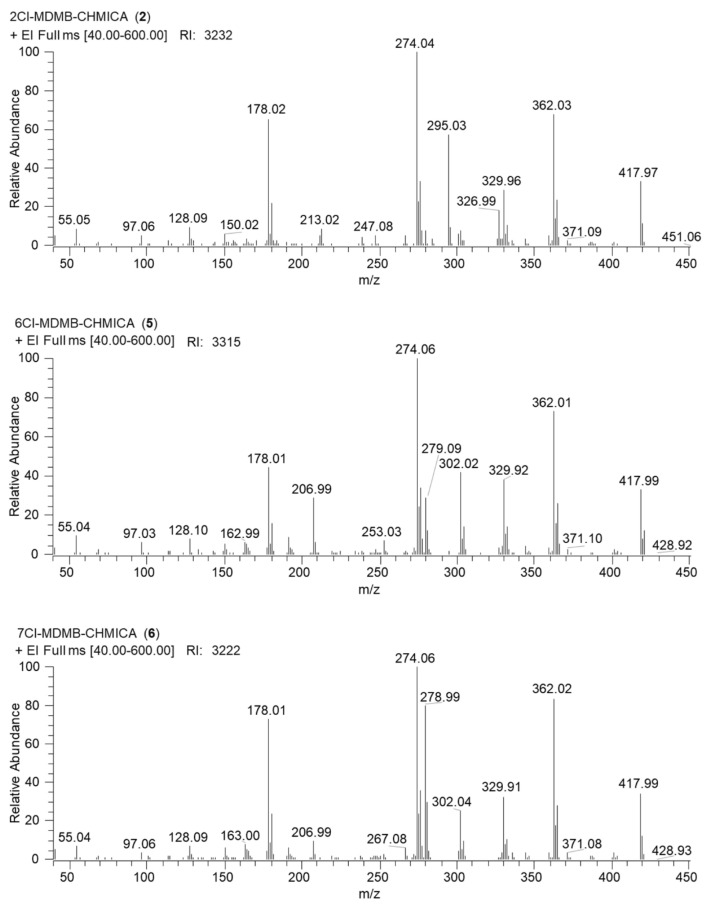
GC-EI-MS spectra of 2-Cl-, 6-Cl-, and 7-Cl-MDMB-CHMICA.

To provide data relevant to the majority of toxicological institutions, all five isomers were analyzed by GC-sIR, GC-EI-MS, UHPLC-UV/VIS, and UHPLC-ESI-MS. As GC-MS is one of the most frequently used analytical techniques to identify unknown substances, the discrimination power of this technique was investigated. The corresponding retention indices (RIs) on a TG-5MS column were **2**: 3232; **3**: 3238; **4**: 3238; **5**: 3315; and **6**: 3222. The GC-MS measurements on a DB-1 column yielded the following RI: **2**: 3142; **3**: 3261; **4**: 3258; **5**: 3214; and **6**: 3134. On a TG-5MS column and with the applied temperature gradient system, only **5** is distinguishable through the RI, whereas on a DB-1 column, **2** and **6** could be separated from **3**, **4**, and **5**. The EI-MS spectra showed very few differences in the fragmentation pattern for **2**, **3,** and **4**. However, in the fragment spectra of **5** and **6**, slightly different patterns could be observed, enabling discrimination. For comparison, the GC-EI-mass spectra of **2** (also representing **3** and **4**), **5,** and **6** are shown in Figure 3. Of special interest are the small intensities of *m*/*z* 302, 279, and 207 in the spectrum of **2** compared to those of **5** and **6**. Otherwise, very small signals for *m*/*z* 295 and 327 were observed for **5** and **6**, which are clearly present in the spectrum of **2**. Discrimination between **5** and **6** is achieved by the RI values of the chromatography and the intensity ratios of *m*/*z* 279, 207, and 178 in combination with the slightly increased signal for *m*/*z* 253 in the spectrum of **5**.

**Figure 4:**
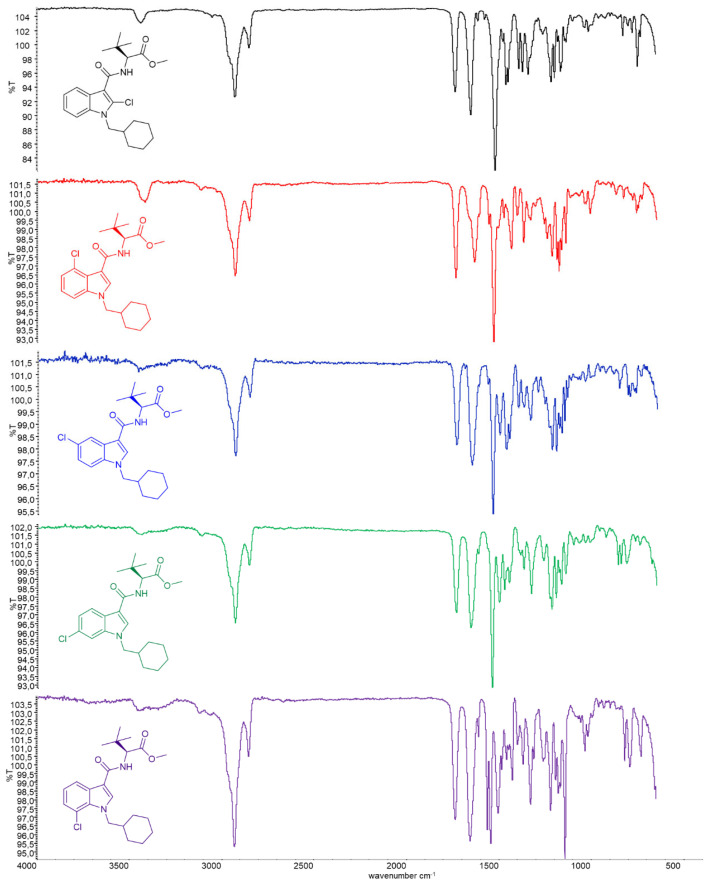
Stacked GC-sIR spectra of the five chloroindole isomers of MDMB-CHMICA.

The implementation of GC condensed phase IR analysis (GC-sIR) allows for the collection of spectral data directly from chromatographic peaks whereby the analyte is deposited cryogenically onto an IR-transparent zinc selenide disc. This technique is especially important for the analysis of mixtures, which normally occur in designer drugs containing cannabimimetic compounds. The advantage of employing this method is that IR spectra are recorded in a solid state comparable to freebase spectra with sharp distinct bands recorded under traditional conditions, for example, by using a Fourier transform attenuated total reflection (ATR) approach. Further discrimination could be achieved for all chlorinated indole derivatives by comparing the IR spectra in Figure 4. Whereas the infrared spectra of **2**, **3**, and **6** are completely different in the fingerprint region, the spectra of **4** and **5** are very similar. The spectra of **4** and **5** could however be distinguished by small but discrete differences in the absorption bands in the fingerprint region of the IR spectra. Therefore, IR is able to differentiate all synthesized chloro-analogues of MDMB-CHMICA. The individual full spectra and fingerprint zooms of all isomers can be taken from the Supplementary Materials.

### 3.3. Characterization via UHPLC-ESI-Ion Trap-MS and UHPLC-UV/VIS

**Figure 5:**
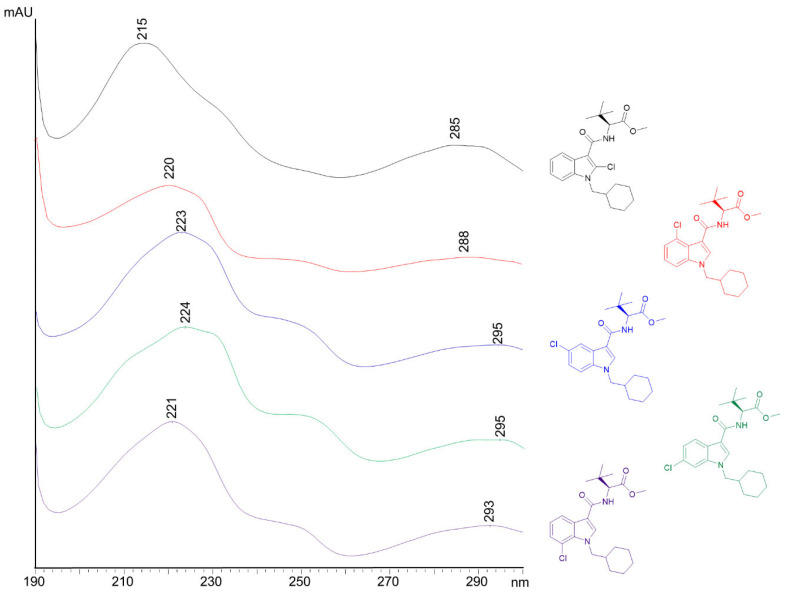
UV/VIS spectra for the five chloroindole isomers of MDMB-CHMICA.

Both the UV/VIS and MS coupled UHPLC systems were operated using the same chromatography (gradient system and column); thus, the retention times observed for all isomers on both systems were similar: **2**: 6.42 min; **3**: 5.14 min; **4**: 5.76 min; **5**: 5.80 min; and **6**: 6.40 min. Although the peaks in UHPLC were relatively sharp, it was not possible to distinguish between **2** and **6** or between **4** and **5**. The ion trap MS yielded the same mass transition of *m*/*z* 419-274-178 up to MS^3^ for all isomers with no other detectable product ions. The UV/VIS spectra recorded from 190 nm to 300 nm for all five isomers are shown in Figure 5. Small differences could be observed, especially through a shift in the maximum absorbance wavelength in the shorter wavelength range (215–224 nm) and an increased absorption around 250 nm for the three isomers, **4**, **5,** and **6**. The absorption maxima for the isomers were as follows: **2**: 215 and 285 nm; **3**: 220 and 288 nm; **4**: 223 and 295 nm; **5**: 224 and 295 nm; **6**: 221 and 293 nm.

### 3.4. In Vitro hCB1 Receptor Binding Affinity Assay

The affinity to the receptors is an important pharmacological feature of drugs, together with the agonistic response, and should be determined according to the chemical structure. Contrary to EC_50_, though, K_i_ values obtained in similar studies are not dependent on the expressed level of the receptors [31]. Various methods to test the affinity to the receptors are available in the literature, with isotopic receptor binding assays being the most traditional ones. To provide data relevant for most toxicological institutions, in the present study, after a successful synthesis, all five isomers were tested for their binding affinity at hCB_1_ by radioligand binding studies, which has shown to allow successful structure–activity relationship analysis [31].

The hCB_1_ receptor binding affinities of CP 55,940 were measured and appeared in line with previously published values [31,32,33,34]. Moreover, the hCB_1_ receptor binding affinities of non-chlorinated MDMB-CHMICA were in accordance with what was already measured by Schoeder et al. [31] using a comparable assay setup (K_i_ = 0.41 ± 0.141 nM), confirming the quality of our data. Lastly, the hCB_1_ binding affinity for JWH-018 was measured and appeared entirely consistent with previous publications with a similar experimental setting [25,34]. Taken together, these results allow the confirmation of the reliability of our data concerning the newly synthesized analogues.

**Figure 6:**
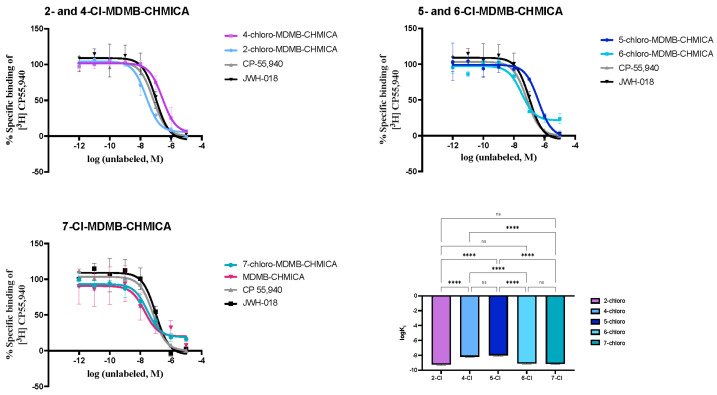
Concentration–displacement curves at the hCB_1_ receptor as obtained with the [^3^H]CP-55,940 mediated in vitro competitive binding affinity assay with the tested compounds. Data are shown as mean receptor affinity ± SEM, and experiments were performed in triplicate. ns = non significant difference; **** = significant difference with *p* = 0.0001.

The binding affinity of the synthesized test compounds **1**–**5** at the hCB_1_ receptor are reported in Table 1 and concentration–displacement curves are shown in Figure 6, compared to MDMB-CHMICA, CP 55,940, and JWH-018. All isomers, which differed only for the position of the chloro-substituent at the indole core, showed affinities in the low nanomolar range (Ki = 0.58–9.8 nM). This would suggest that the newly synthesized compound should share the effects of MDMB-CHMICA at least partially.

Nevertheless, some differences depending on the site of chlorination were also highlighted, with approximately a 16-fold variation between the most and the least affine compounds to the receptors.

Particularly, chlorination in positions 4 and 5 of the indole core was shown to reduce the hCB_1_ binding affinity compared to the non-chlorinated MDMB-CHMICA, suggesting a rather low efficacy of the compounds. A determination of the hCB_2_-receptor binding affinity would additionally allow the evaluation of whether the chlorination in positions 4 and 5 might be of interest for pharmaceutical purposes.

On the contrary, the test compounds chlorinated in positions 2, 6, and 7 retained binding affinities in the same range as reported for MDMB-CHMICA and were even lower. This would suggest that similar substitutions could be in the future exploited by illicit laboratories in the attempt to synthesize potent compounds, which are usually the most successful on the market.

On the basis of our results, the substitutions can be ranked in the following order regarding the hCB_1_ receptor binding affinity: 2-chloro > 7- and 6-chloro > 4-chloro > 5-chloro. The results of the chlorinated compounds **1**–**5** proved to be significantly different with respect to the logK_i_, as shown by the extra sum-of-squares F-test and ANOVA with post hoc Bonferroni analysis, both with (*p* = 0.0001). Particularly, 4- and 5-chloro-MDMB-CHMICA significantly differed from 2-, 6-, and 7-chloro-MDMB-CHMICA, as shown in Figure 6.

Our results accorded to what was reported for indol-3-yl-tetramethylcyclopropyl ketones, where 5-chloro substitution led to a decrease of the hCB_1_ receptor affinity (K_i_ = 512 nM) with respect to the substitution in the 6-position (K_i_ 14 nM) [14]. Eissenstat et al. [13] reported 5-substitution at the indole core with either bromine, fluorine, or methyl to be detrimental for binding and functional activity, while a high hCB_1_ binding affinity characterized synthetic cannabinoids with substitutions in the 6- and 7-indole position.

The results of Eissenstat et al. and Frost et al. also suggested that the position of substituents could be critical for binding affinity and activity [13,14]. Small substituents in the 2-position, e.g., a methyl group, appear to retain good binding activity, according to Eissenstat et al. [13], and similar K_i_ values were described for JWH-018 and JWH-007, the 2-methylated analogue of JWH-018, in competitive binding assays [35]. Accordingly, in our results, the lowest Ki value was obtained for 2-chloro-MDMB-CHMICA. Similar results were also obtained, despite a very different structure, for the analogues of the endogenous cannabinoid receptor ligand anandamide. Indeed, Lin et al. explored the requirements of the ethanolamido headgroup for the interaction with the cannabinoid receptor active site, including the effect of halogen substitution, and the highest affinity was found for 2′-chloroanandamide [36].

Our results are a further confirmation that even minor structural changes can lead to significant modifications of the pharmacological properties, and particularly affinity, as already shown, e.g., MDMB-FUBINACA compared to MMB-FUBINACA [37]. Although the potency of the compounds was not determined in the present study, this would suggest that structural modifications of the indole core might have a significant impact on the pharmaceutical characteristics of the compound.

Binding to the hCB_1_ receptor has recently been studied for MDMB-FUBINACA, a compound similar to MDMB-CHMICA. Besides the aliphatic chains, the interactions between the heterocyclic indazole core and the linker appeared as an important factor for MDMB-FUBINACA’s efficacy, with a postulated loss of efficacy with indole instead of indazole cores. It remains to be ascertained if a chloro-substitution at the indole core of MDMB-CHMICA would increase or decrease the structural rigidity, which, in MDMB-FUBINACA, likely stabilizes the active receptor conformation [38]. Given that hCB_1_ agonists could also induce conformational changes in the receptor, these effects might also trigger such changes in the receptor and therefore alter the activation and downstream signalling, as shown by Hua et al. [39].

## 4. Conclusions

UHPLC-ESI-ion trap MS is the least favourable method to distinguish between the five isomers, as the selectivity of the chromatography is only average and the soft ionization in combination with the fragmentation has no discriminating value. UV/VIS showed minor differences in absorption maxima and the overall curve; however, no distinct differences were observed that would allow an unambiguous identification of a specific isomer. GC was only capable of separating a few of the isomers. Even in combination with EI-MS, it was not possible to distinguish between **2**, **3,** and **4**. However, GC, in combination with sIR, could distinguish between all isomers in the fingerprint area of the IR spectrum. With NMR spectroscopy, it was not only possible to distinguish between the isomers, but it was also possible to identify the exact position of the chlorine on the indole via 1D and 2D experiments.

Competitive radioligand binding assays demonstrated that the position of the chlorine atom at the indole core has a substantial effect on the hCB_1_ binding affinity, with 4- and 5-chloro substitution conferring a lower hCB_1_ affinity compared to 2-, 6-, and 7-chloro substitution. It is likely that different hCB1 binding affinities are related to the electron-withdrawing effects and therefore result in different binding properties at the receptor binding site. This should be further evaluated in intrinsic activity studies of the test compounds at the hCB_1_ receptor, also taking into consideration conformational changes in the receptor. The results of these studies have caused preventive extensions in the generic definitions of the German NpSG by adding halogenated substituents in all possible locations of the core of synthetic cannabinoids. Further prospective studies of compound classes that are anticipated on the market will allow drug enforcement agencies to stay a step ahead of illicit producers.

## Figures and Tables

**Table 1:** CB_1_ receptor binding affinities of the five chloroindole derivatives of MDMB-CHMICA with the corresponding regression of the fitting curve.

Compound	IC_50_ (nM)	Binding Affinity K_i_ (nM)	Log K_i_ ± SE	Fit K_i_ (R^2^)
2-Chloro-MDMB-CHMICA	23.6	0.58	−9.24 ± 0.12	0.973
4-Chloro-MDMB-CHMICA	268.5	6.55	−8.18 ± 0.12	0.970
5-Chloro-MDMB-CHMICA	402.1	9.81	−8.01 ± 0.16	0.947
6-Chloro-MDMB-CHMICA	31.6	0.77	−9.11 ± 0.15	0.955
7-Chloro-MDMB-CHMICA	28.8	0.70	−9.15 ± 0.16	0.947
MDMB-CHMICA	21.96	0.95	−9.022 ± 0.34	0.951
CP 55,940	67.7	1.65	−8.78 ± 0.06	0.991
JWH-018	102.8	2.69	−8.57 ± 0.20	0.984

## Data Availability

Synthesis procedures and compound characterization data presented in this study are available as a single pdf file upon request from the corresponding author.

## Supplementary Materials

The following supporting information can be downloaded at https://www.mdpi.com/article/10.3390/biom14111414/s1, NMR spectra, GC-MS data, IR spectra, UV spectra, CB_1_ receptor binding data.
