# Supplementary material for: Synthesis, Analytical Characterization, and Human CB1 Receptor Binding Studies of the Chloroindole Analogues of the Synthetic Cannabinoid MDMB-CHMICA
Source: Biomolecules. 2024 Nov 6;14(11):1414. doi: 10.3390/biom14111414 (PMC11592078; doi:10.3390/biom14111414)
Supplement: Supplementary file 1 [file biomolecules-14-01414-s001.zip › biomolecules-3260575-supplementary.pdf]

Synthesis, analytical characterization and human CB_1 receptor binding studies of the chloroindole analogues of the synthetic cannabinoid MDMB-CHMICA

Sascha Münster-Müller [1,,2], Steven Hansen [3], Tobias Lucas [3], Arianna Giorgetti [4,5], Lukas Mogler [5,6], Svenja Fischmann [7], Folker Westphal [7], Volker Auwärter [5,6], Michael Pütz [1] and Till Opatz [3,*]

[1] Federal Criminal Police Office, Forensic Science Institute, Äppelallee 45, 65203 Wiesbaden, Germany.; Michael.Puetz@bka.bund.de

[2] Joint Mass Spectrometry Centre, Institute of Chemistry, Chair of Analytical Chemistry, University of Rostock, 18057 Rostock, Germany; ;SMuenster@eu.sterigenics.com

[3] Johannes Gutenberg University Mainz, Department of Chemistry, Duesbergweg 10–14, 55128 Mainz, Germany; opatz@uni-mainz.de

[4] Department of Medical and Surgical Sciences, Unit of Legal Medicine, University of Bologna, Via Irnerio 49, 40126 Bologna, Italy; arianna.giorgetti@unibo.it

[5] Institute of Forensic Medicine, Forensic Toxicology, Medical Center – University of Freiburg, Albertstr. 9, 79104 Freiburg, Germany; volker.auwaerter@uniklinik-freiburg.de

[6] Faculty of Medicine, University of Freiburg, Breisacher Str. 153, 79110 Freiburg, Germany; volker.auwaerter@uniklinik-freiburg.de

[7] State Bureau of Criminal Investigation Schleswig-Holstein, Forensic Science Institute, Mühlenweg 166, 24116 Kiel, Germany; Folker.Dr.Westphal@polizei.landsh.de

***** Correspondence: opatz@uni-mainz.de; Tel.: +49-6131-3924443

Index

NMR DATA **3**
Methyl (*S*)-2-Amino-3,3-dimethylbutanoate-hydrochloride (22) 3
2-Chloro-1*H*-indole-3-carbaldehyde (7) 6
2-Chloro-1-(cyclohexylmethyl)-1*H*-indole-3-carbaldehyde (9) 9
5-Chloro-1-(cyclohexylmethyl)-1*H*-indole-3-carbaldehyde (10) 12
1-(4-Chloro-1(cyclohexylmethyl)-1*H*-indol-3-yl)-2,2,2-trifluorethan-1-one (16) 15
1-(6-Chloro-1(cyclohexylmethyl)-1*H*-indol-3-yl)-2,2,2-trifluorethan-1-one (17) 18
1-(7-Chloro-1(cyclohexylmethyl)-1*H*-indol-3-yl)-2,2,2-trifluorethan-1-one (18) 21
2-Chloro-1-(cyclohexylmethyl)-1*H*-indole-3-carboxylic acid (11) 24
5-Chloro-1-(cyclohexylmethyl)-1*H*-indole-3-carboxylic acid (12) 27
4-Chloro-1-(cyclohexylmethyl)-1*H*-indole-3-ccarboxylic acid (19) 30
6-Chloro-1-(cyclohexylmethyl)-1*H*-indole-3-carboxylic acid (20) 33
7-Chloro-1-(cyclohexylmethyl)-1*H*-indole-3-carboxylic acid (21) 36
Methyl (*S*)-2-(2-chloro-1-(cyclohexylmethyl)-1*H*-indole-3-carboxamido)-3,3-dimethylbutanoate (2-Chloro-MDMB-CHMICA) (2) 39
Methyl (*S*)-2-(4-chloro-1-(cyclohexylmethyl)-1*H*-indole-3-carboxamido)-3,3-dimethylbutanoate (4-Chloro-MDMB-CHMICA) (3) 42
Methyl (*S*)-2-(5-chloro-1-(cyclohexylmethyl)-1*H*-indole3-carboxamido)-3,3-dimethylbutanoate (5-Chloro-MDMB-CHMICA) (4) 45
Methyl (*S*)-2-(6-chloro-1-(cyclohexylmethyl)-1*H*-indole-3-carboxamido)-3,3-dimethylbutanoate (6-Chloro-MDMB-CHMICA) (5) 48
Methyl (*S*)-2-(7-chloro-1-(cyclohexylmethyl)-1*H*-indole-3-carboxamido)-3,3-dimethylbutanoate (7-Chloro-MDMB-CHMICA) (6) 51
GC-MS SPECTRA **54**
IR SPECTRA **56**
UV SPECTRA **63**
CB_1 RECEPTOR BINDING ASSAY .... FEHLER! TEXTMARKE NICHT DEFINIERT.

This supplementary information shows the recorded spectra of 2-Cl-, 4-Cl-, 5-Cl-, 6-Cl- and 7-Cl-MDMB-CHMICA via NMR, GC-MS, GC-sIR and UHPLC-UV/VIS and the corresponding graphs for competitive receptor binding studies. Additionally, all intermediates of the corresponding syntheses were characterized via NMR.

NMR Data

Figure S-1 to S-85 show the ^{1}H and ^{13}C NMR, COSY, HSQC and HMBC spectra of 2-Cl-, 4-Cl-, 5-Cl-, 6-Cl- and 7-Cl-MDMB-CHMICA and their corresponding intermediates while synthesis.

Methyl (*S*)-2-Amino-3,3-dimethylbutanoate-hydrochloride (22)

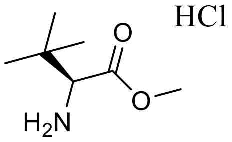

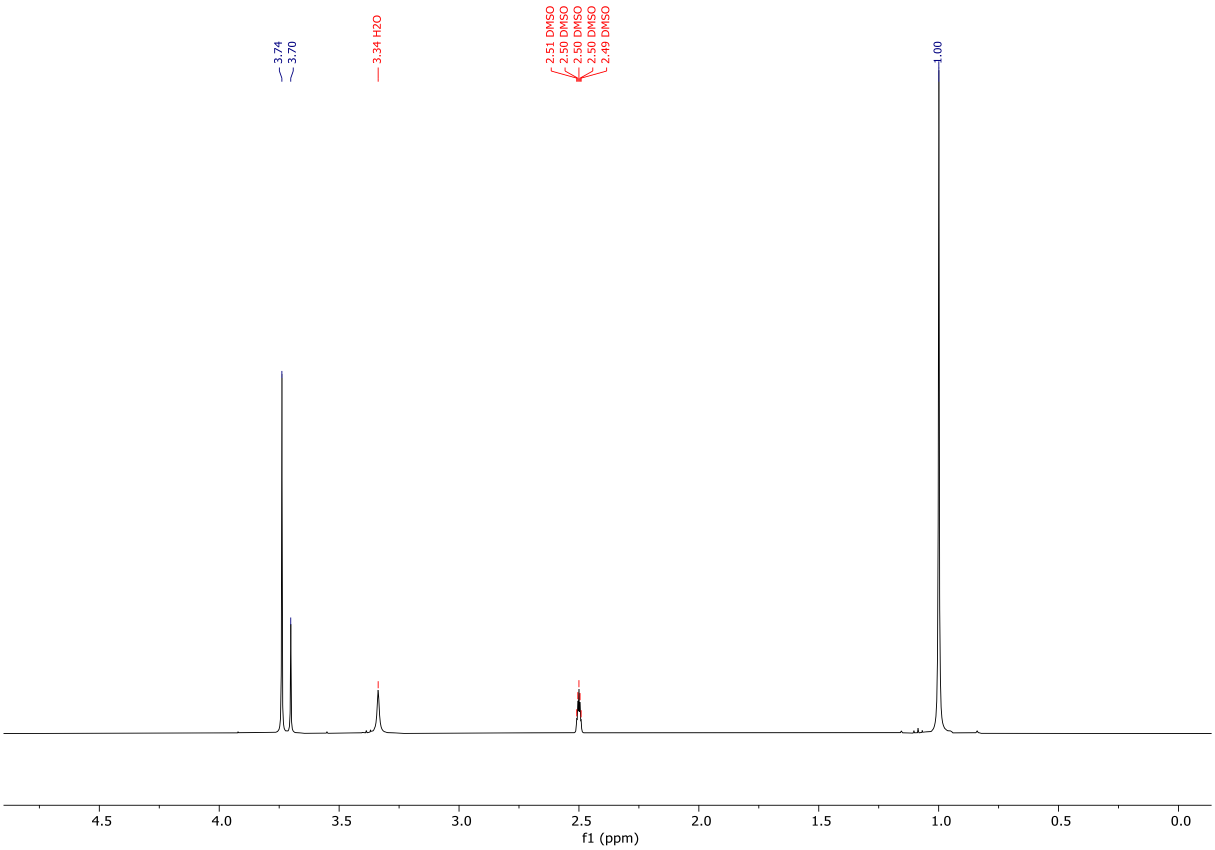

Figure S-1: ^{1}H-NMR of 22 (400 MHz, DMSO-d_6)

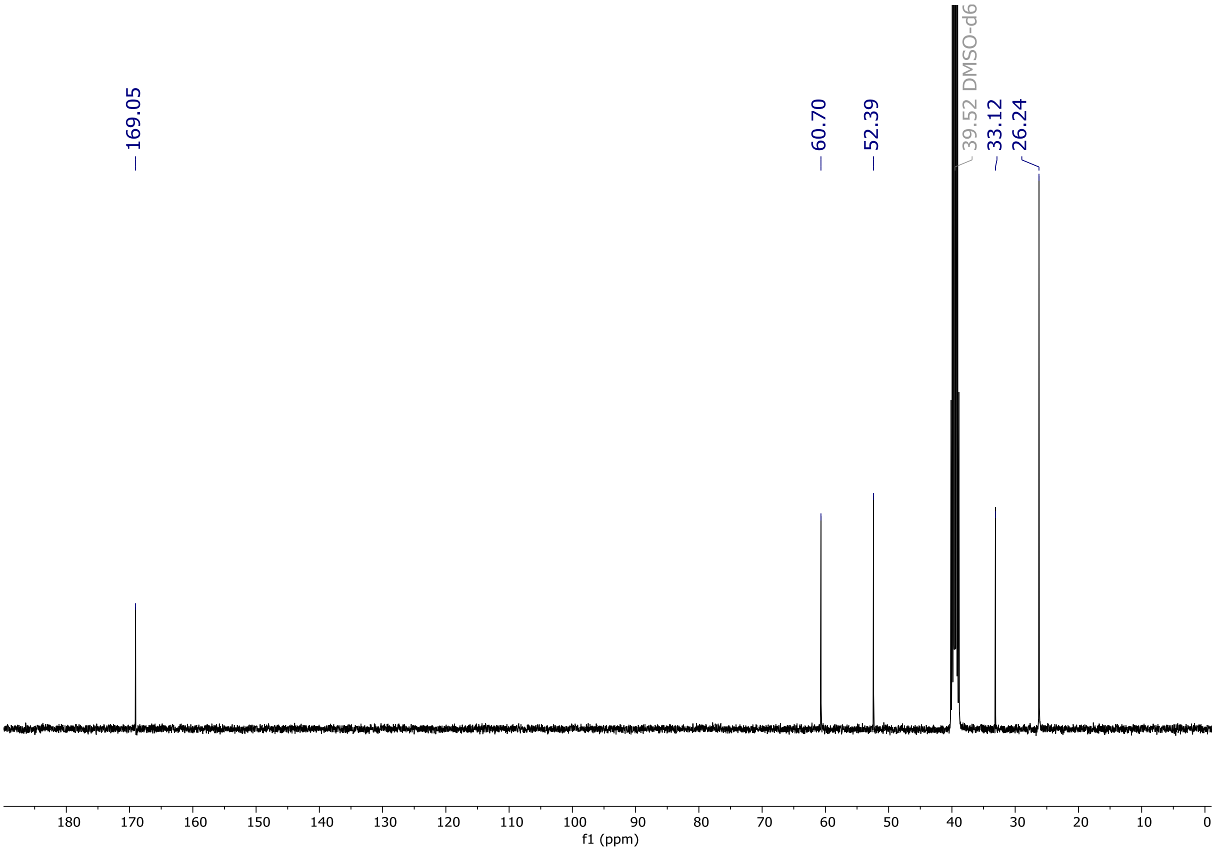

Figure S-2: ^{13}C-NMR of 22 (100.6 MHz, DMSO-*d$_6$*)

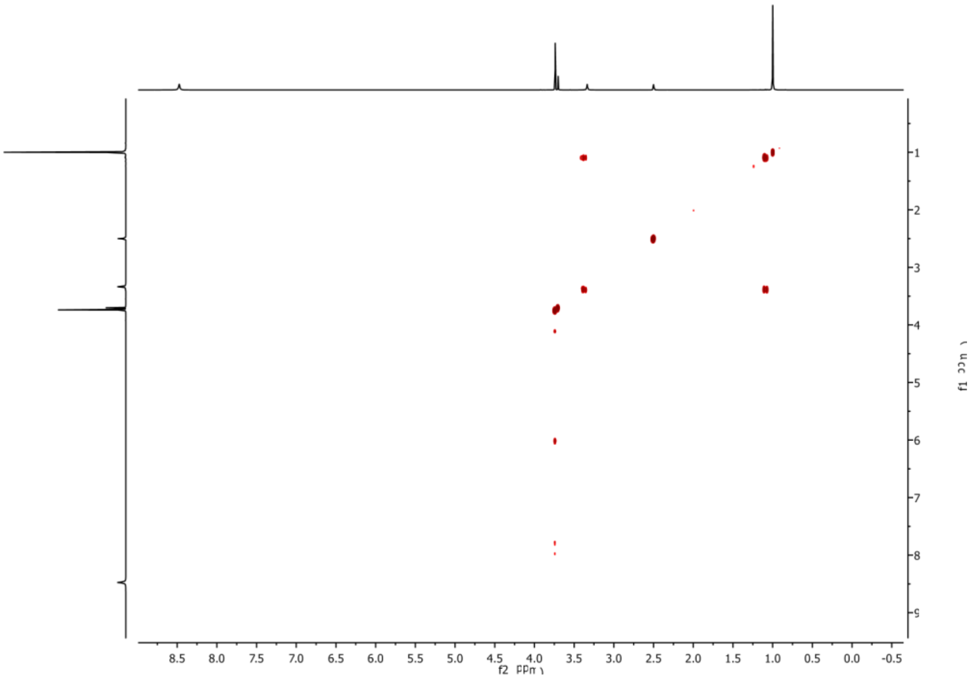

Figure S-3: COSY of 22 (400 MHz, DMSO-*d$_6$*):

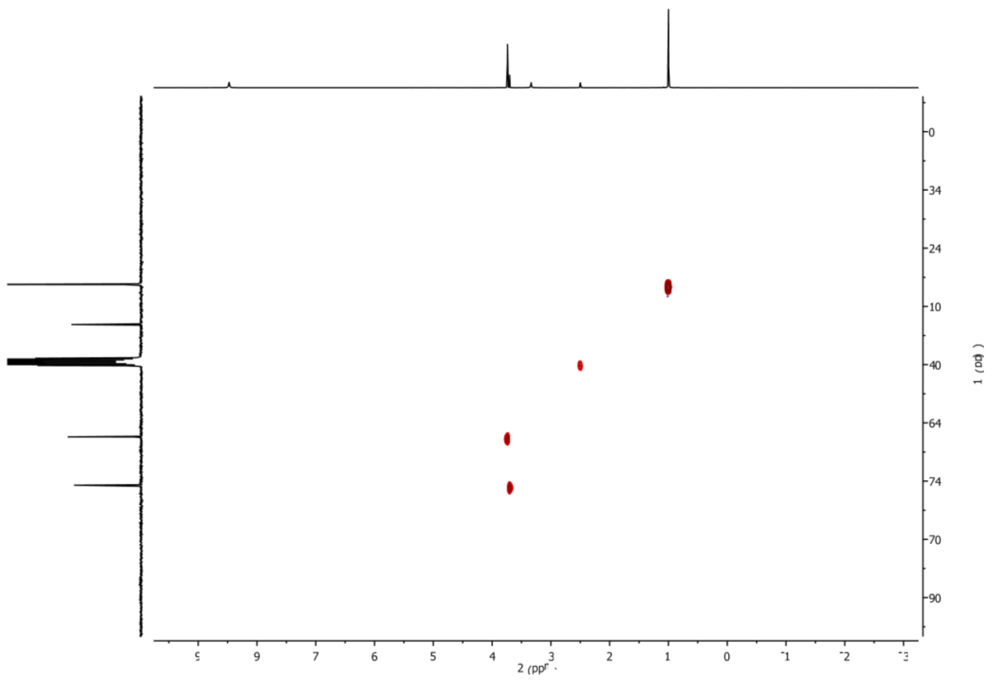

Figure S-4: HSQC of 22 (100.6 MHz, DMSO-*d*$_6$)

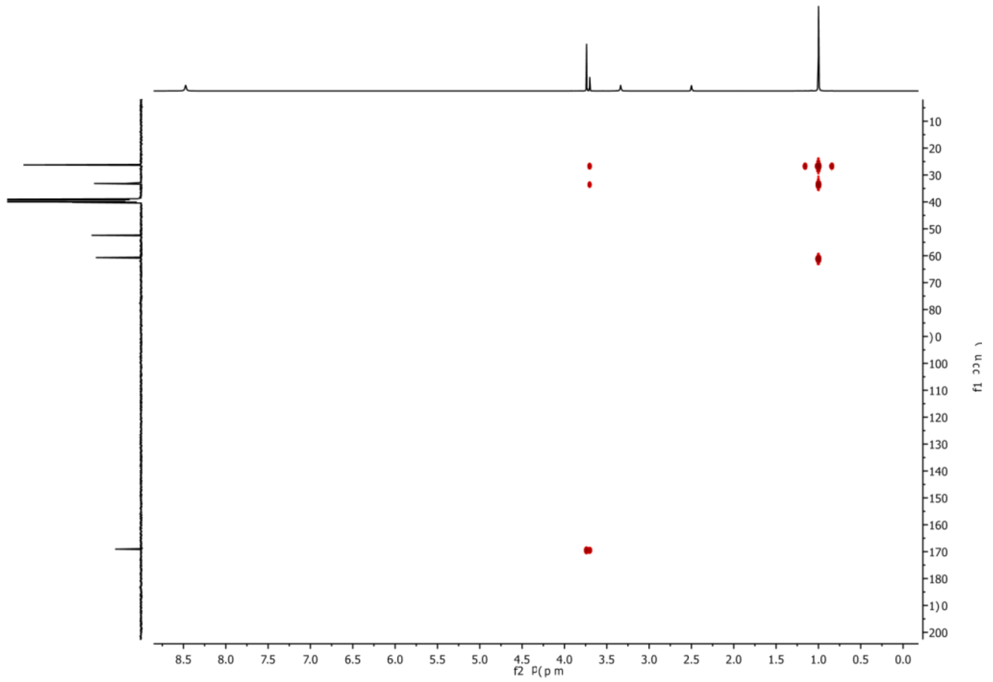

Figure S-5: HMBC of 22 (100.6 MHz, DMSO-*d*$_6$)

2-Chloro-1*H*-indole-3-carbaldehyde (7)

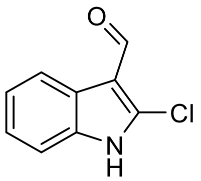

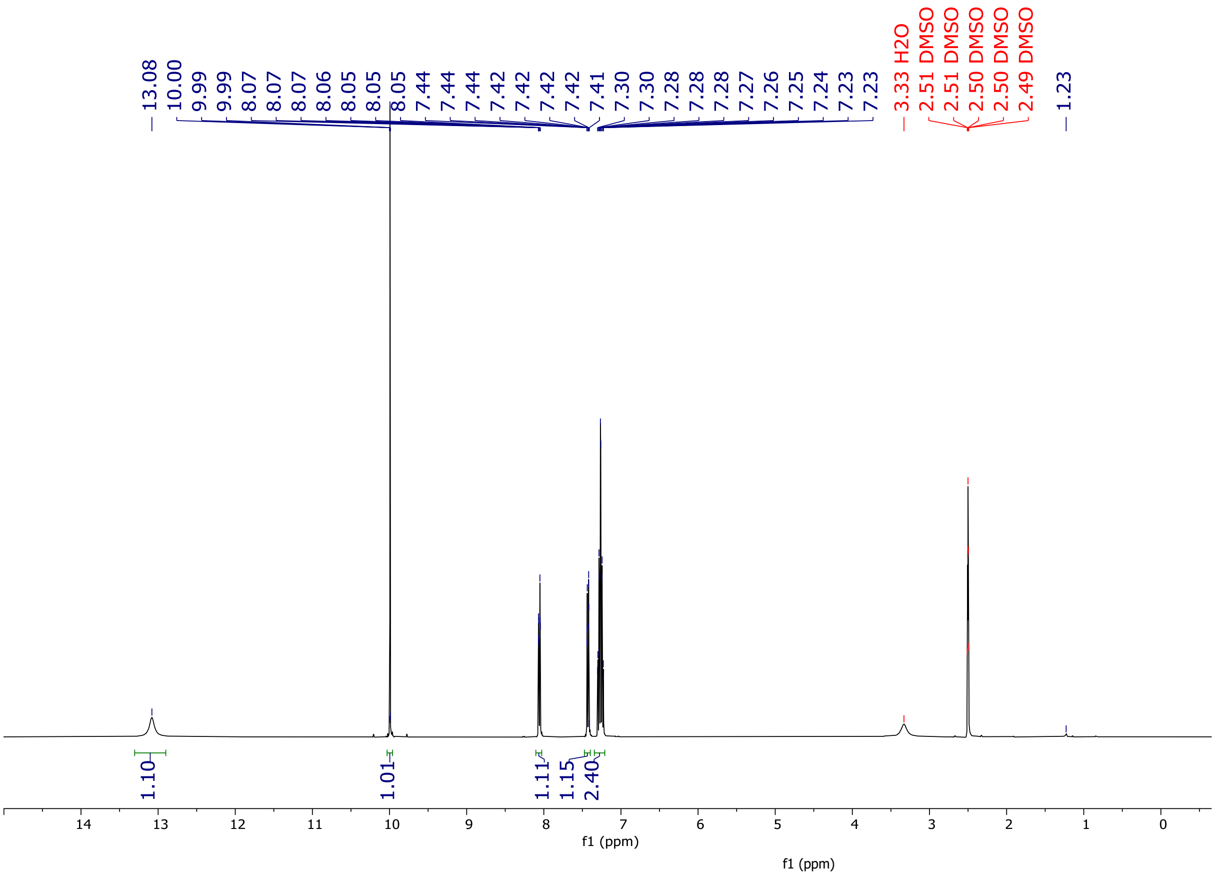

Figure S-6: ^{1}H-NMR of 7 (400 MHz, DMSO-d_6)

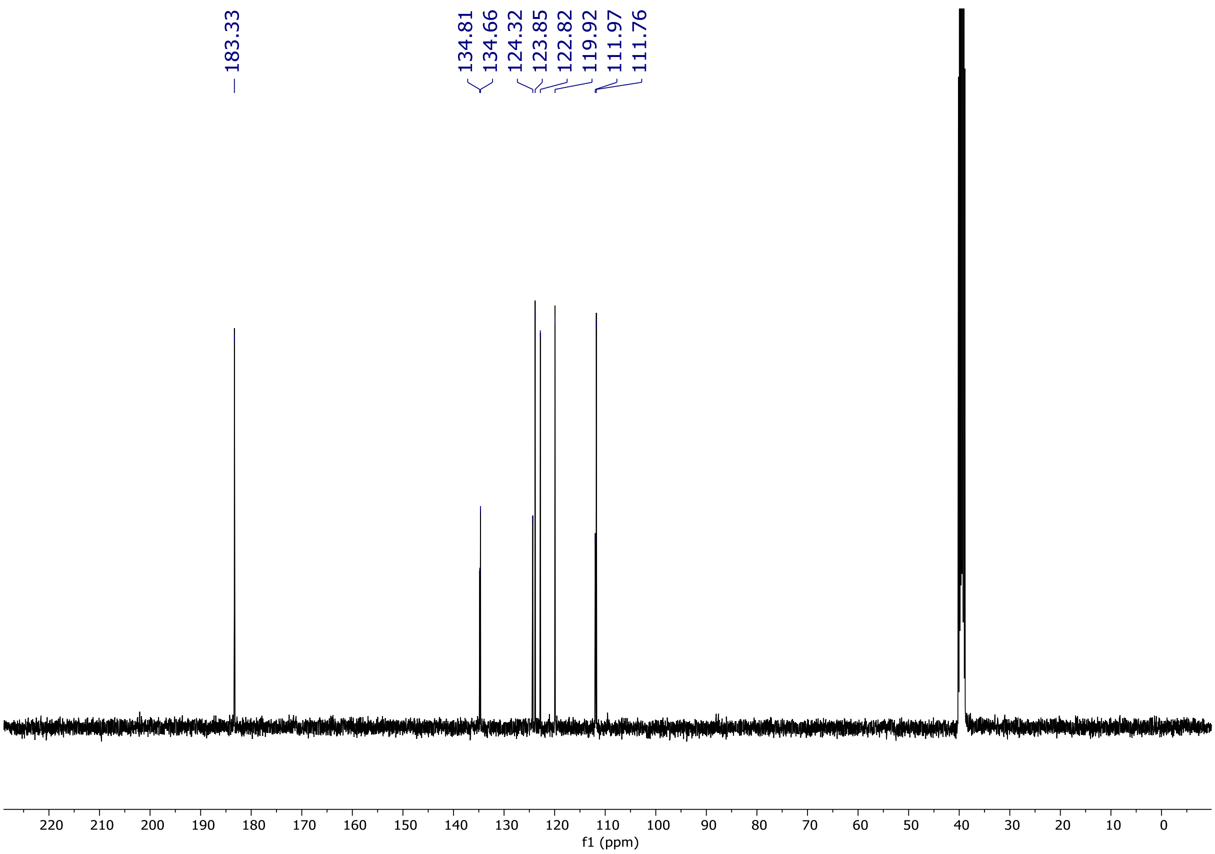

Figure S-7: ^{13}C-NMR of 7 (100.6 MHz, DMSO-*d6*)

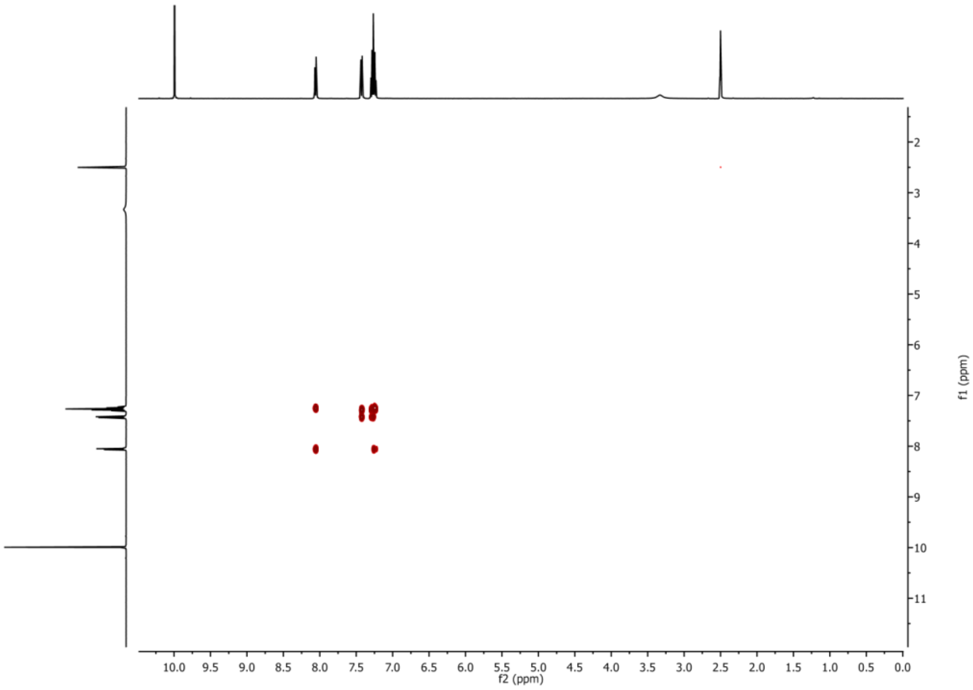

Figure S-8: COSY of 7 (400 MHz, DMSO-d_6)

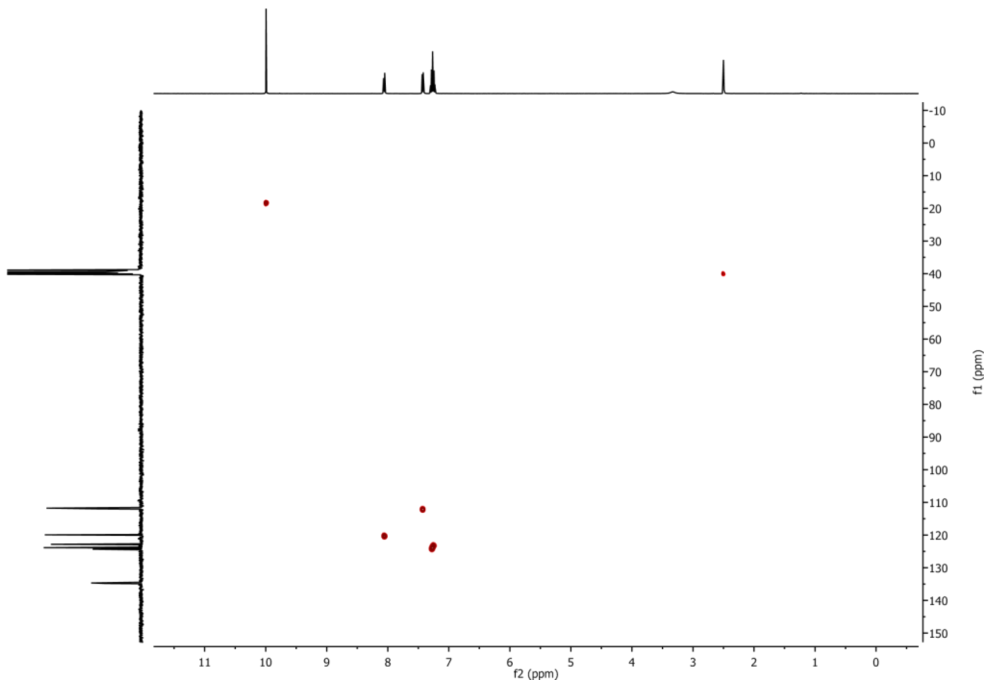

Figure S-9: HSQC of 7 (100.6 MHz, DMSO-d_6)

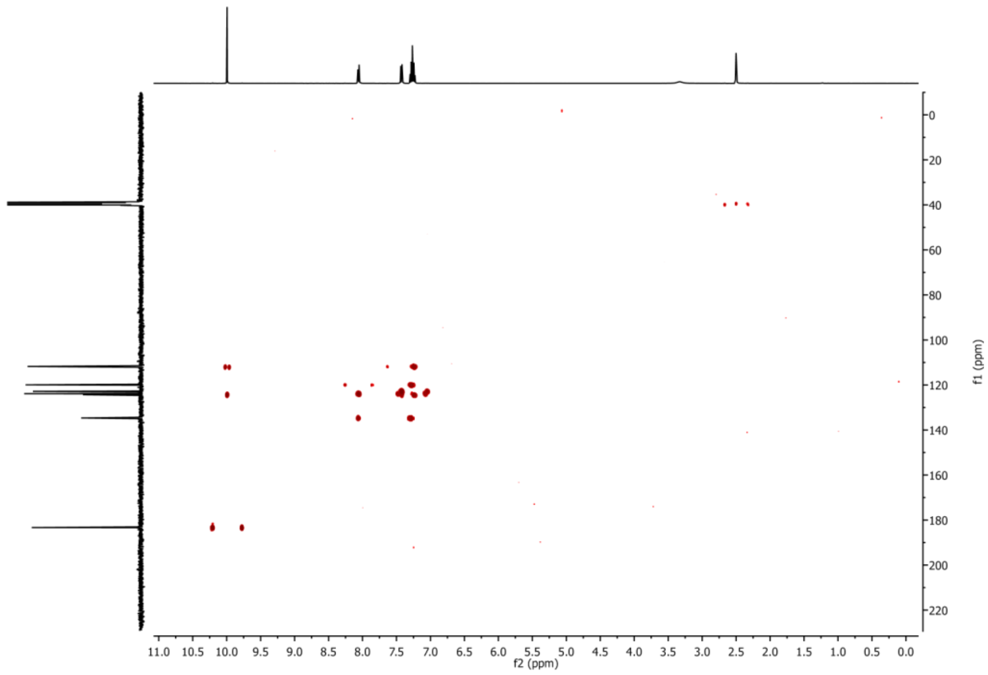

Figure S-10: HMBC of 7 (100.6 MHz, DMSO-d_6)

2-Chloro-1-(cyclohexylmethyl)-1*H*-indole-3-carbaldehyde (9)

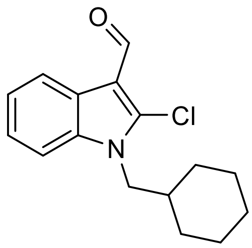

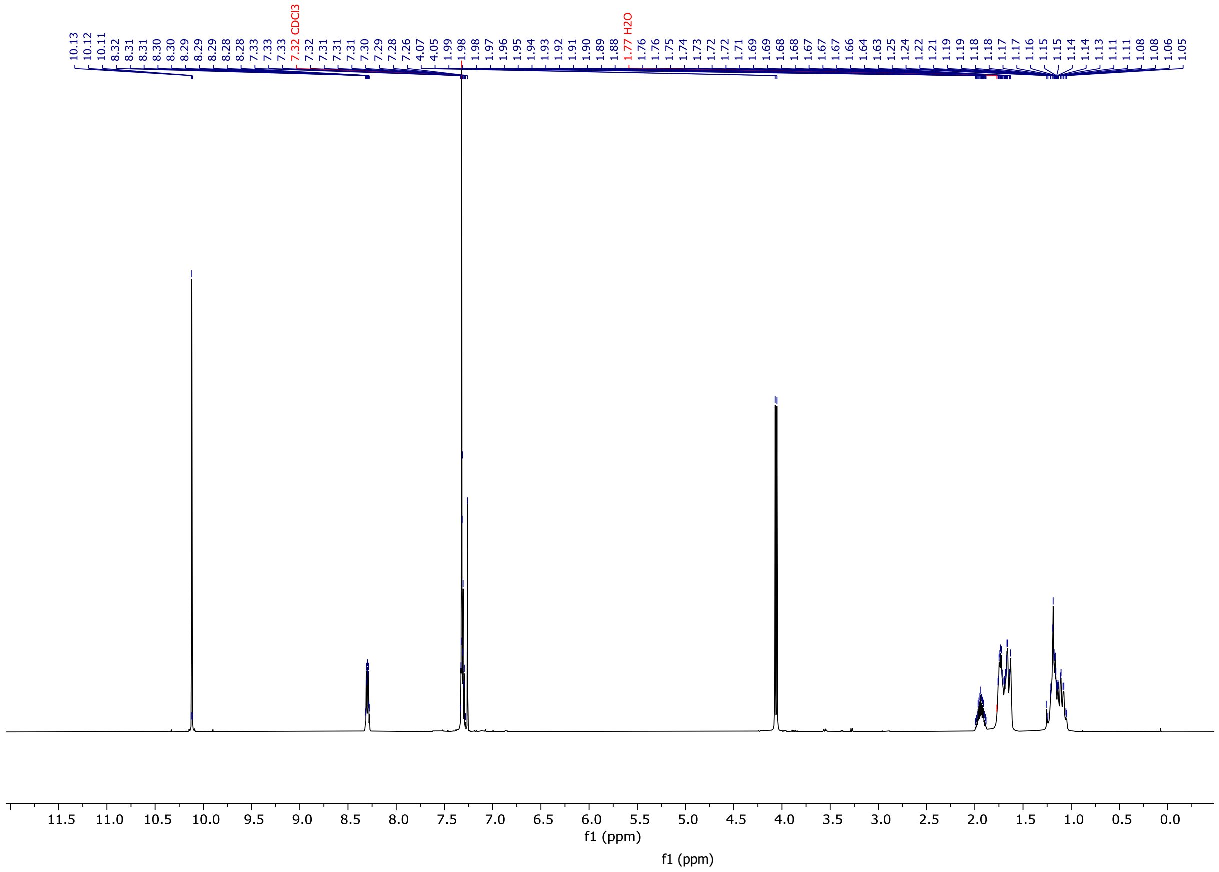

Figure S-11: ^{1}H-NMR of 9 (400 MHz, $CDCl_3$)

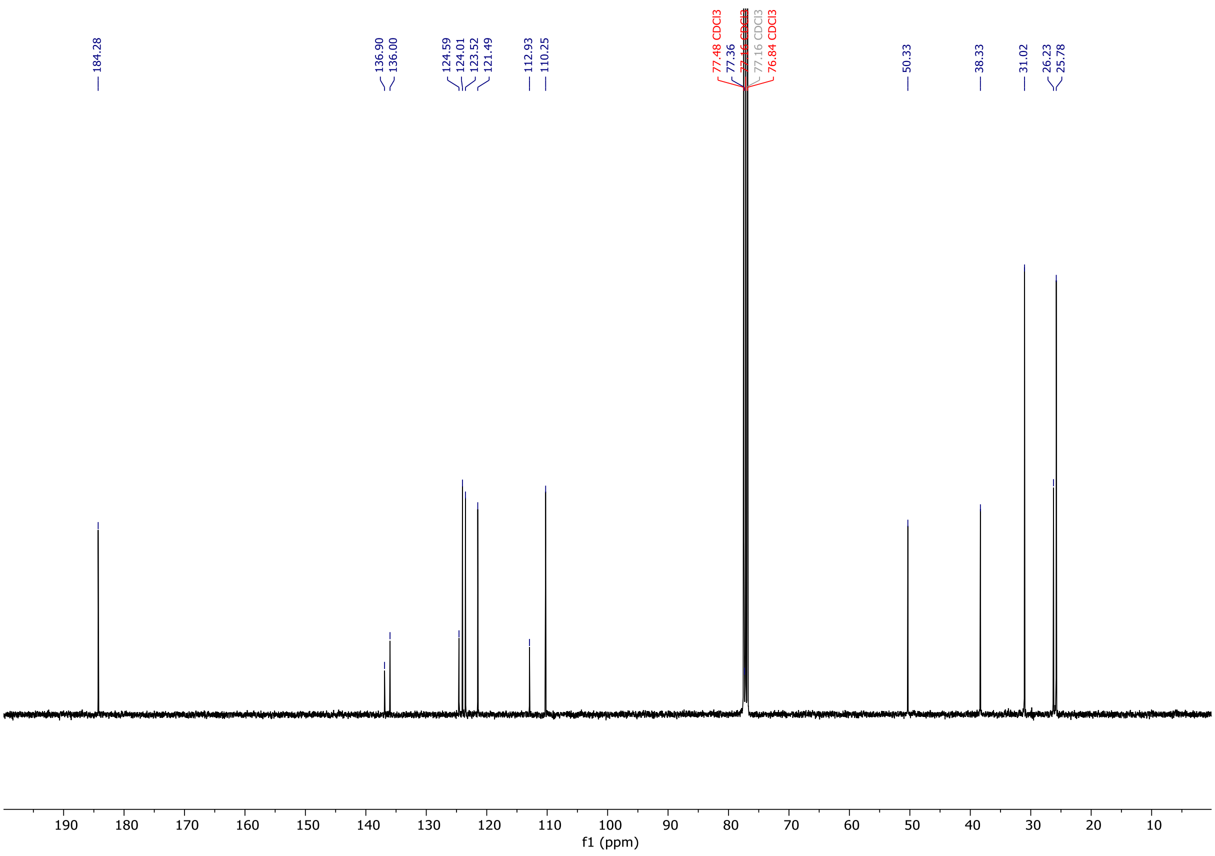

Figure S-12: ^{13}C-NMR of 9 (100.6 MHz, CDCl3)

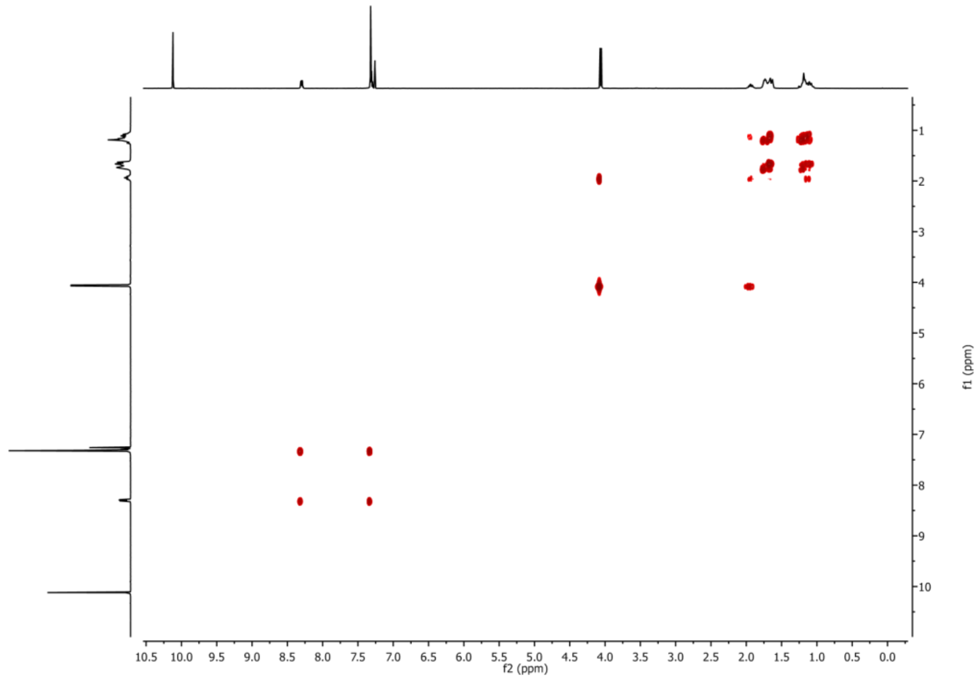

Figure S-13: COSY of 9 (400 MHz, $CDCl_3$)

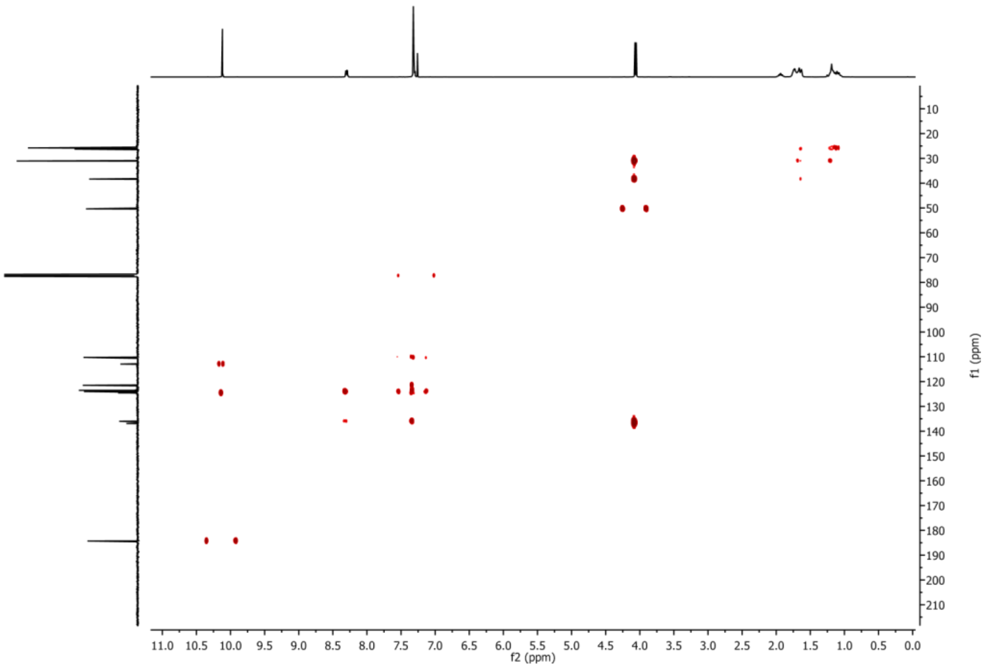

Figure S-14: HSQC of 9 (100.6 MHz, CDCl$_3$)

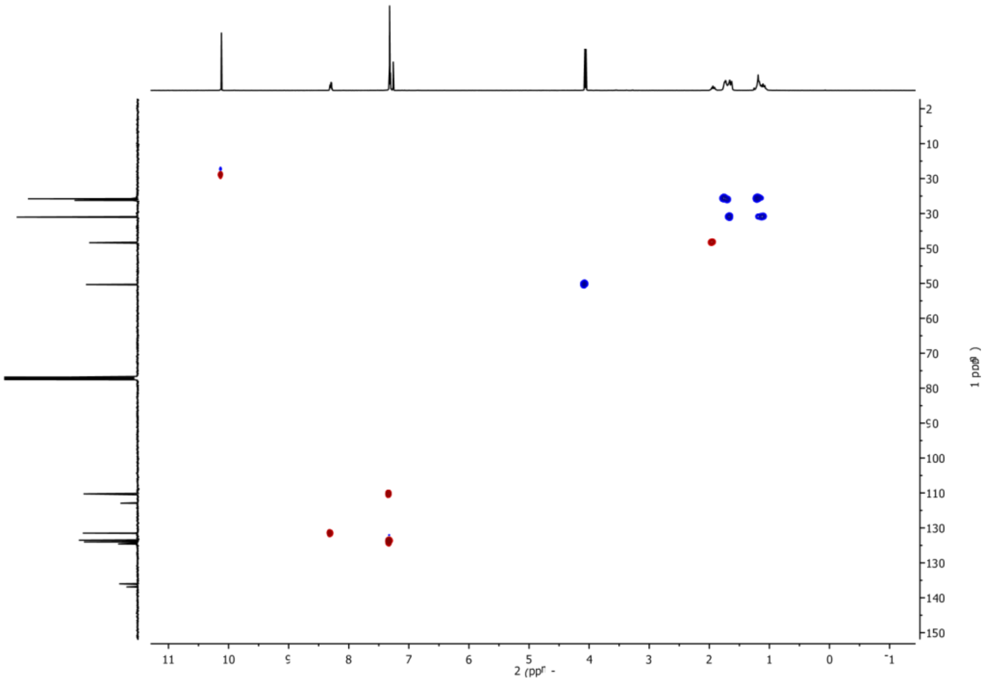

Figure S-15: HMBC of 9 (100.6 MHz, CDCl$_3$)

5-Chloro-1-(cyclohexylmethyl)-1*H*-indole-3-carbaldehyde (10)

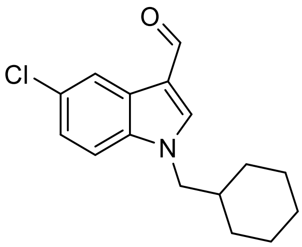

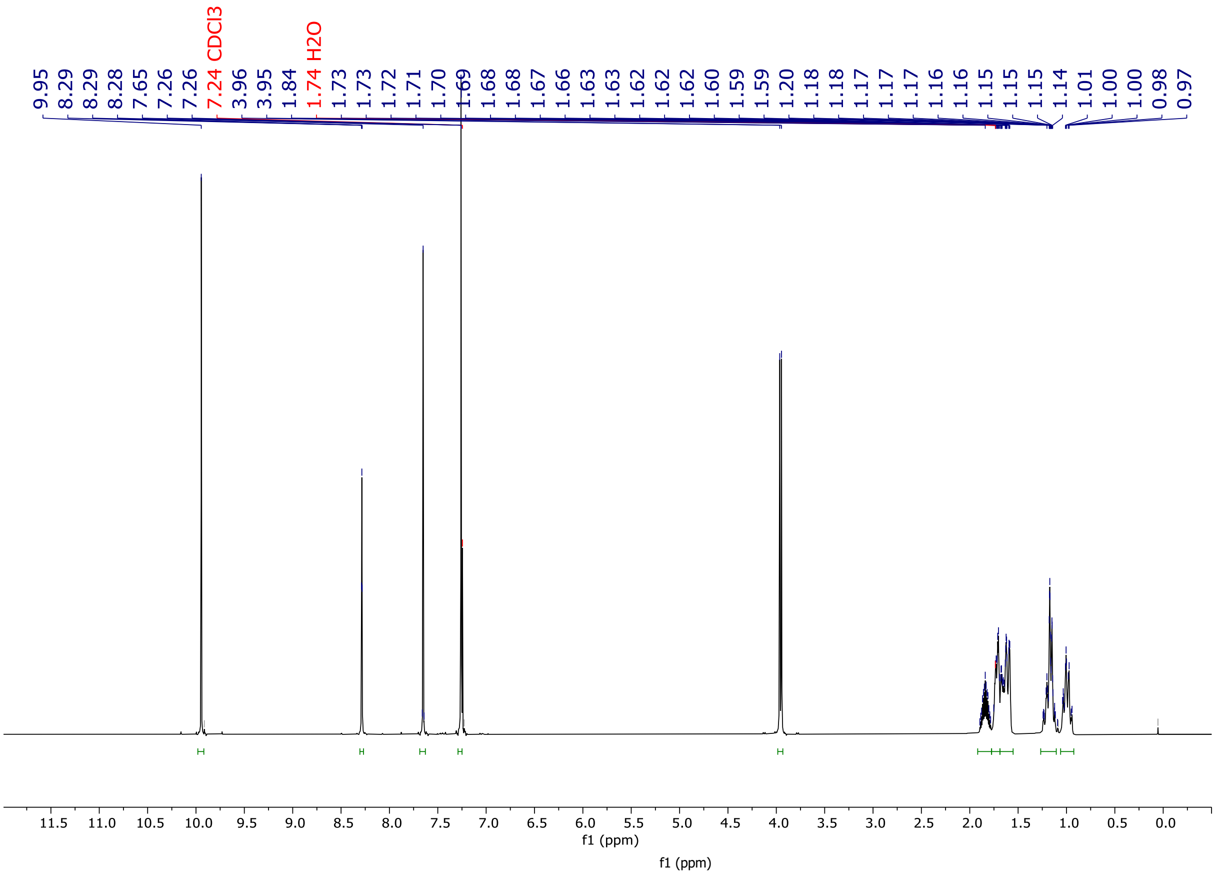

Figure S-16: ^{1}H-NMR of 10 (400 MHz, CDCl3)

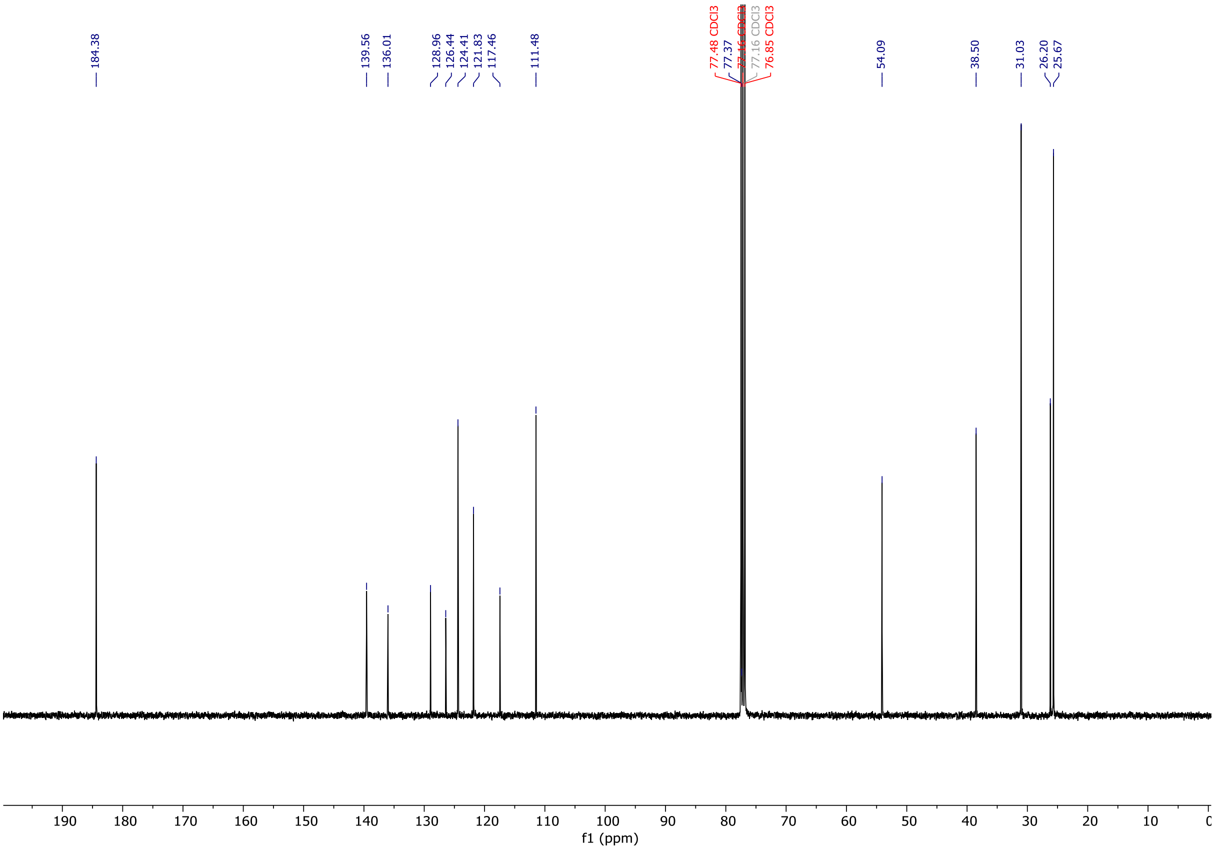

Figure S-17: ^{13}C-NMR of 10 (100.6 MHz, CDCl3)

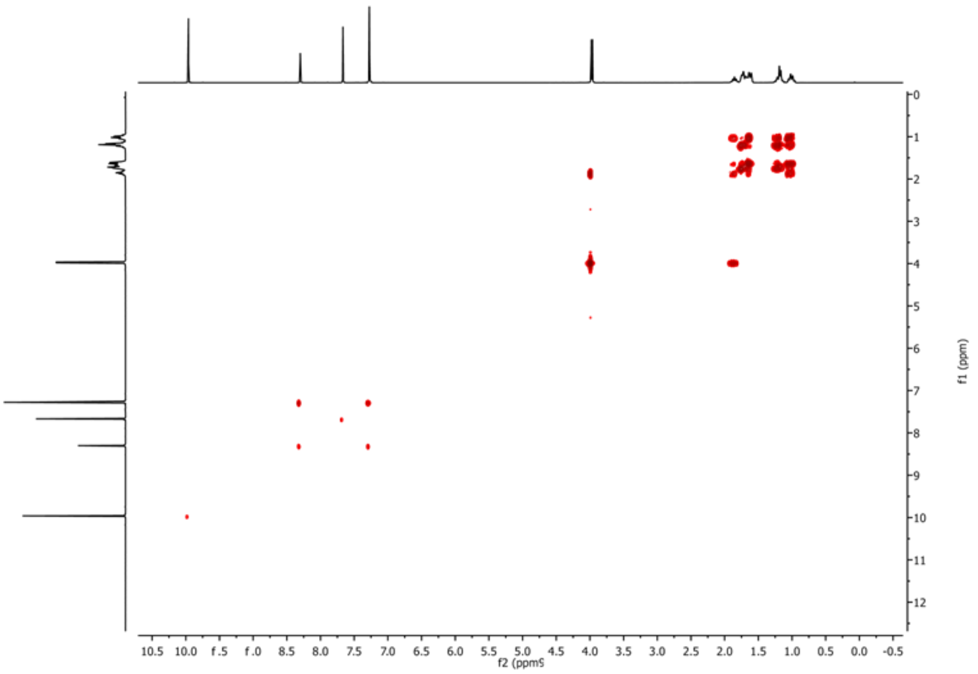

Figure S-18: COSY of 10 (400 MHz, $CDCl_3$)

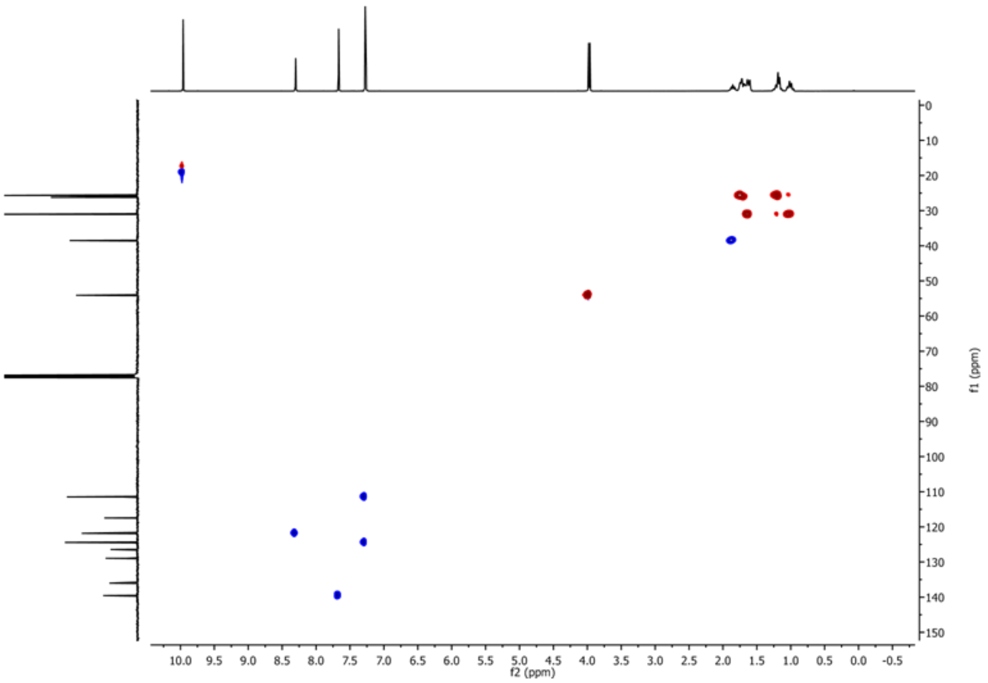

Figure S-19: HSQC of 10 (100.6 MHz, $CDCl_3$)

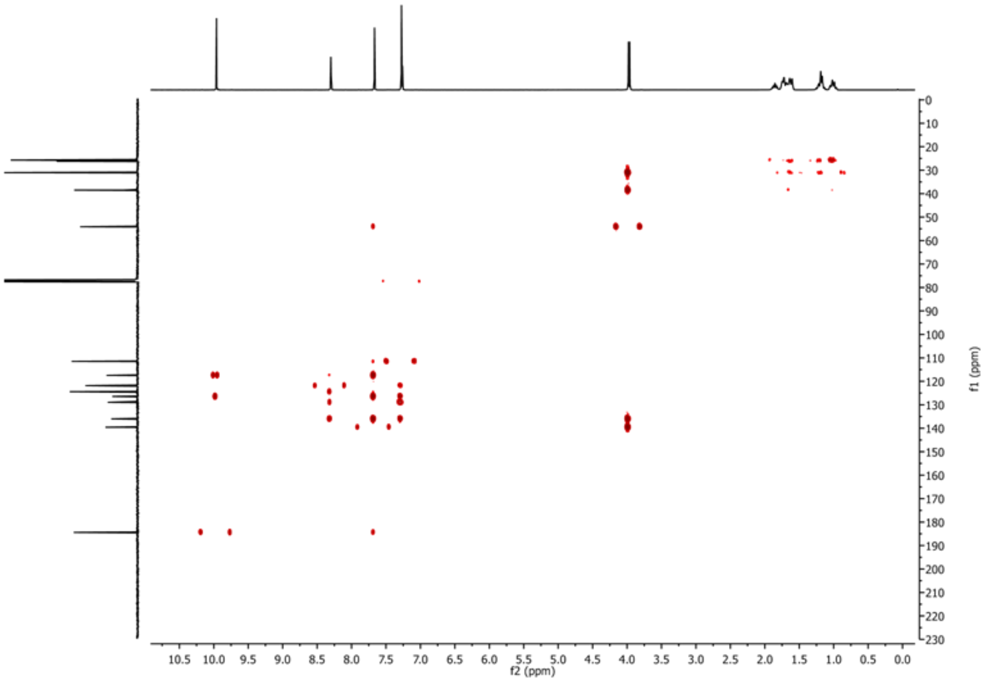

Figure S-20: HMBC of 10 (100.6 MHz, $CDCl_3$)

1-(4-Chloro-1(cyclohexylmethyl)-1*H*-indol-3-yl)-2,2,2-trifluorethan-1-one (16)

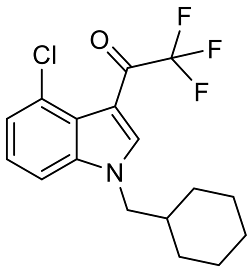

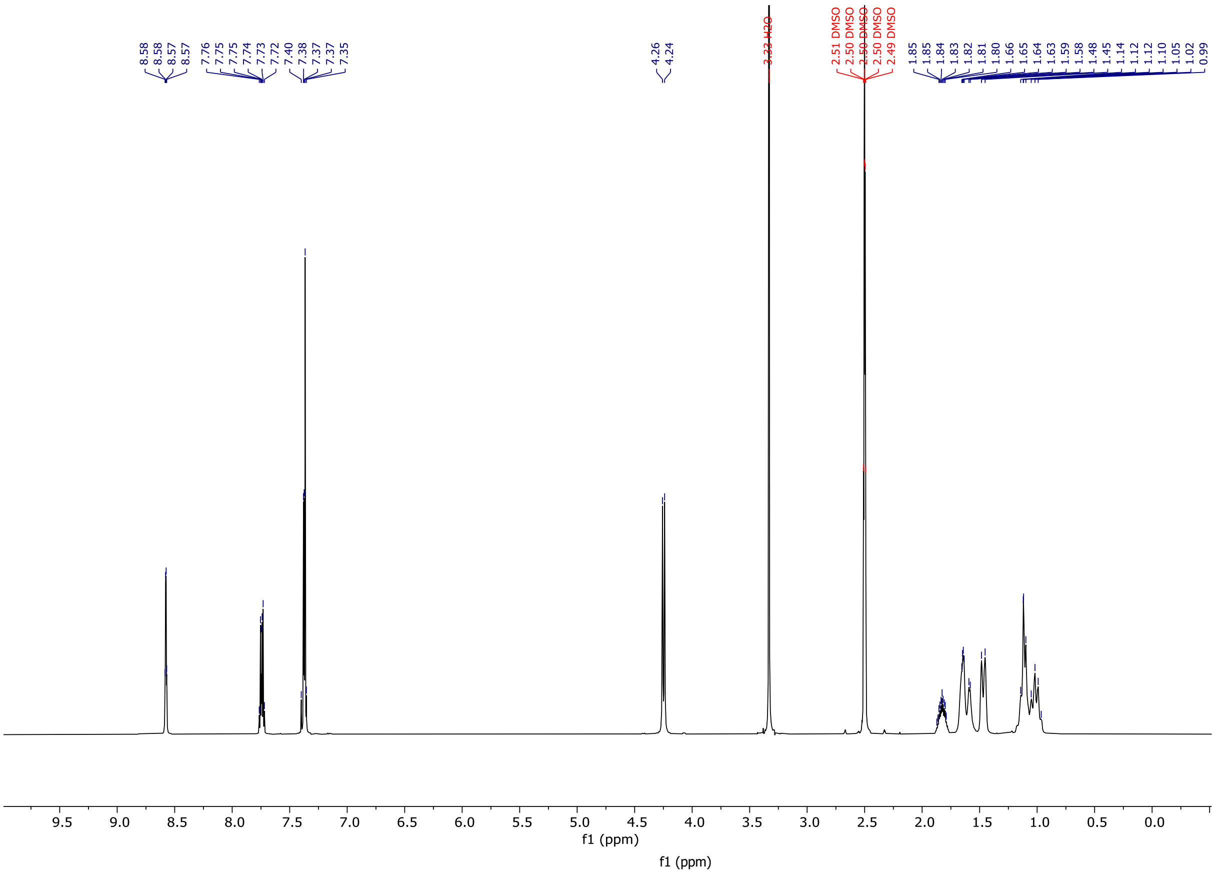

Figure S-21: ^{1}H-NMR of 16 (400 MHz, DMSO-d_6)

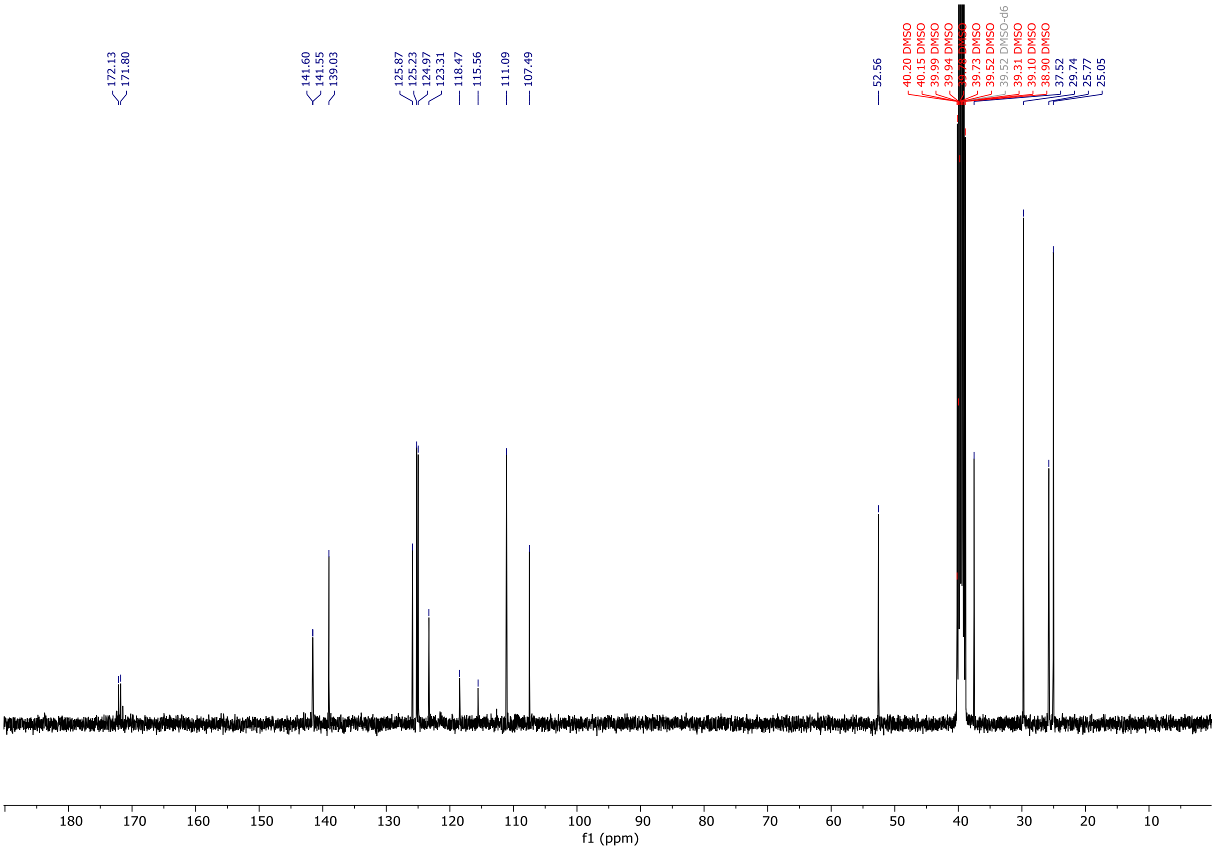

Figure S-22: ^{13}C-NMR of 16 (100.6 MHz, DMSO-*d6*)

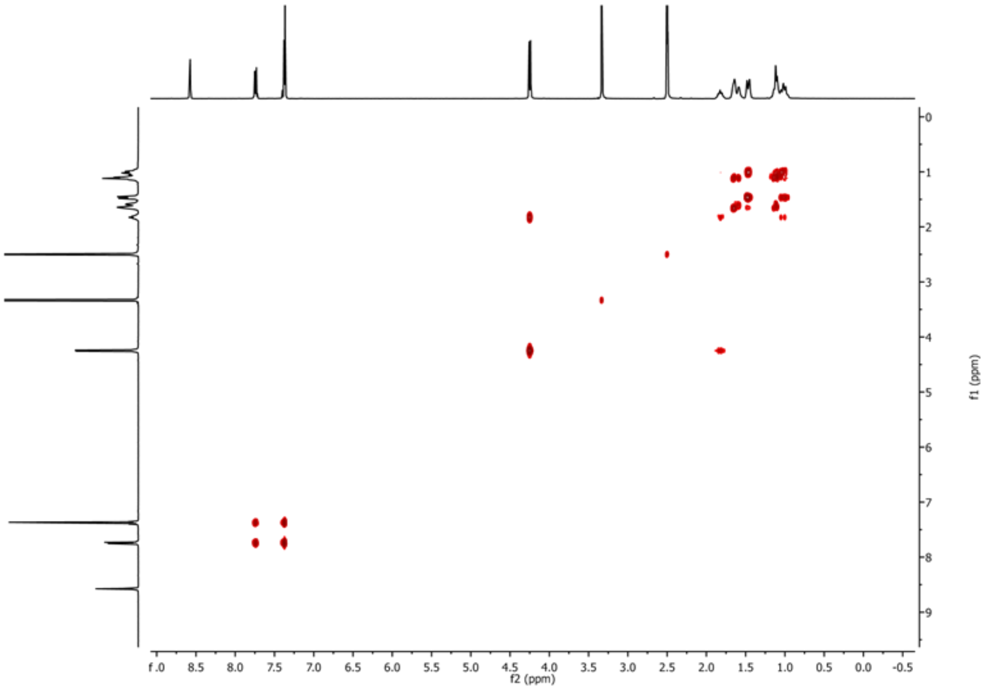

Figure S-23: COSY of 16 (400 MHz, DMSO-*d*$_6$)

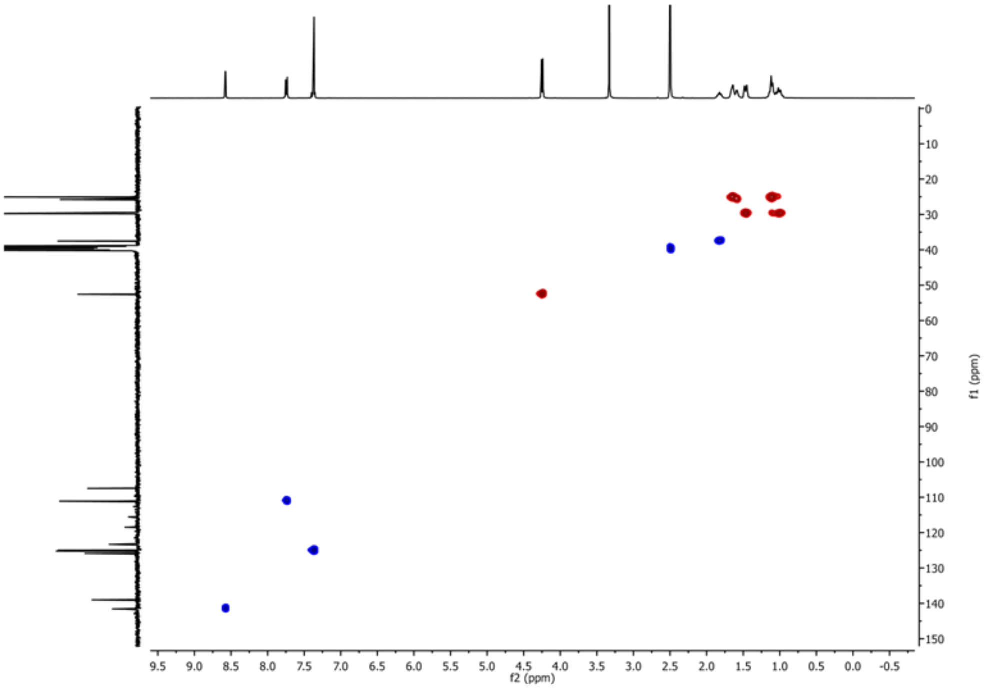

Figure S-24: HSQC of 16 (100.6 MHz, DMSO-d_6)

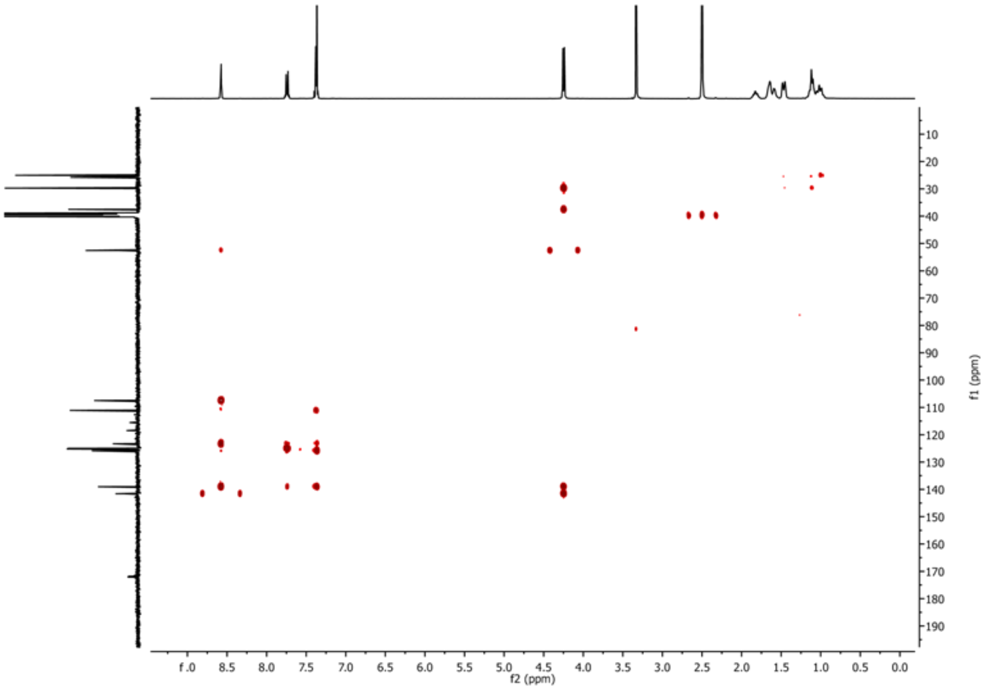

Figure S-25: HMBC of 16 (100.6 MHz, DMSO-d_6)

1-(6-Chloro-1(cyclohexylmethyl)-1*H*-indol-3-yl)-2,2,2-trifluorethan-1-one (17)

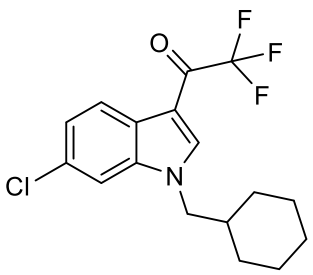

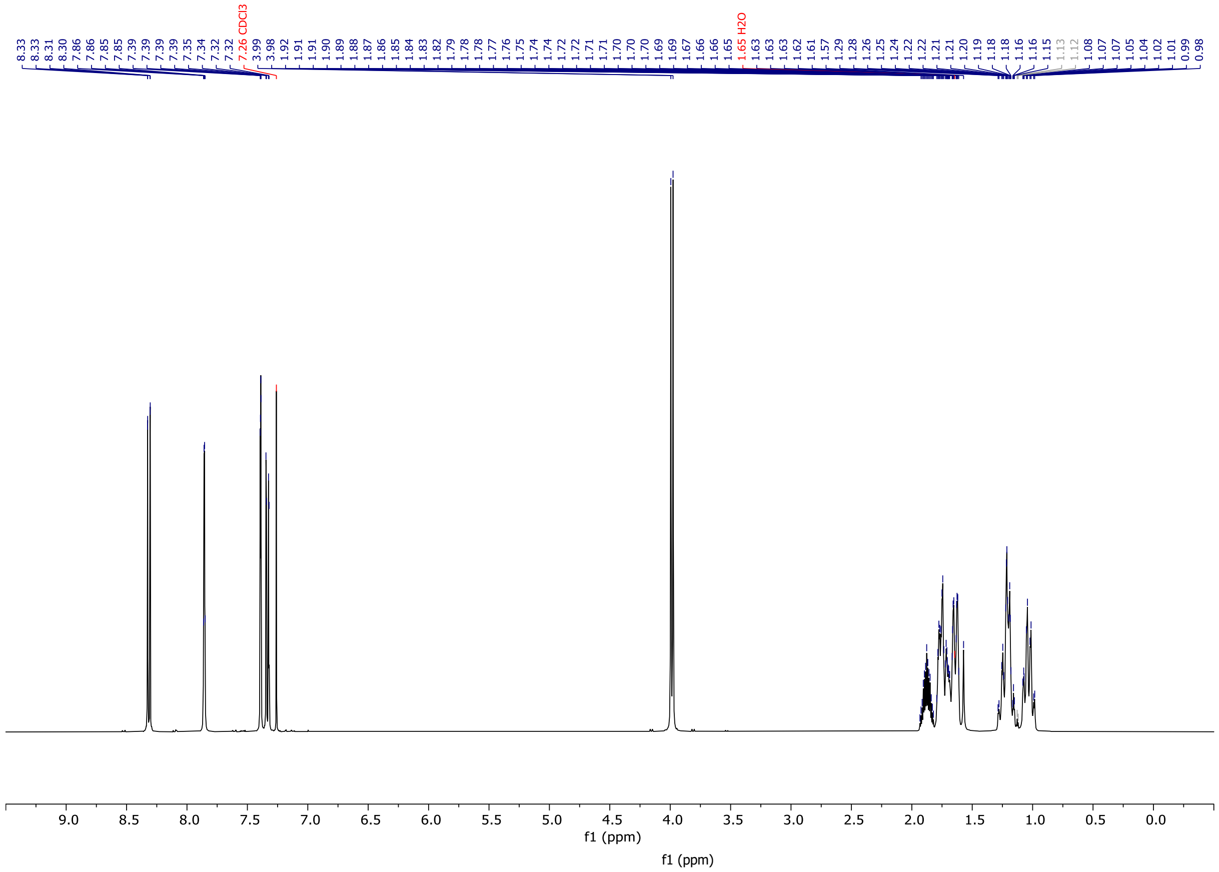

Figure S-26: ^{1}H-NMR of 17 (400 MHz, $CDCl_3$)

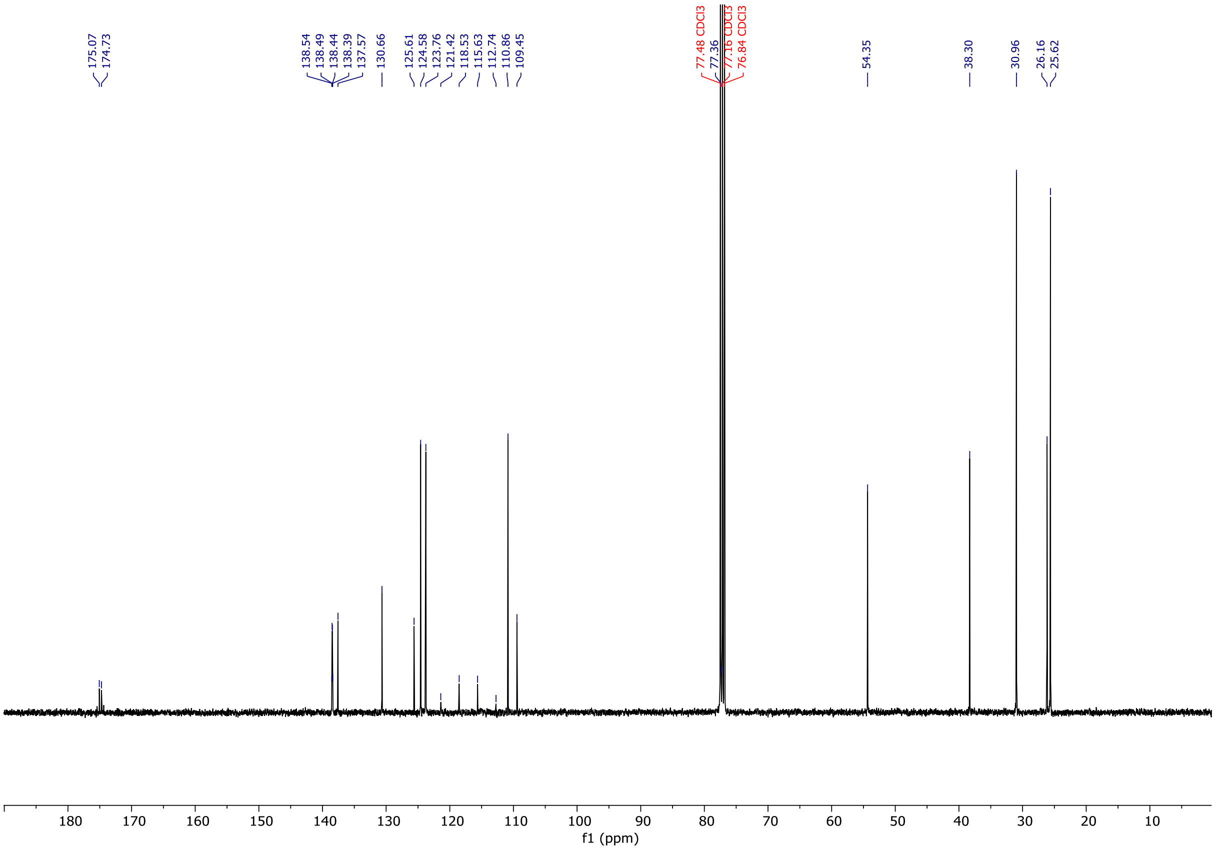

Figure S-27: ^{13}C-NMR of 17 (100.6 MHz, CDCl3)

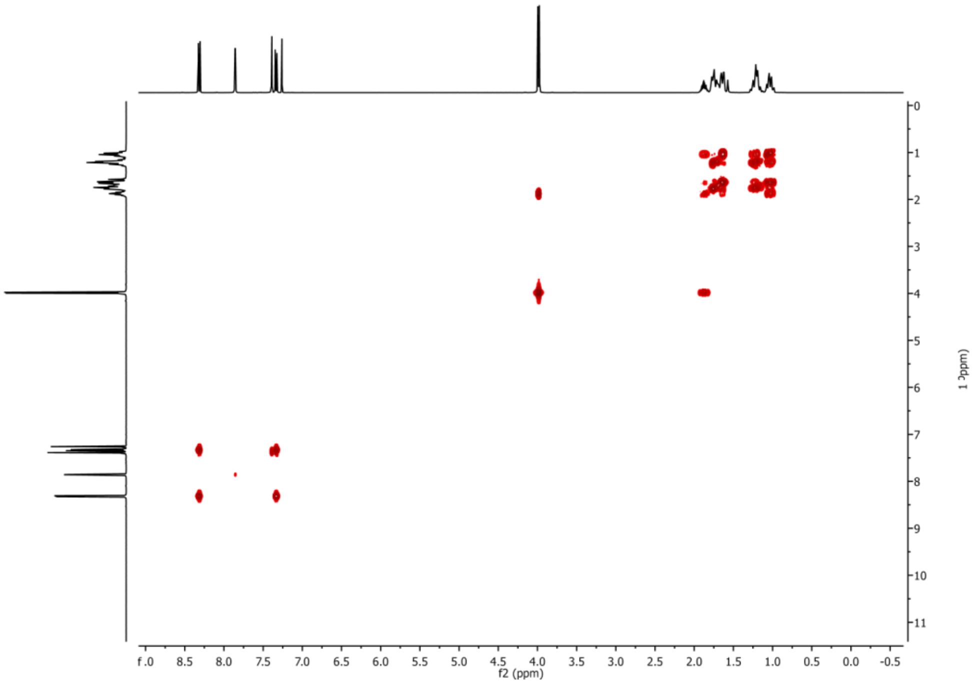

Figure S-28: COSY of 17 (400 MHz, $CDCl_3$)

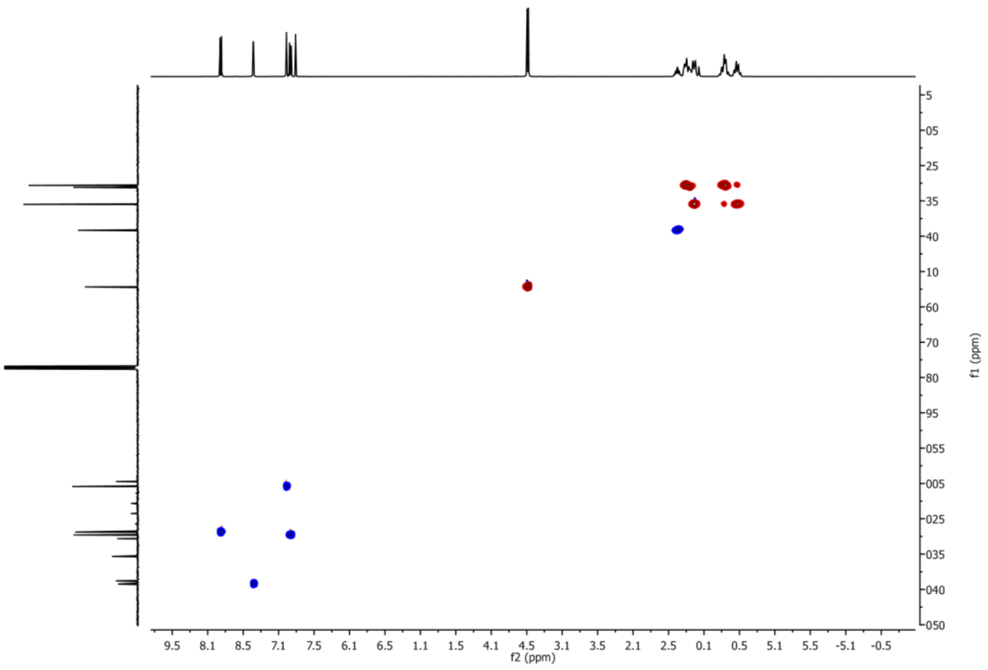

Figure S-29: HSQC of 17 (100.6 MHz, $CDCl_3$)

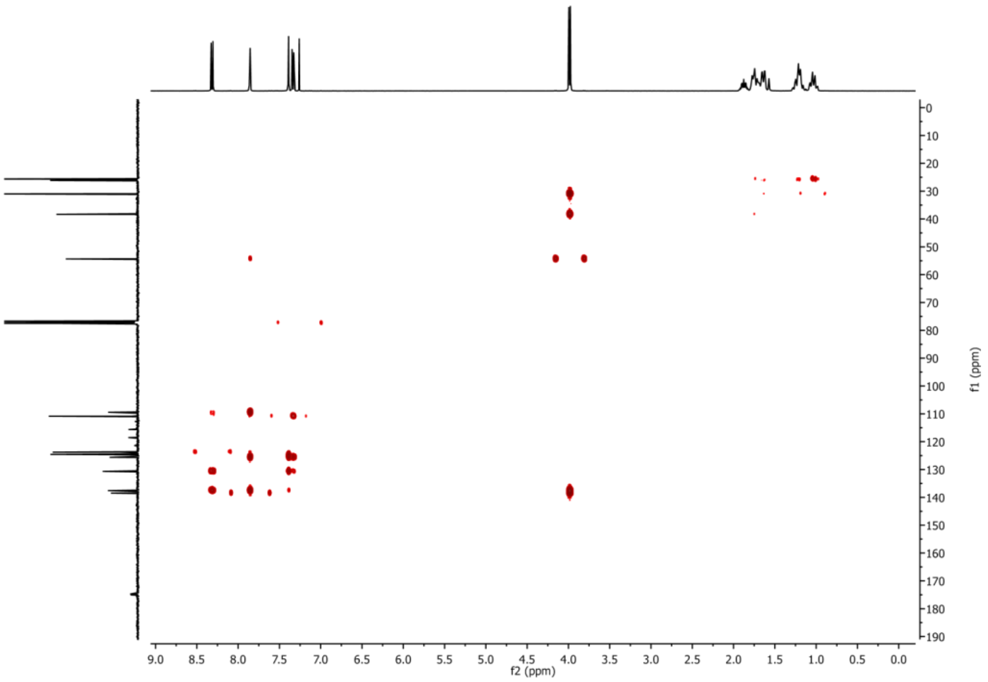

Figure S-30: HMBC of 17 (100.6 MHz, $CDCl_3$)

1-(7-Chloro-1(cyclohexylmethyl)-1*H*-indol-3-yl)-2,2,2-trifluorethan-1-one (18)

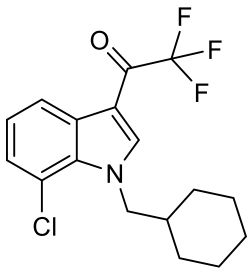

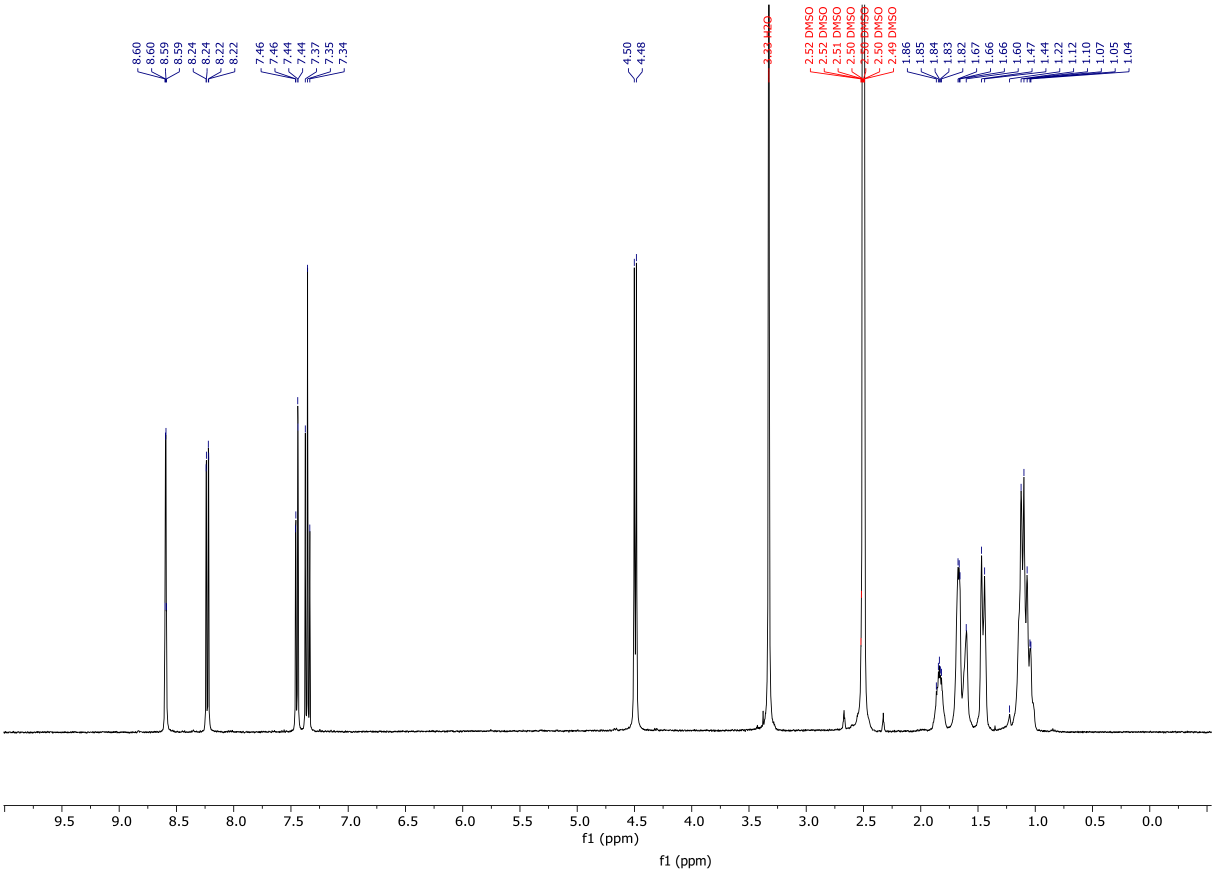

Figure S-31: ^{1}H-NMR of 18 (400 MHz, DMSO-d_6)

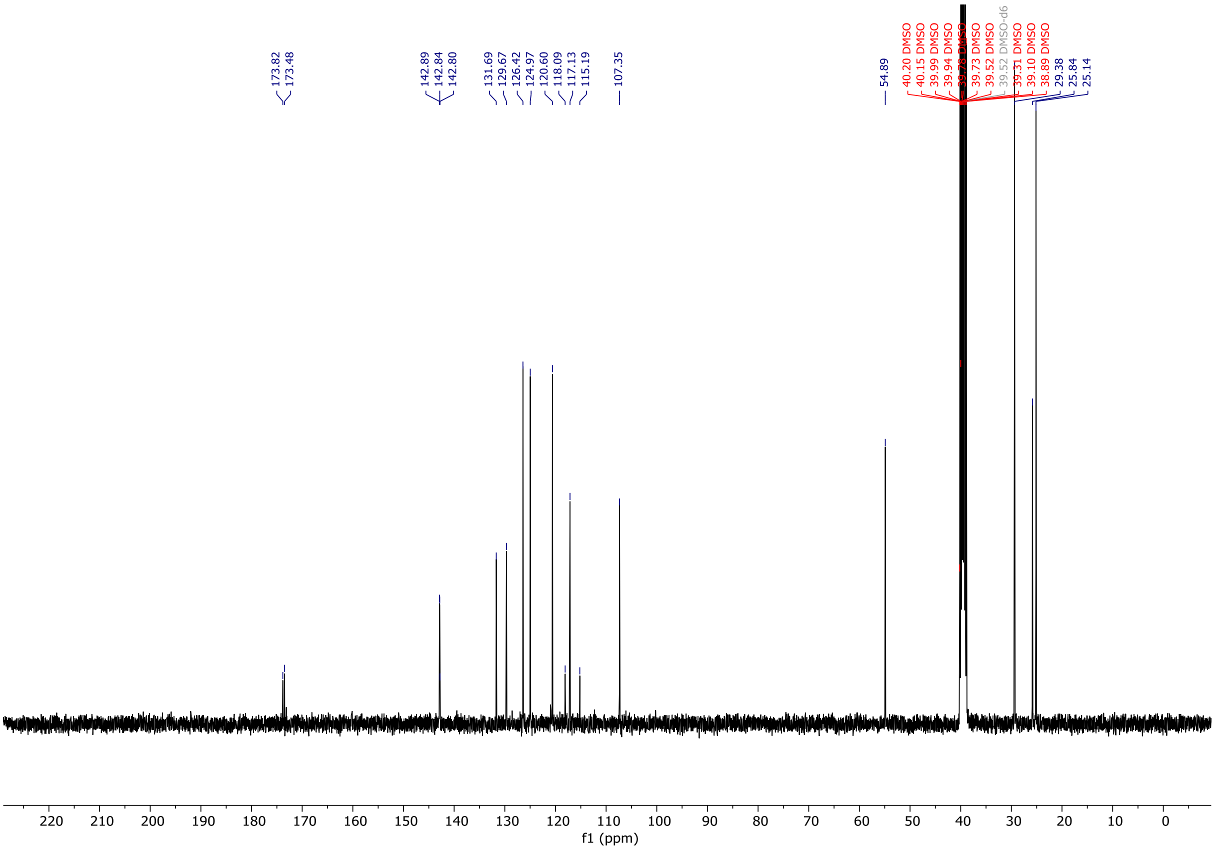

Figure S-32: ^{13}C-NMR of 18 (100.6 MHz, DMSO-d_6)

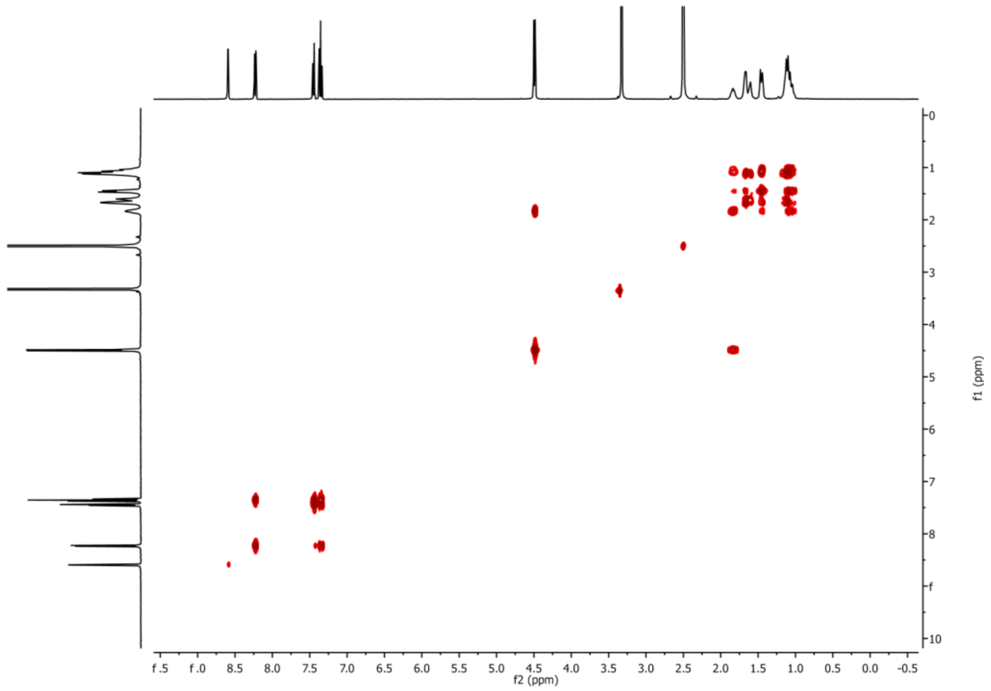

Figure S-33: COSY of 18 (400 MHz, DMSO-d_6)

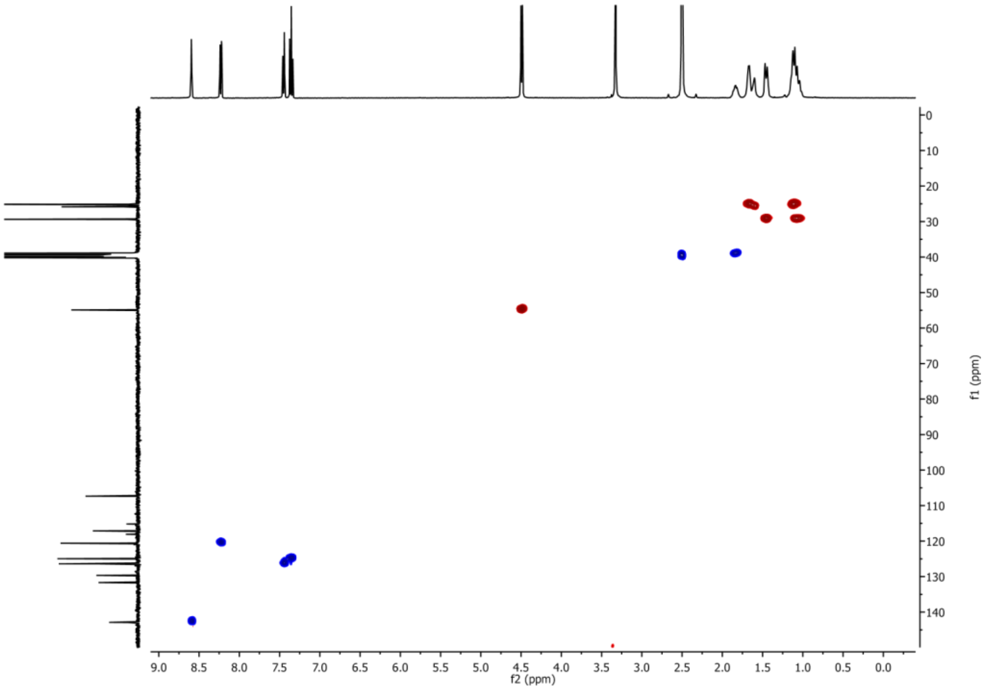

Figure S-34: HSQC of 18 (100.6 MHz, DMSO-d_6)

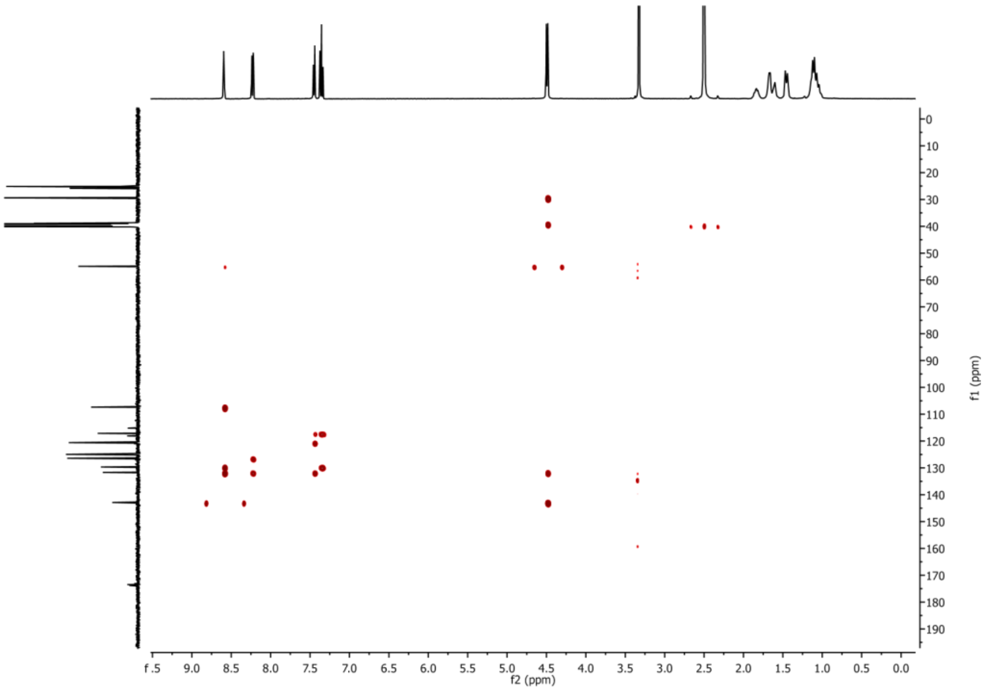

Figure S-35: HMBC of 18 (100.6 MHz, DMSO-d_6)

2-Chloro-1-(cyclohexylmethyl)-1*H*-indole-3-carboxylic acid (11)

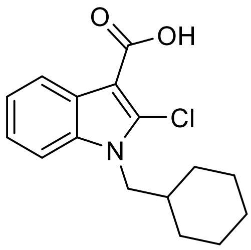

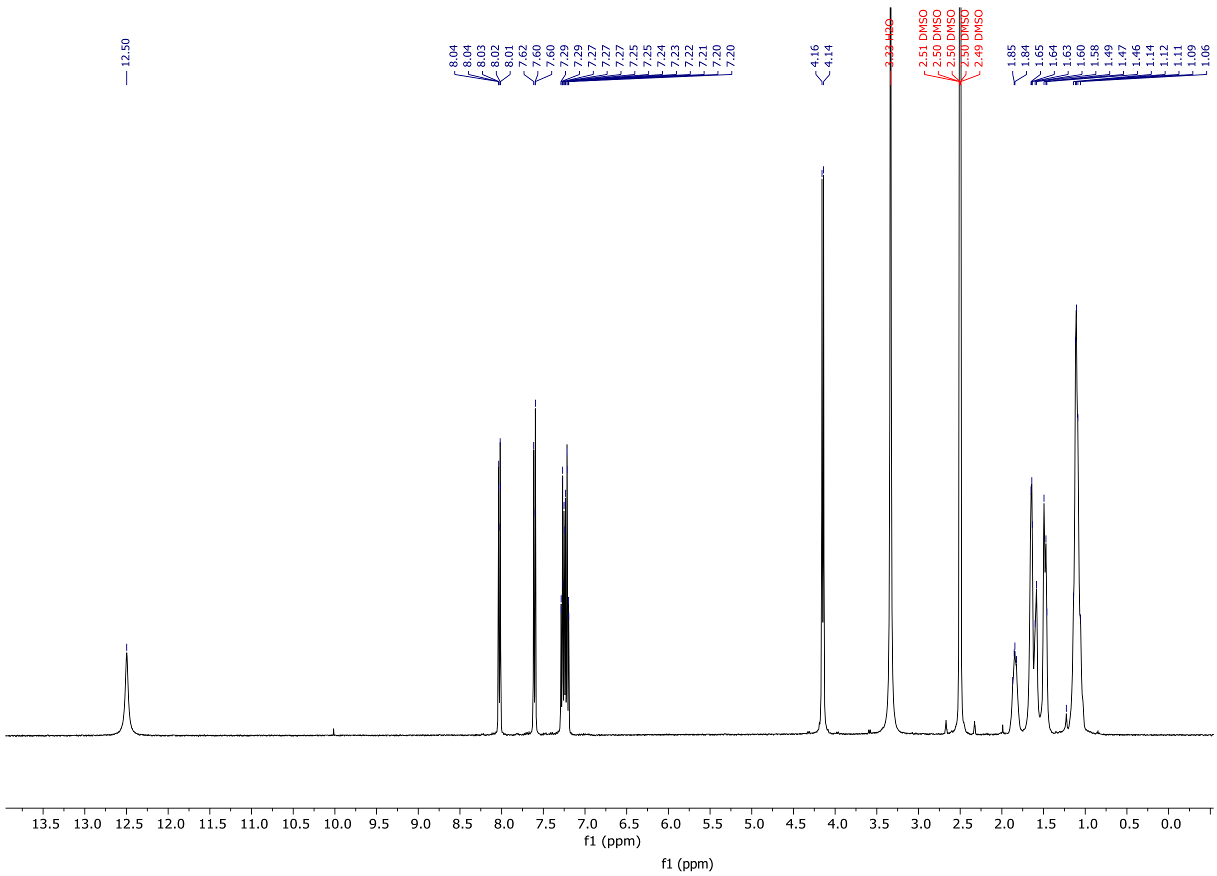

Figure S-36: ^{1}H-NMR of 11 (400 MHz, DMSO-*d*$_6$)

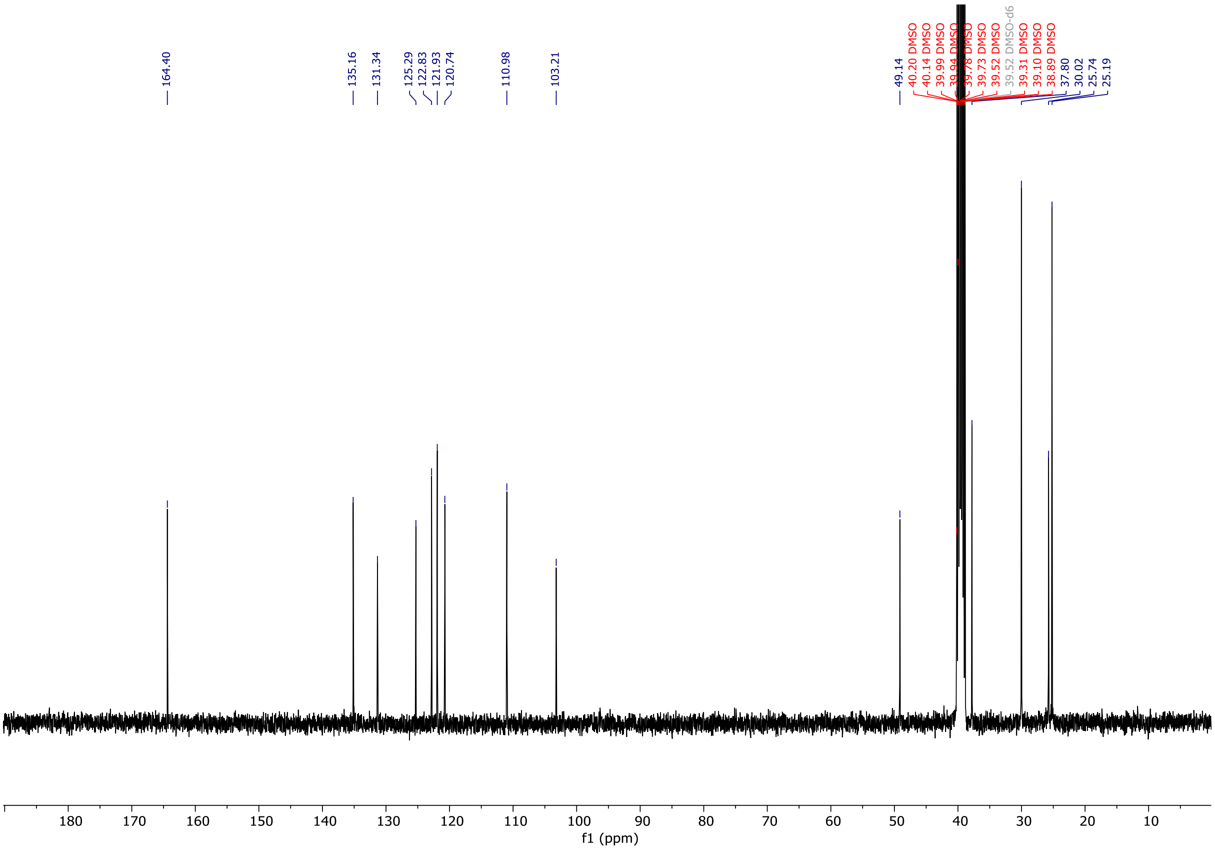

Figure S-37: ^{13}C-NMR of 11 (100.6 MHz, DMSO-d_6)

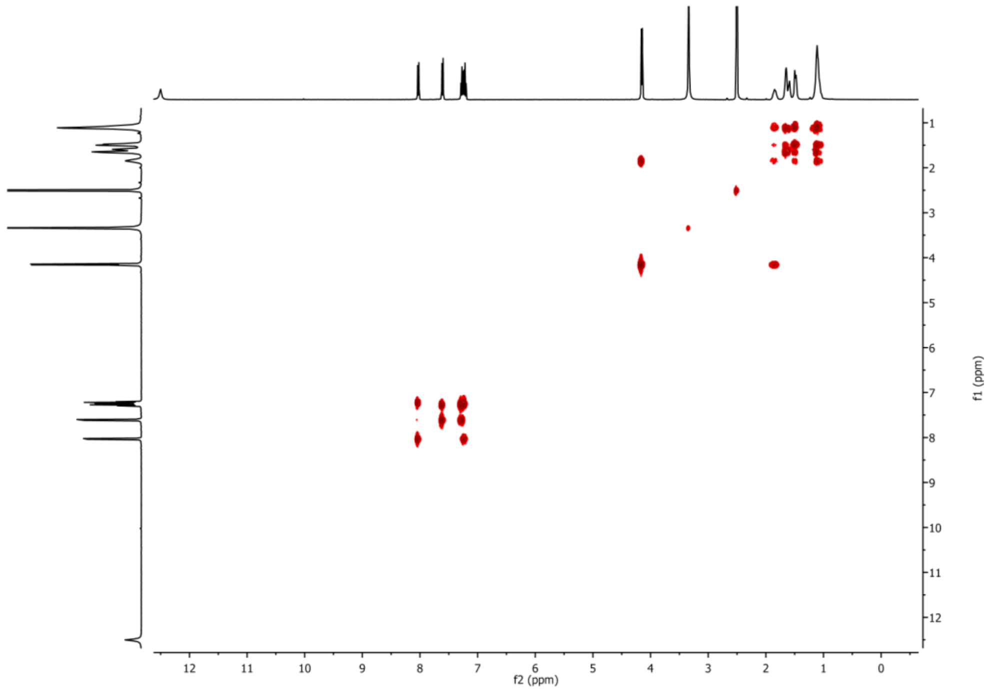

Figure S-38: COSY of 11 (400 MHz, DMSO-d_6)

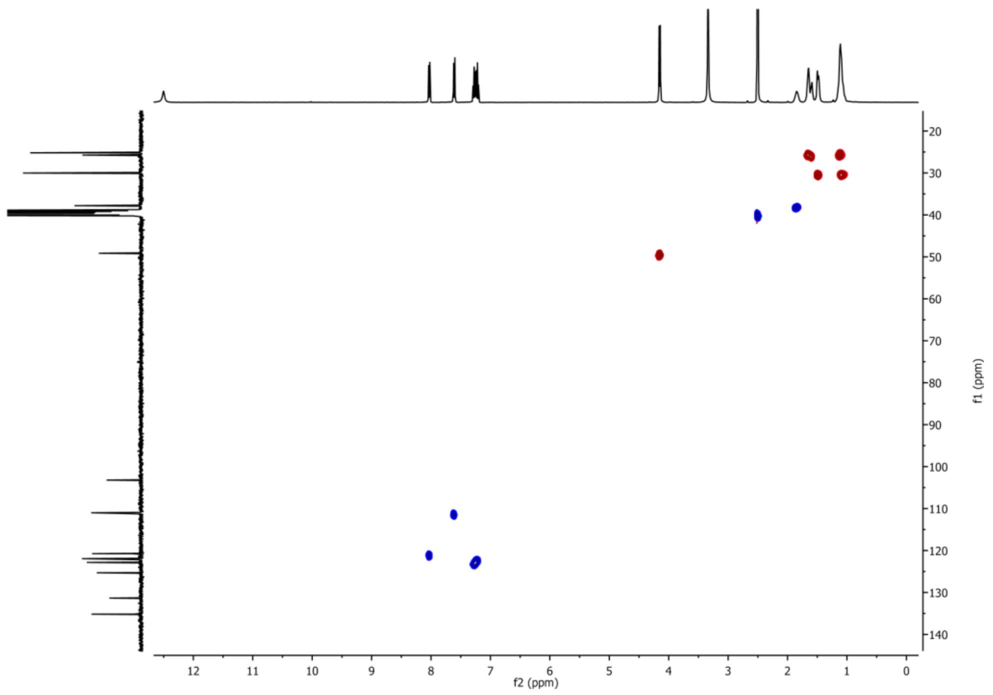

Figure S-39: HSQC of 11 (100.6 MHz, DMSO-d_6)

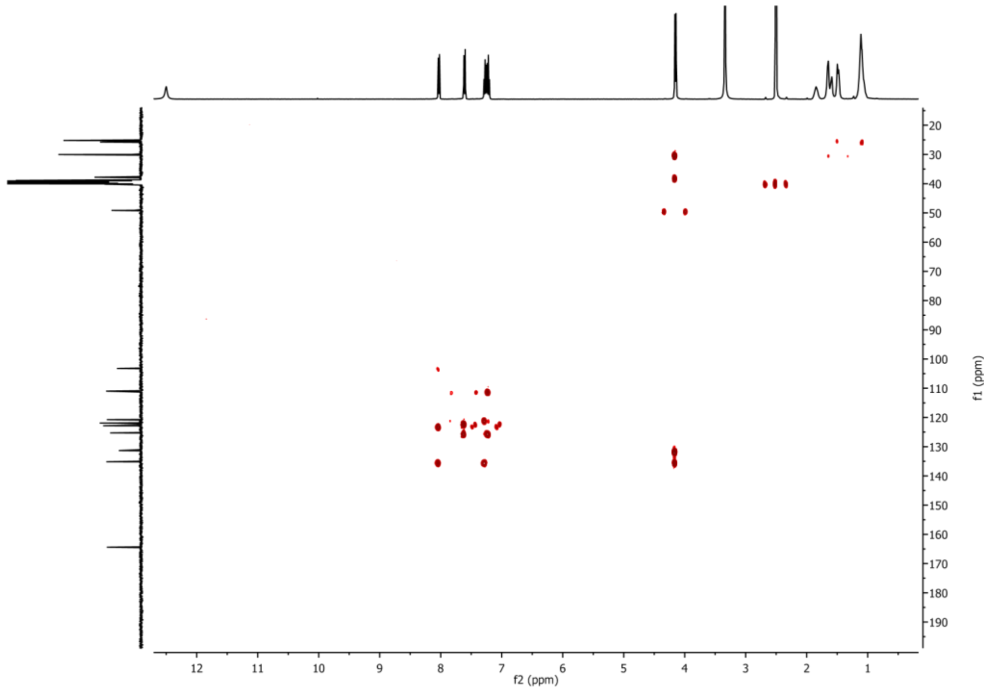

Figure S-40: HMBC of 11 (100.6 MHz, DMSO-d_6)

5-Chloro-1-(cyclohexylmethyl)-1*H*-indole-3-carboxylic acid (12)

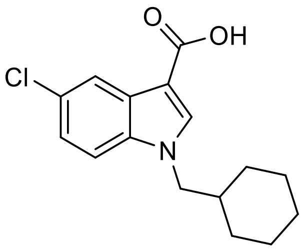

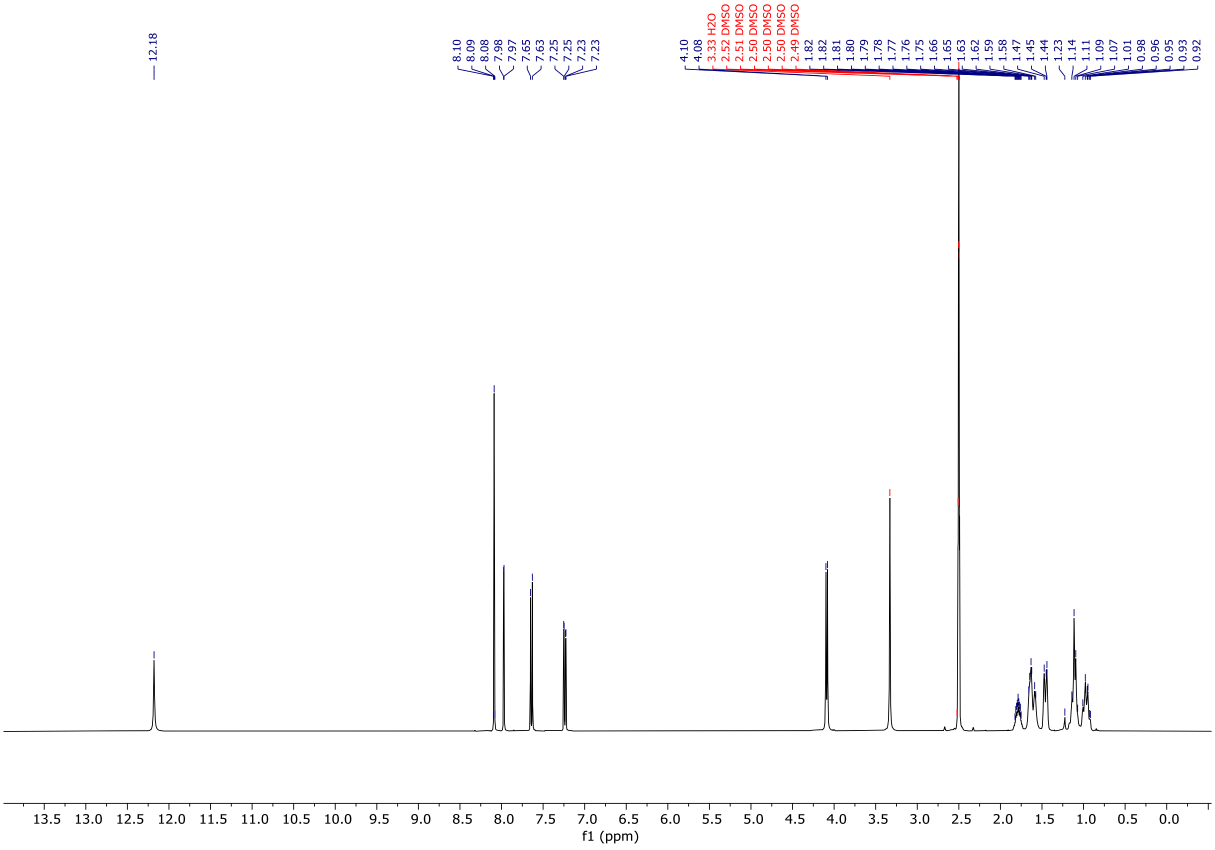

Figure S-41: ^{1}H-NMR of 12 (400 MHz, DMSO-d_6)

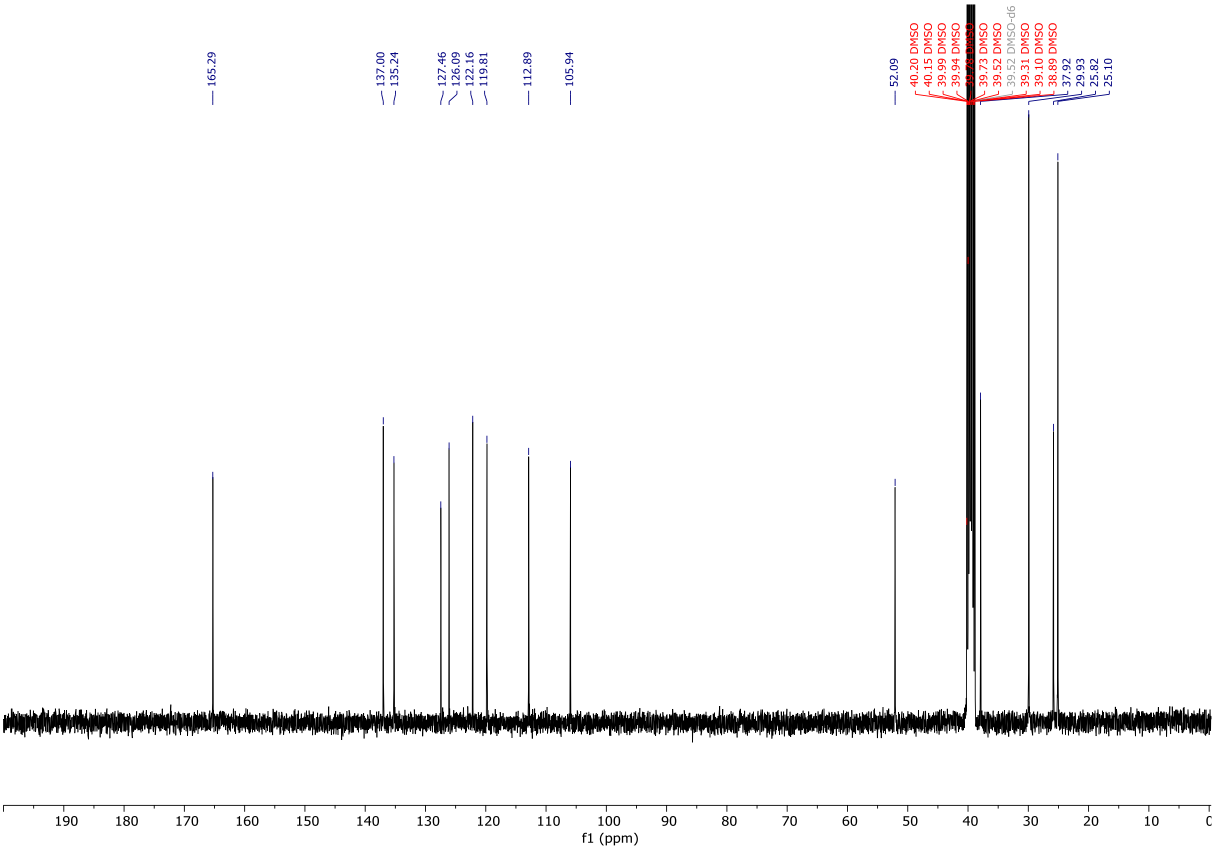

Figure S-42: ^{13}C-NMR of 12 (100.6 MHz, DMSO-d_6)

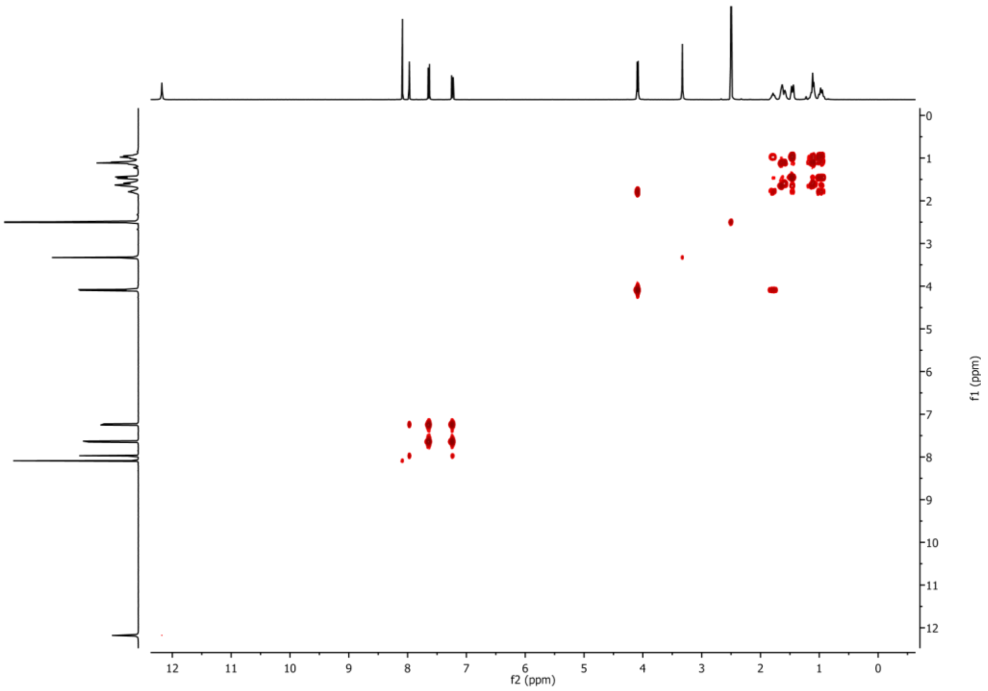

Figure S-43: COSY of 12 (400 MHz, DMSO-d_6)

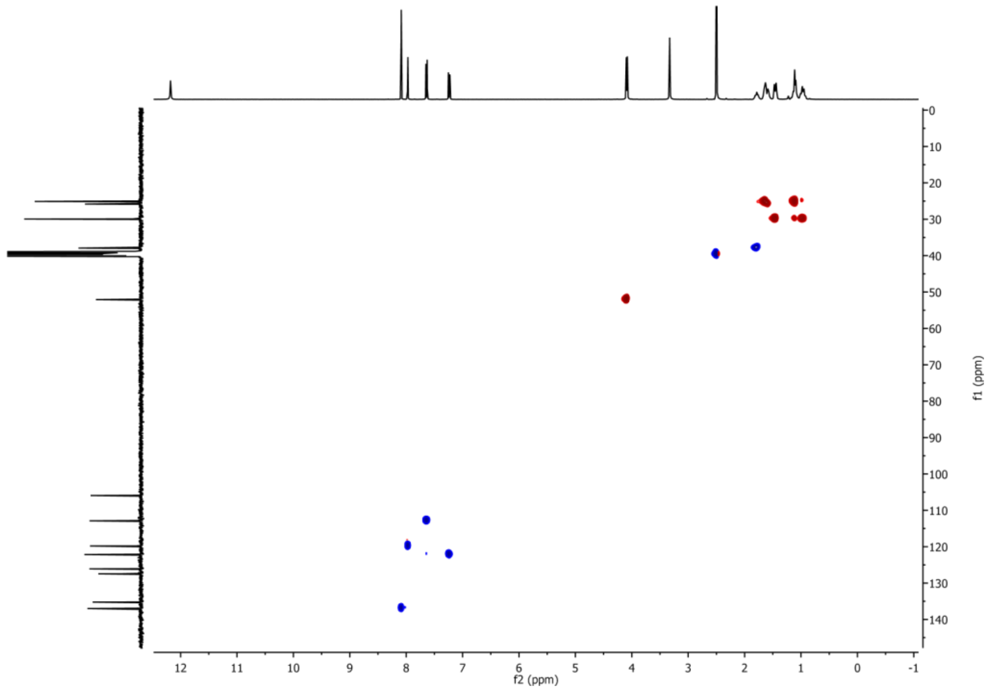

Figure S-44: HSQC of 12 (100.6 MHz, DMSO-d_6)

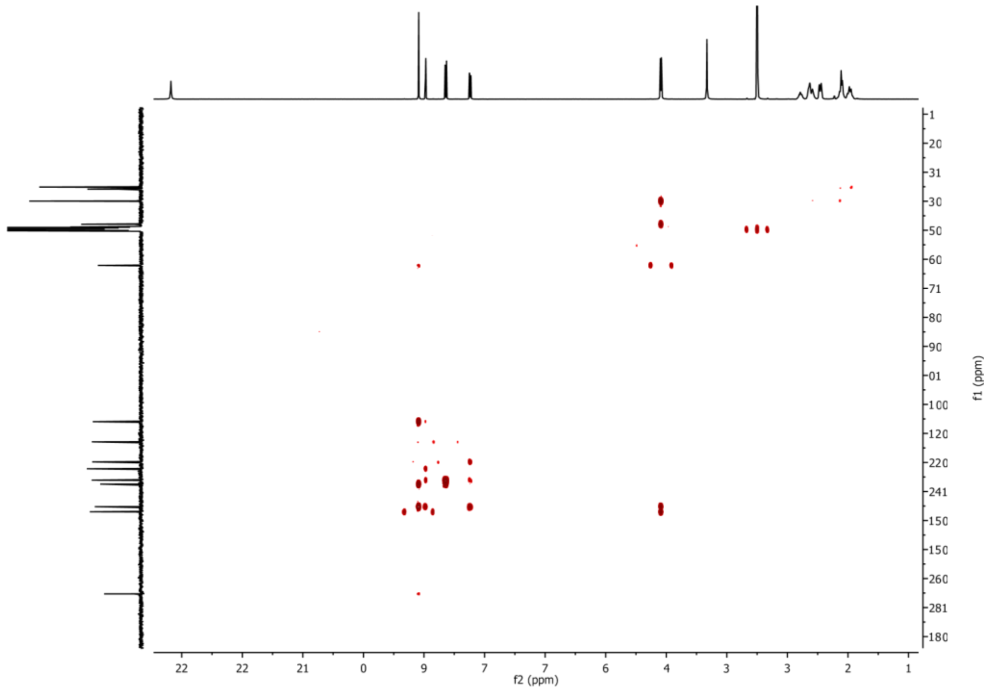

Figure S-45: HMBC of 12 (100.6 MHz, DMSO-d_6)

4-Chloro-1-(cyclohexylmethyl)-1*H*-indole-3-carboxylic acid (19)

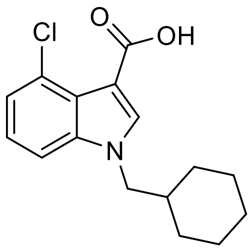

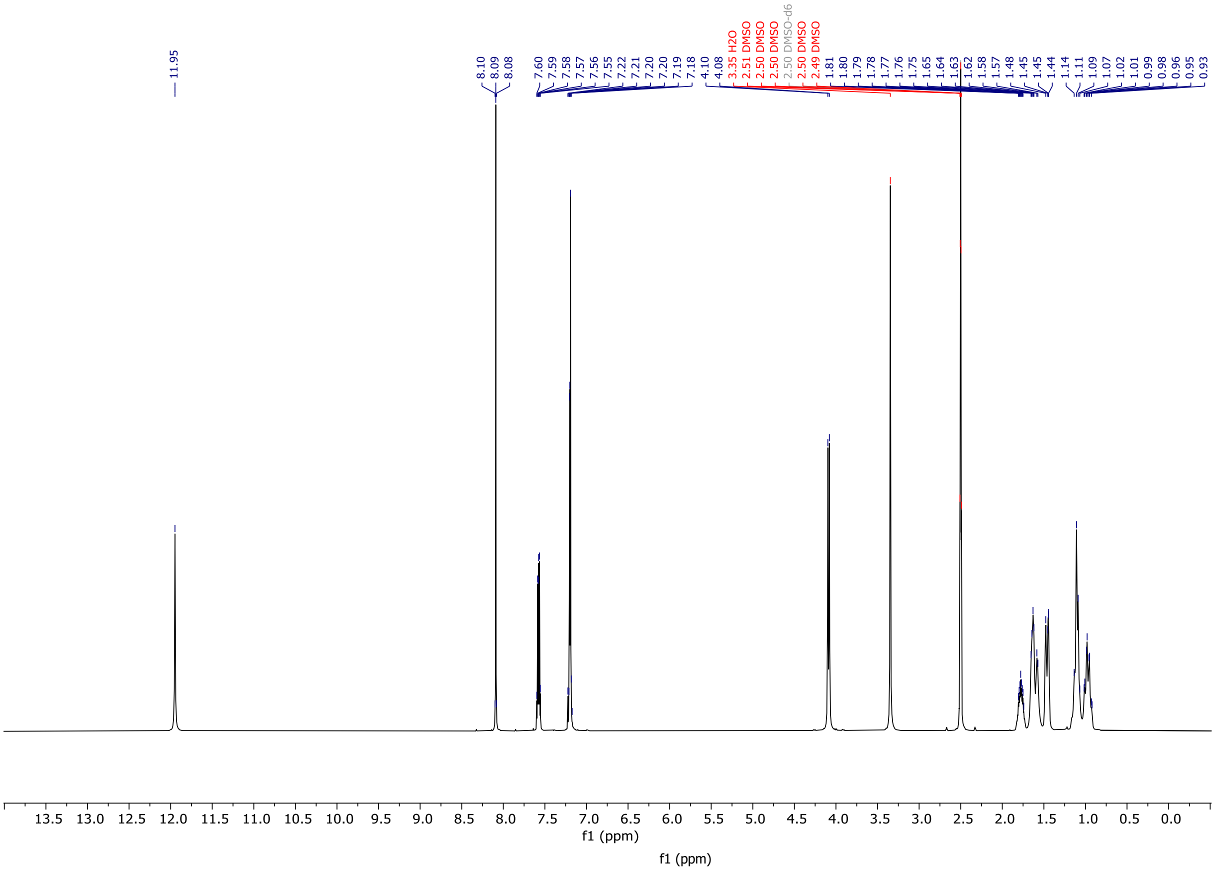

Figure S-46: ^{1}H-NMR of 19 (400 MHz, DMSO-d_6)

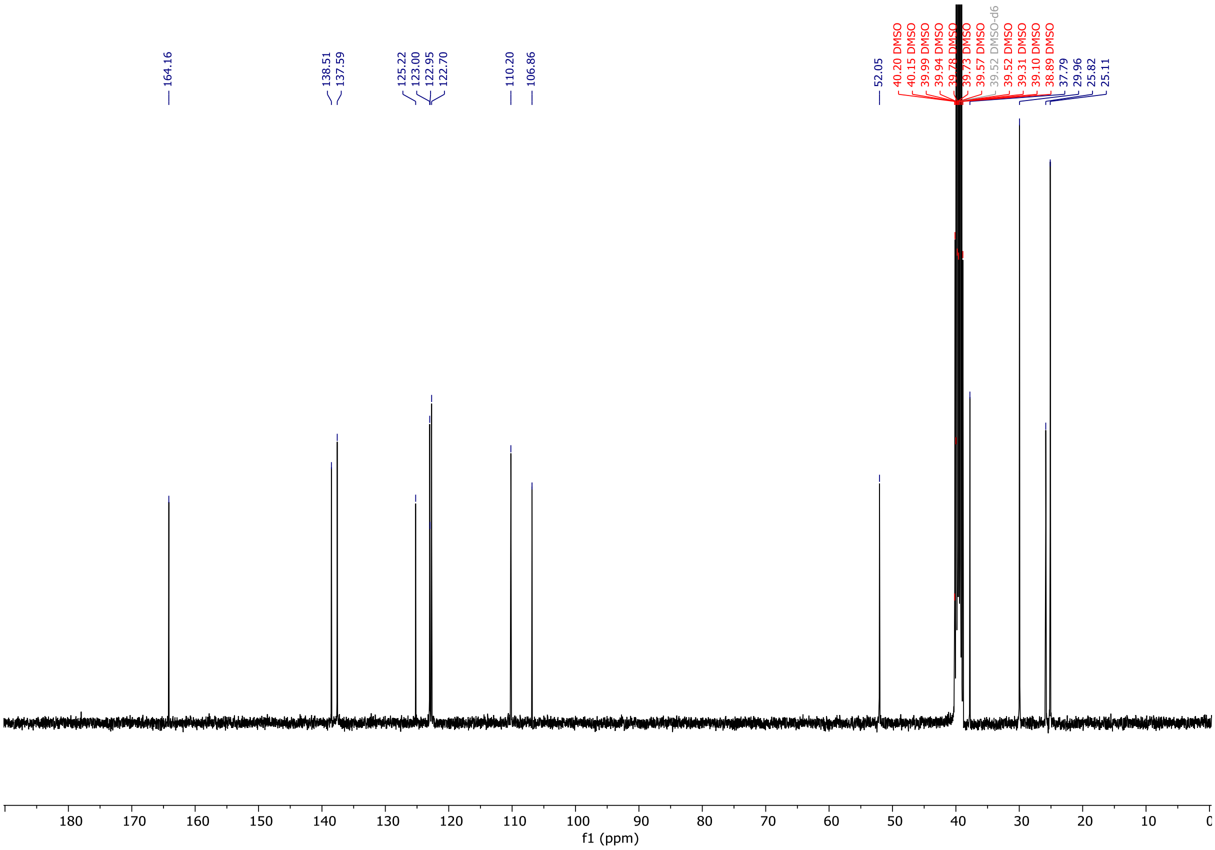

Figure S-47: ^{13}C-NMR of 19 (100.6 MHz, DMSO-d_6)

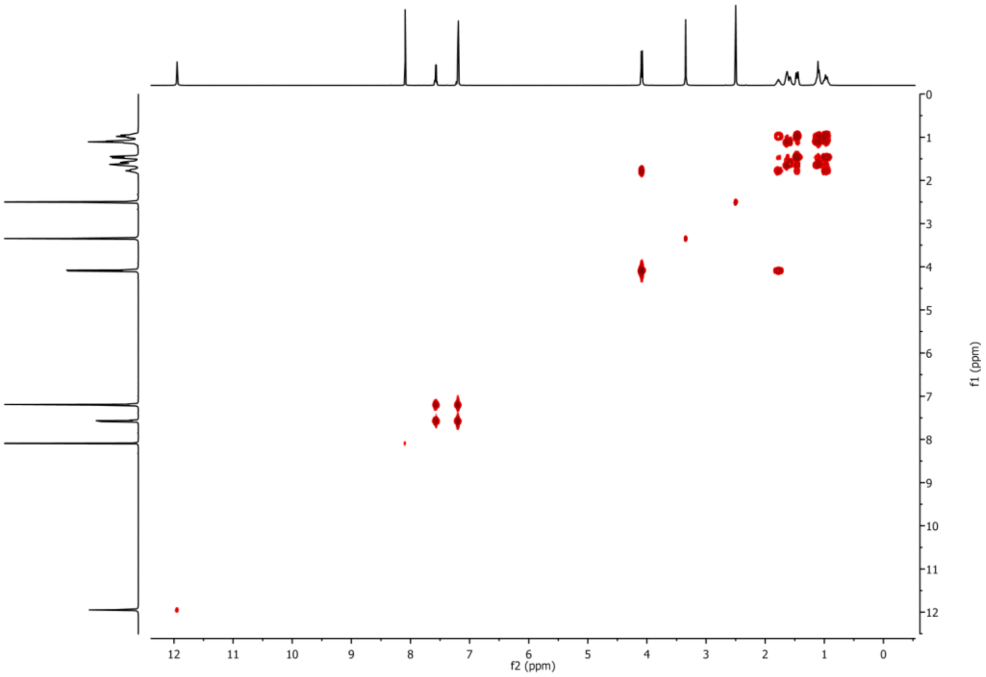

Figure S-48: COSY of 19 (400 MHz, DMSO-d_6)

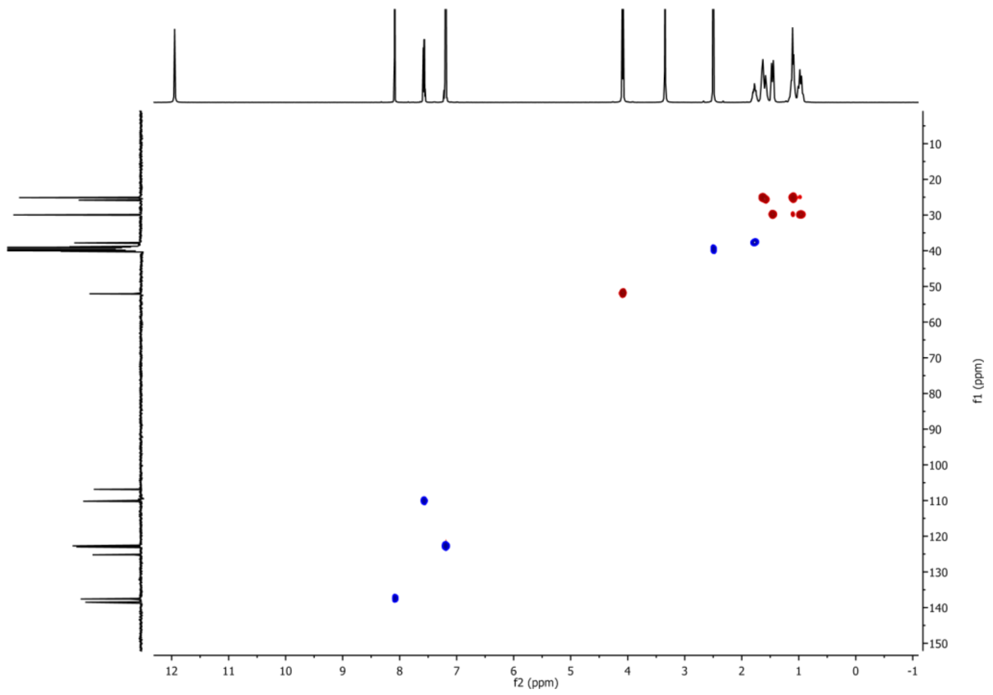

Figure S-49: HSQC of 19 (100.6 MHz, DMSO-d_6)

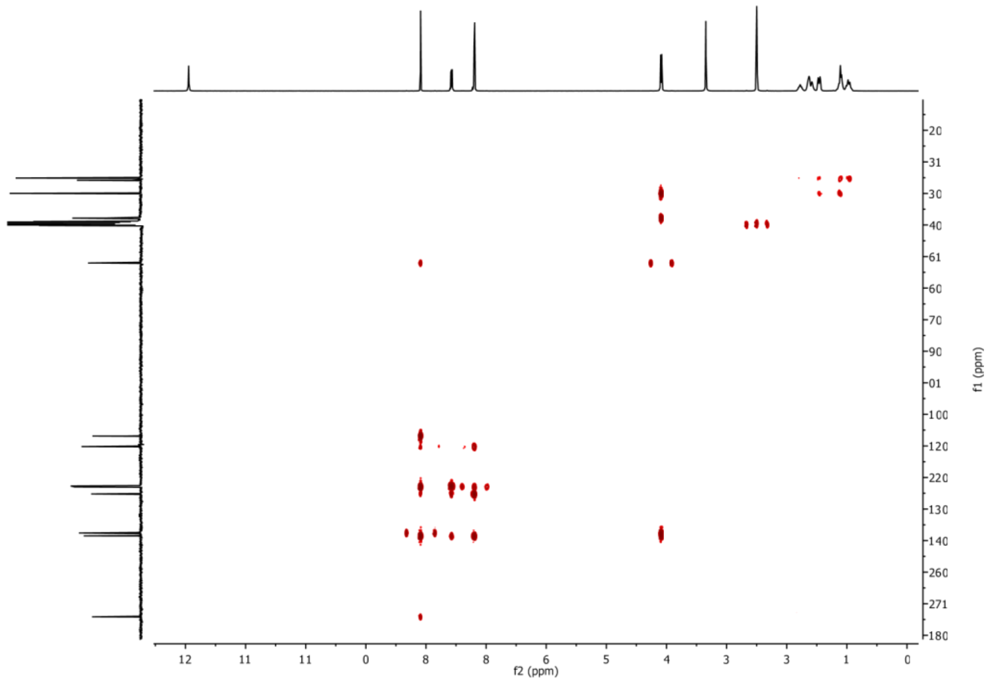

Figure S-50: HMBC of 19 (100.6 MHz, DMSO-d_6)

6-Chloro-1-(cyclohexylmethyl)-1*H*-indole-3-carboxylic acid (20)

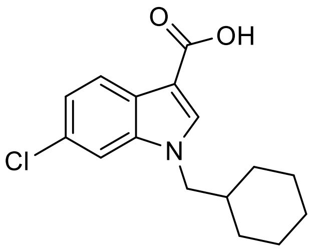

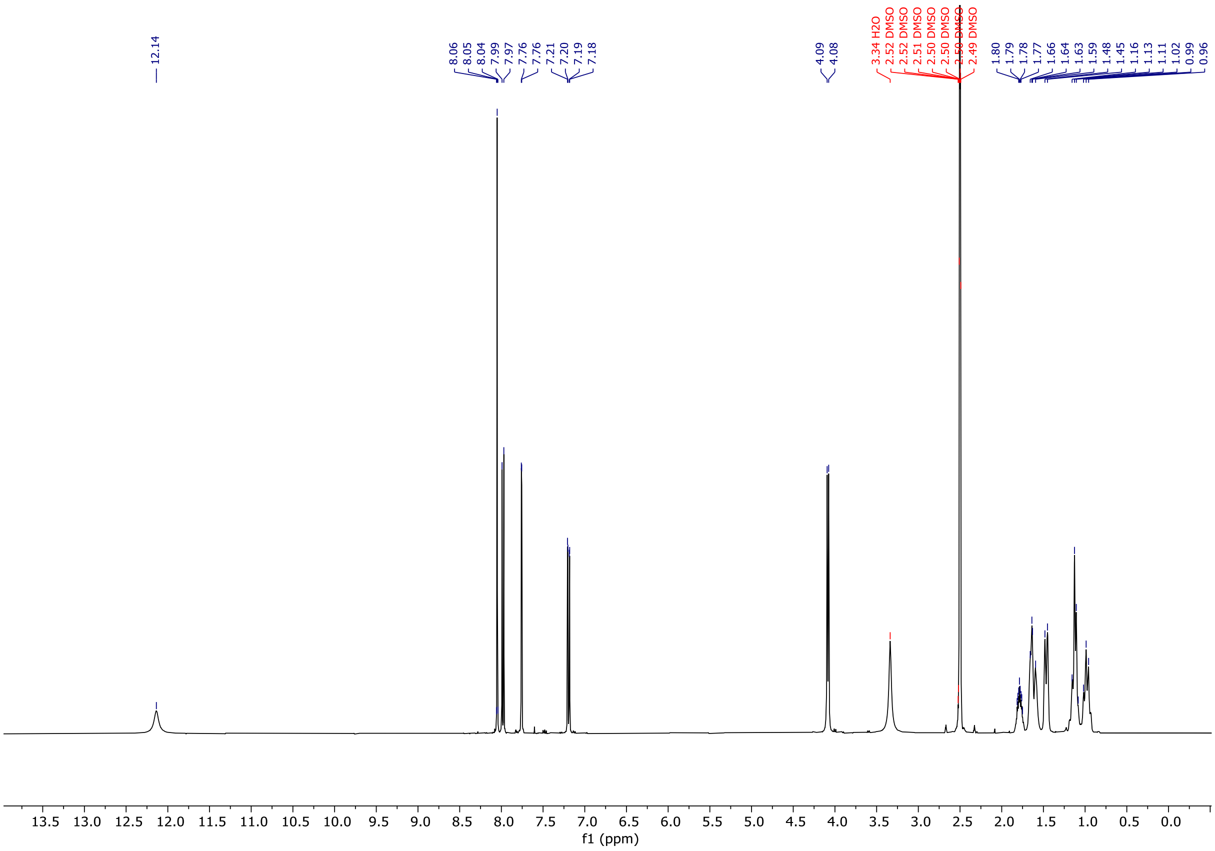

Figure S-51: ^{1}H-NMR of 20 (400 MHz, DMSO-d_6)

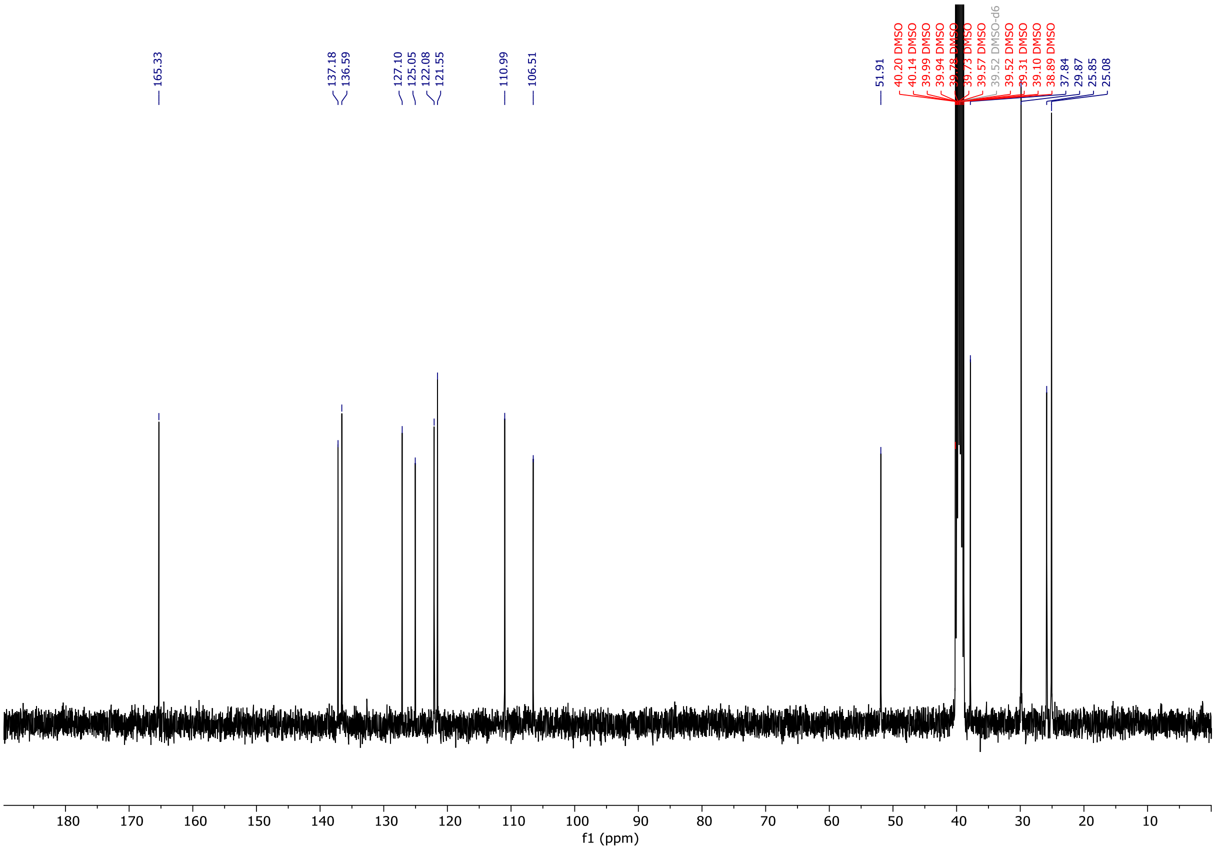

Figure S-52: ^{13}C-NMR of 20 (100.6 MHz, DMSO-d_6)

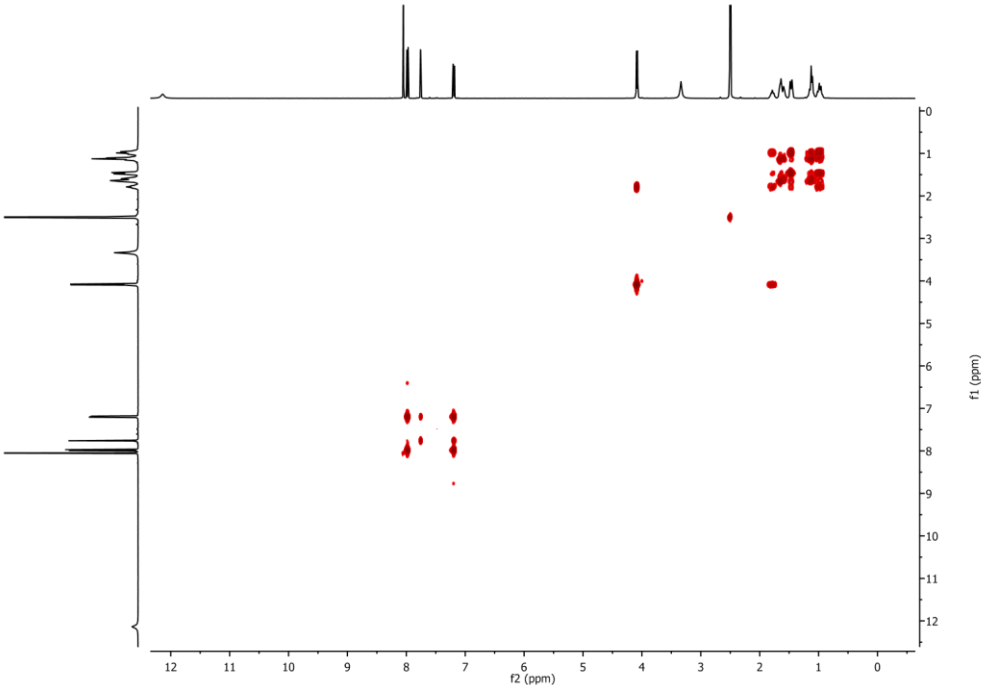

Figure S-53: COSY of 20 (400 MHz, DMSO-d_6)

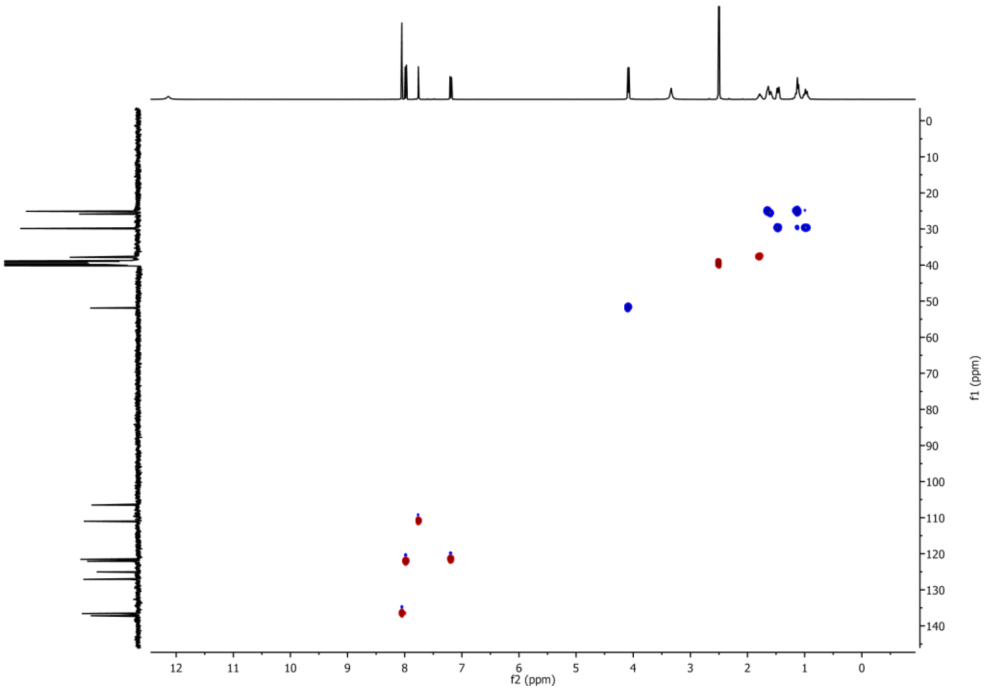

Figure S-54: HSQC of 20 (100.6 MHz, DMSO-*d*$_6$)

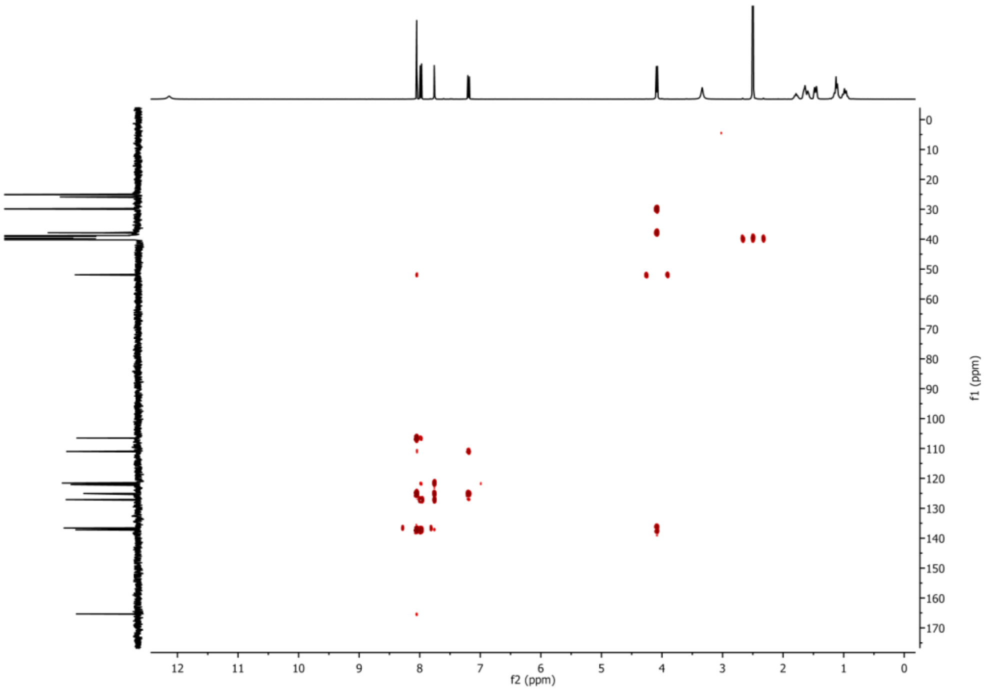

Figure S-55: HMBC of 20 (100.6 MHz, DMSO-*d*$_6$)

7-Chloro-1-(cyclohexylmethyl)-1*H*-indole-3-carboxylic acid (21)

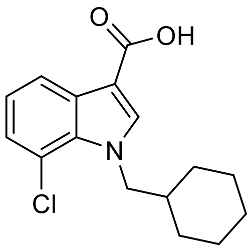

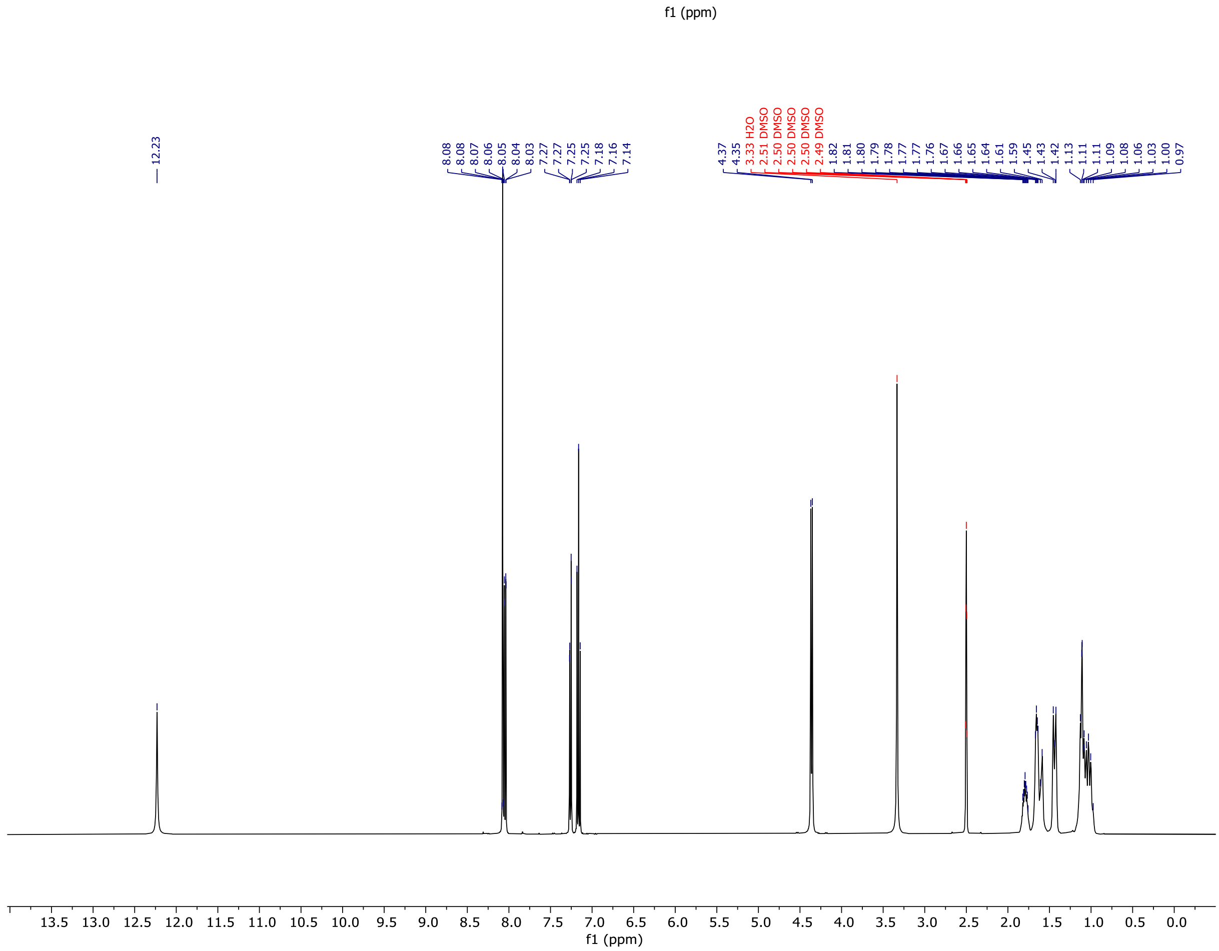

Figure S-56: ^{1}H-NMR of 21 (400 MHz, DMSO-d_6)

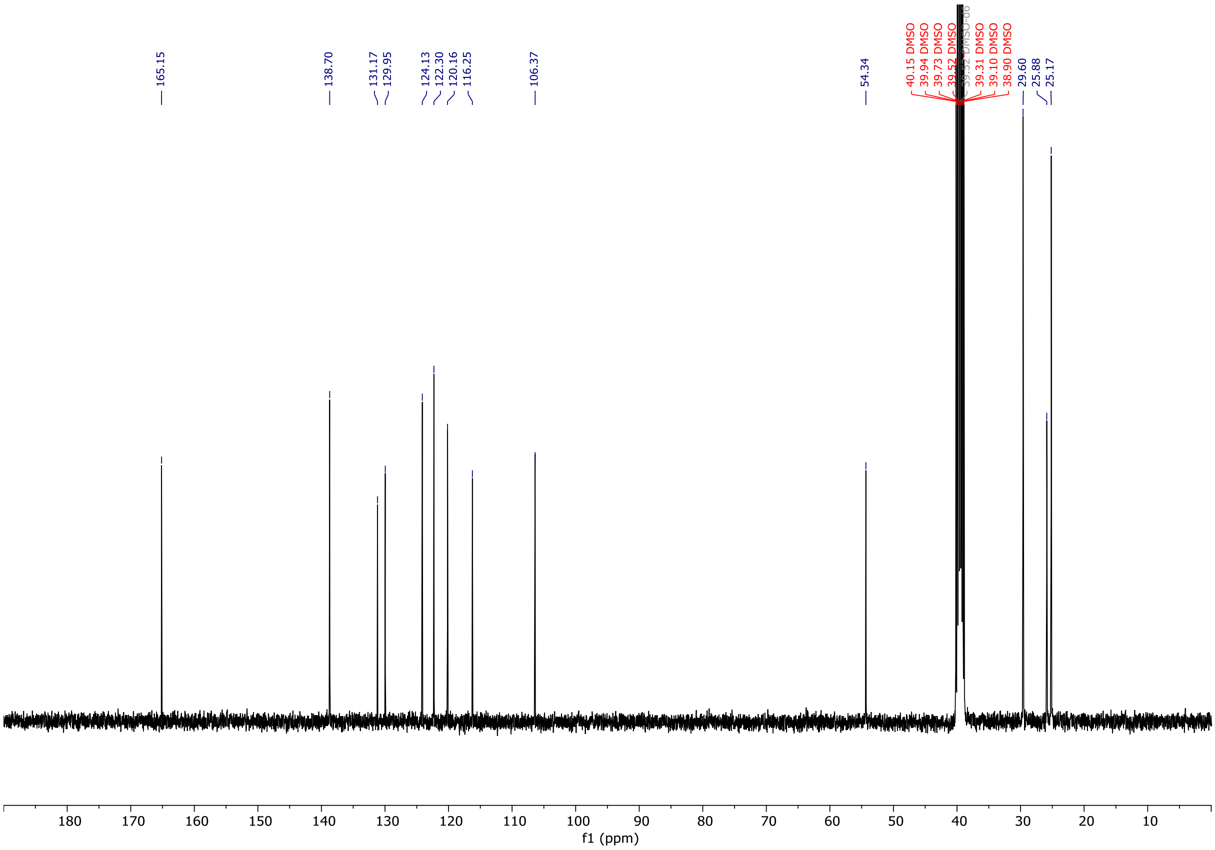

Figure S-57: ^{13}C-NMR of 21 (100.6 MHz, DMSO-*d*$_6$)

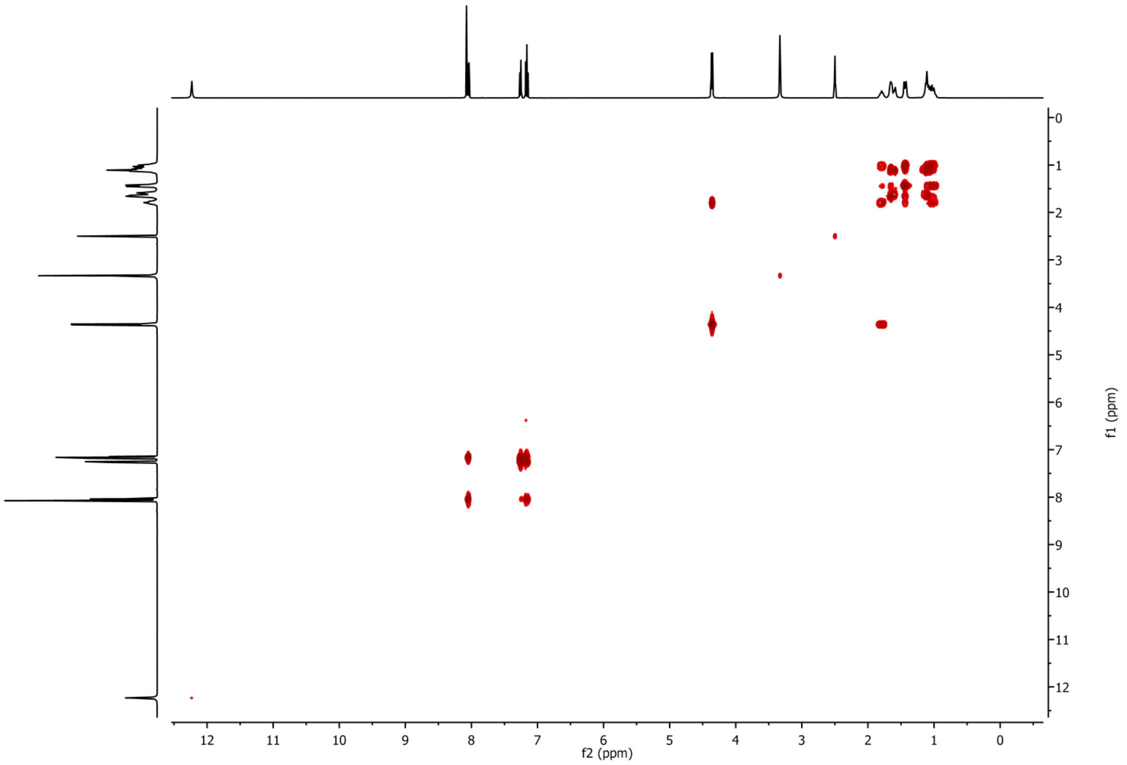

Figure S-58: COSY of 21 (400 MHz, DMSO-*d*$_6$)

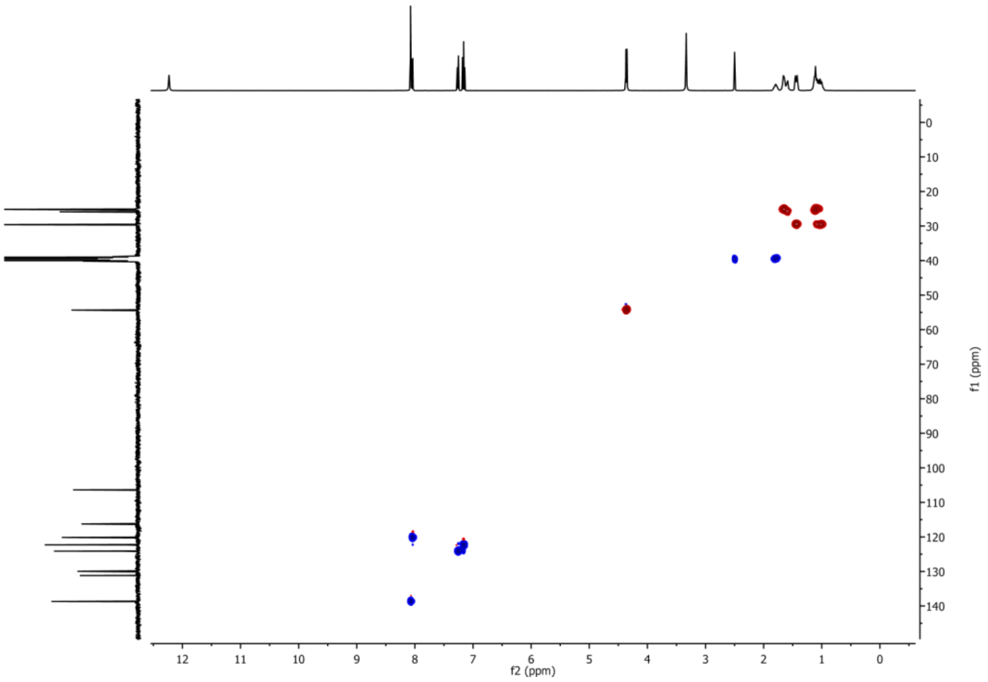

Figure S-59: HSQC of 21 (100.6 MHz, DMSO-d_6)

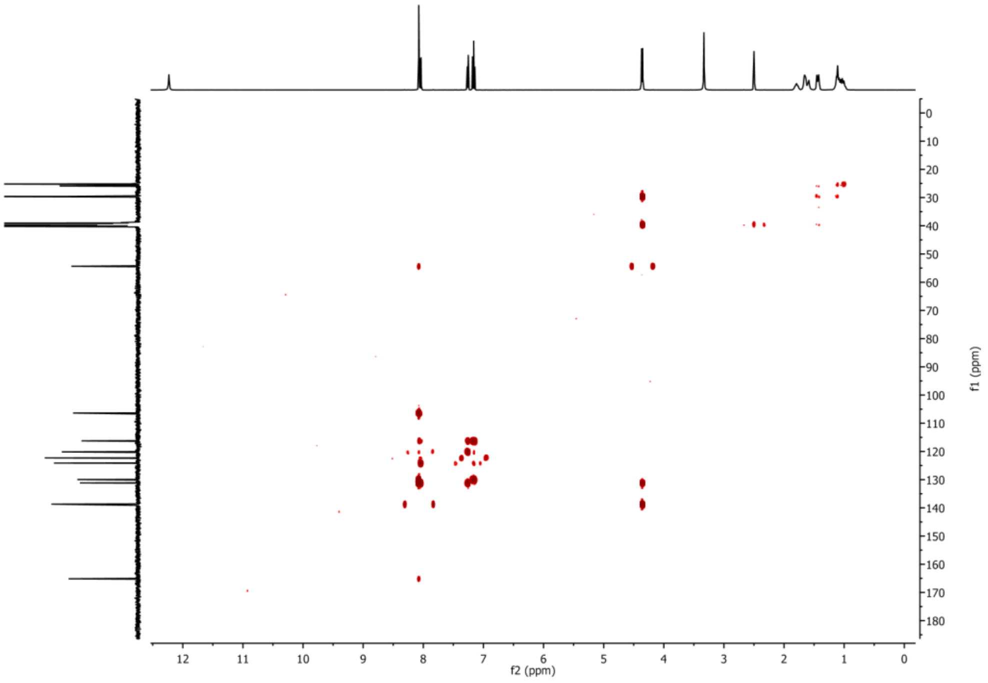

Figure S-60: HMBC of 21 (100.6 MHz, DMSO-d_6)

Methyl (*S*)-2-(2-chloro-1-(cyclohexylmethyl)-1*H*-indole-3-carboxamido)-3,3-dimethylbutanoate (2-Chloro-MDMB-CHMICA) (2)

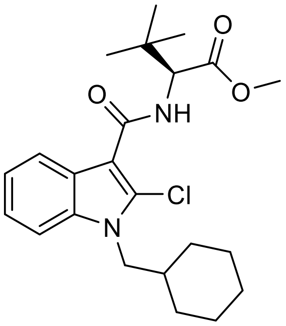

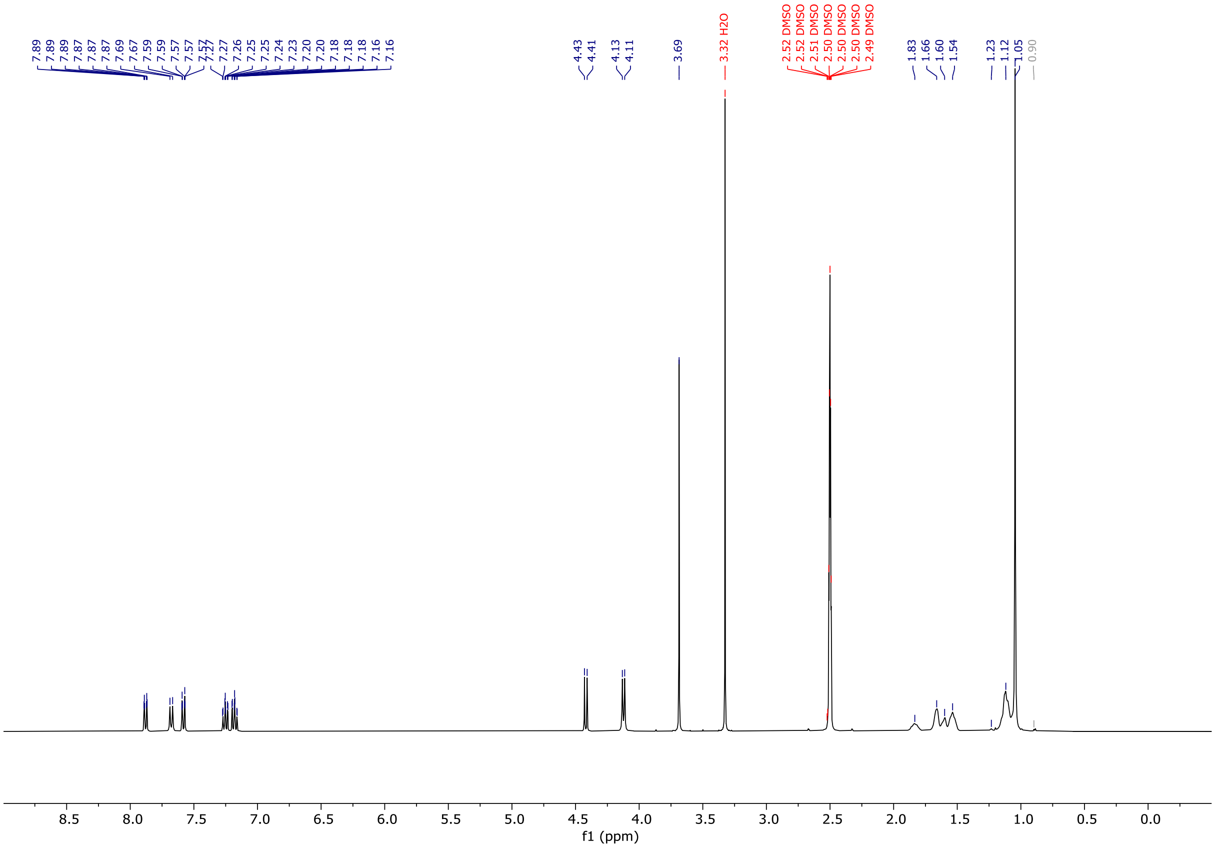

Figure S-61: ^{1}H-NMR of 2 (400 MHz, DMSO-d_6)

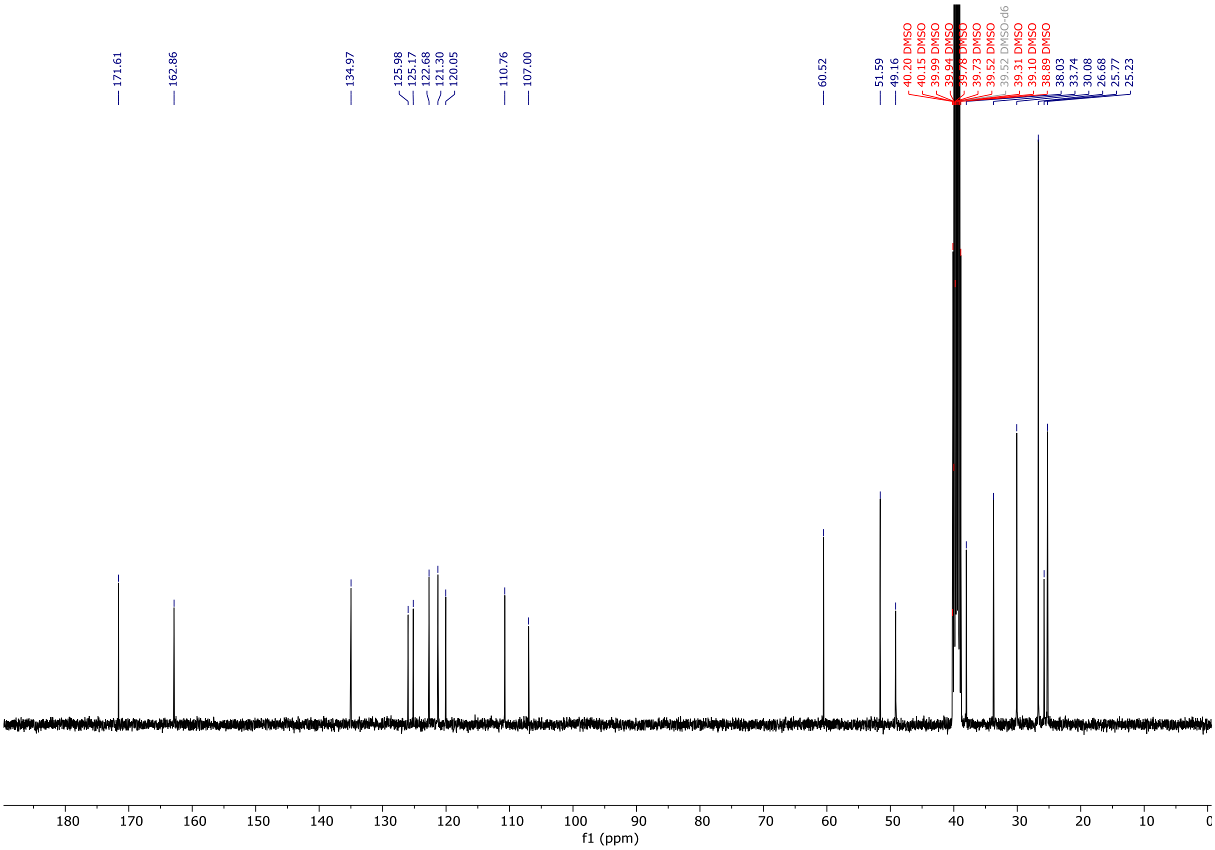

Figure S-62: ^{13}C-NMR of 2 (100.6 MHz, DMSO-*d6*)

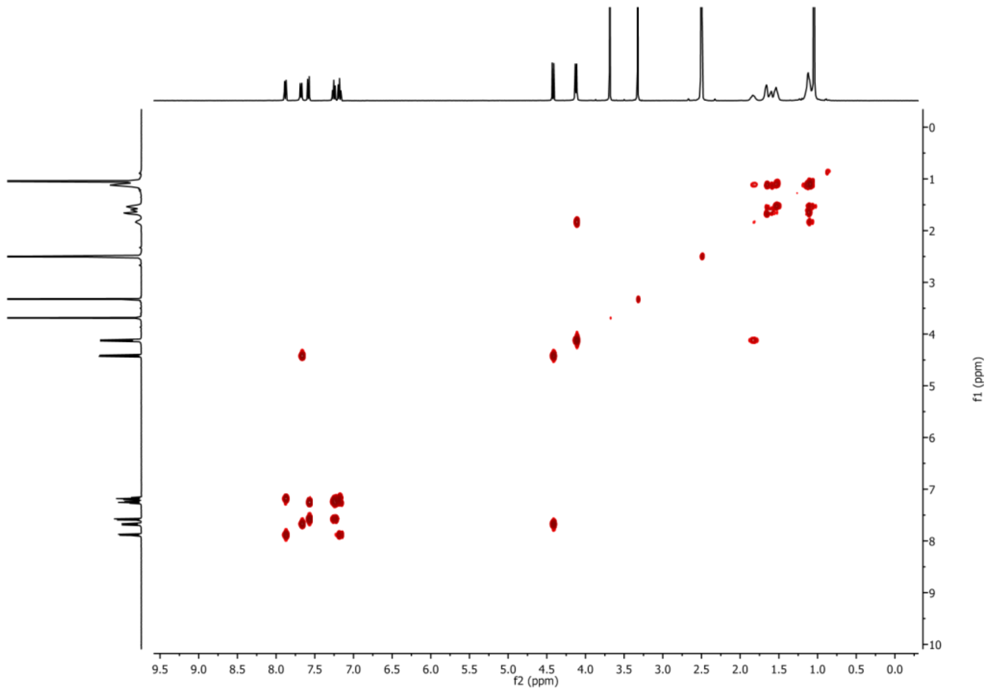

Figure S-63: COSY of 2 (400 MHz, DMSO-d_6)

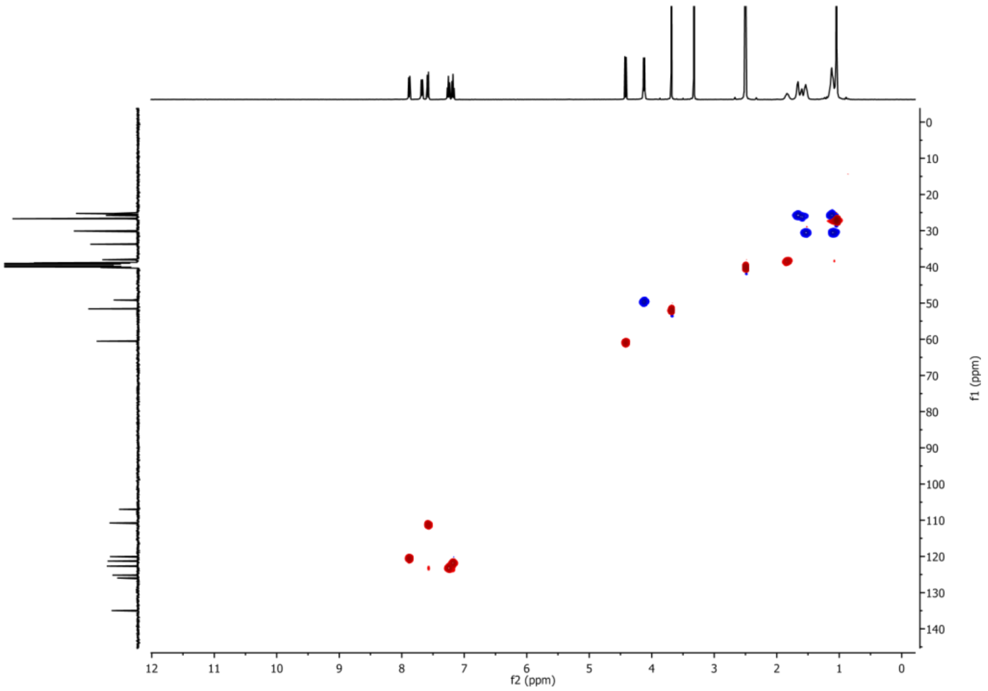

Figure S-64: HSQC of 2 (100.6 MHz, DMSO-d_6)

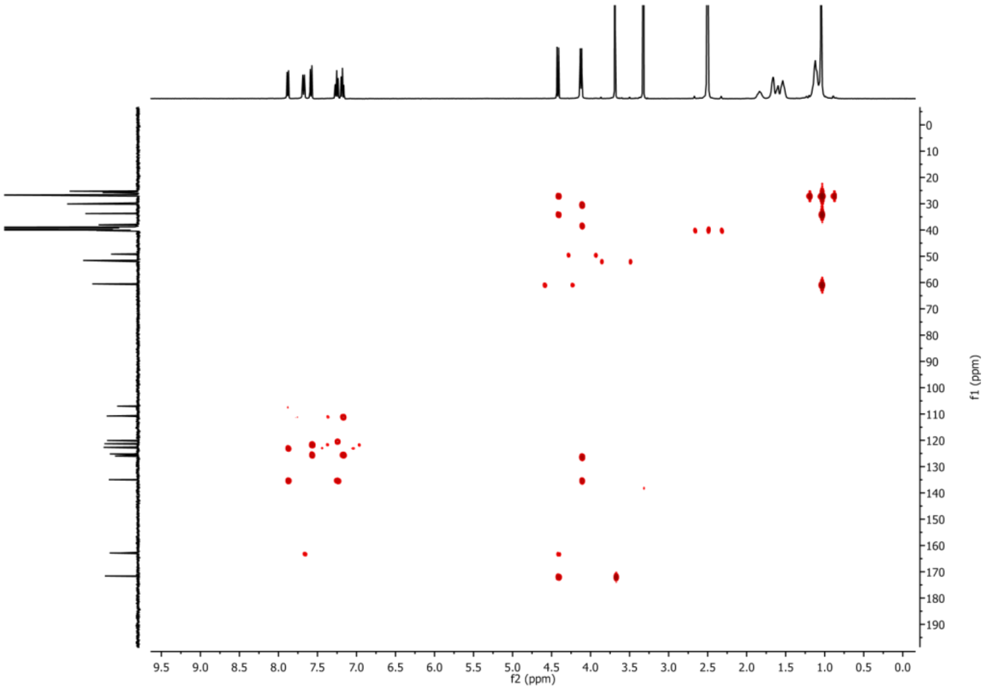

Figure S-65: HMBC of 2 (100.6 MHz, DMSO-d_6)

Methyl (*S*)-2-(4-chloro-1-(cyclohexylmethyl)-1*H*-indole-3-carboxamido)-3,3-dimethylbutanoate (4-Chloro-MDMB-CHMICA) (3)

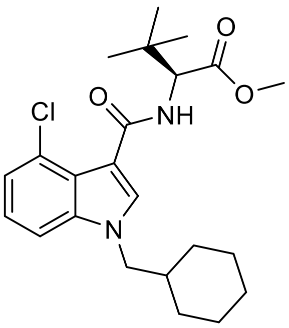

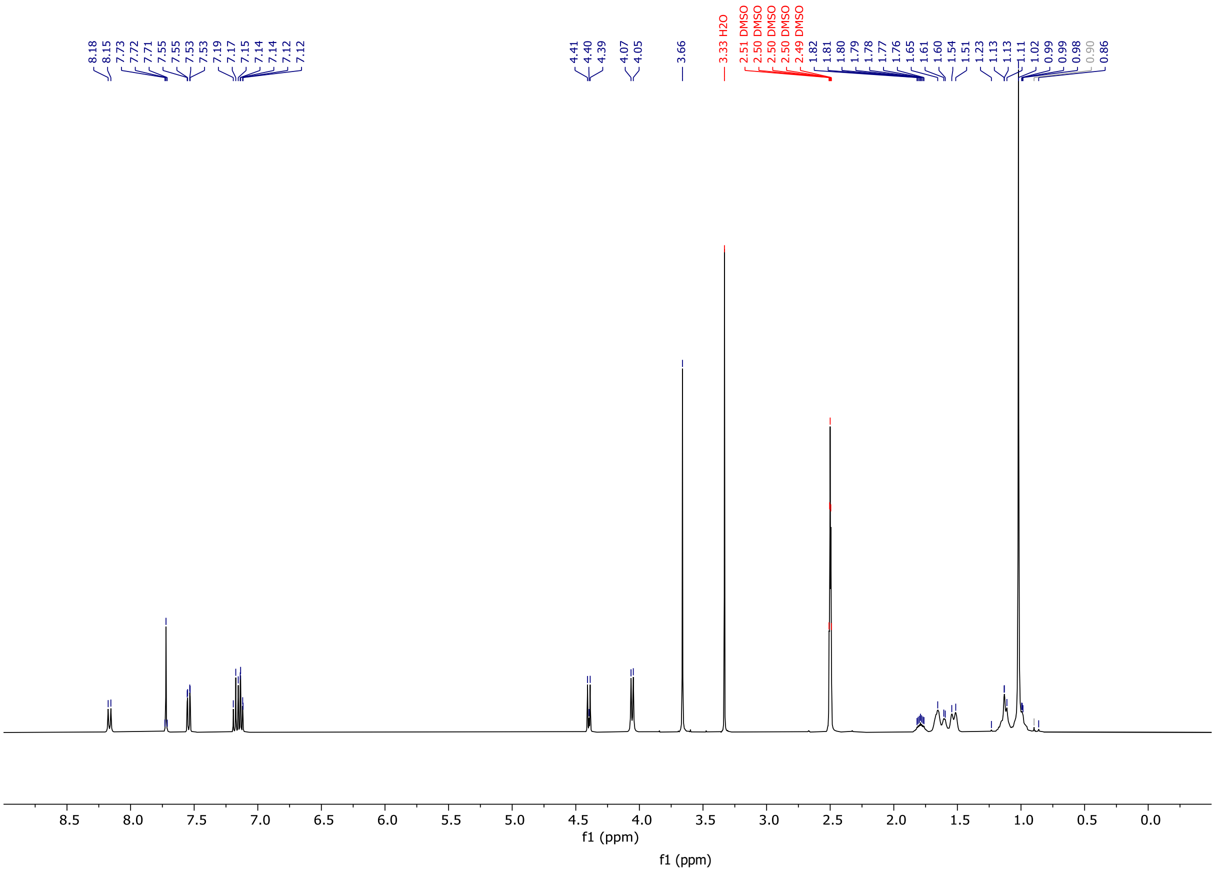

Figure S-66: ^{1}H-NMR of 3 (400 MHz, DMSO-d_6)

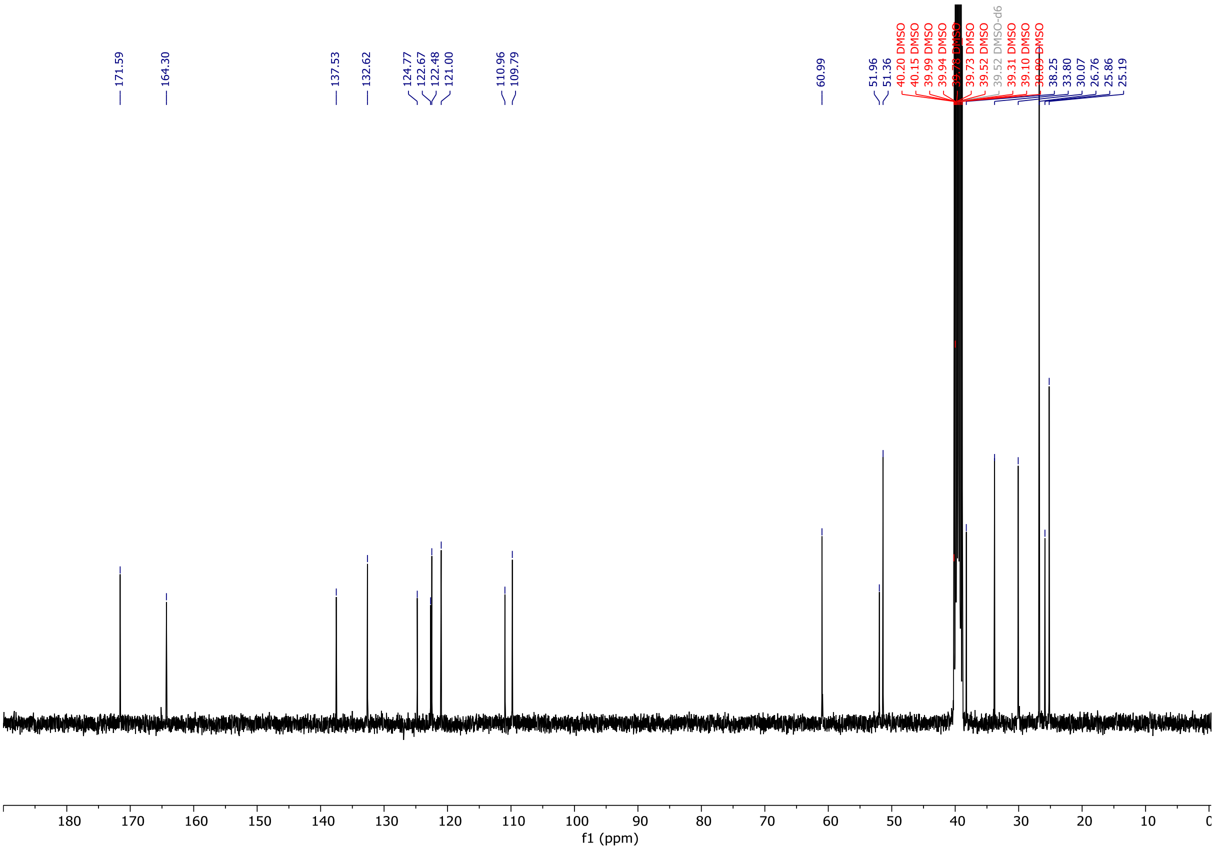

Figure S-67: ^{13}C-NMR of 3 (100.6 MHz, DMSO-d_6)

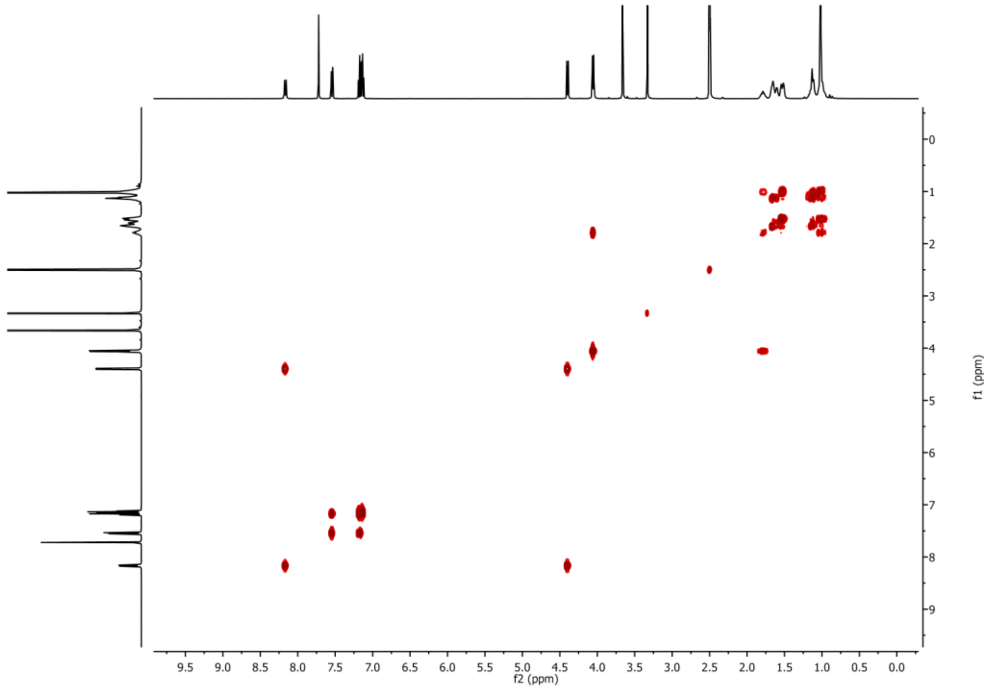

Figure S-68: COSY of 3 (400 MHz, DMSO-d_6)

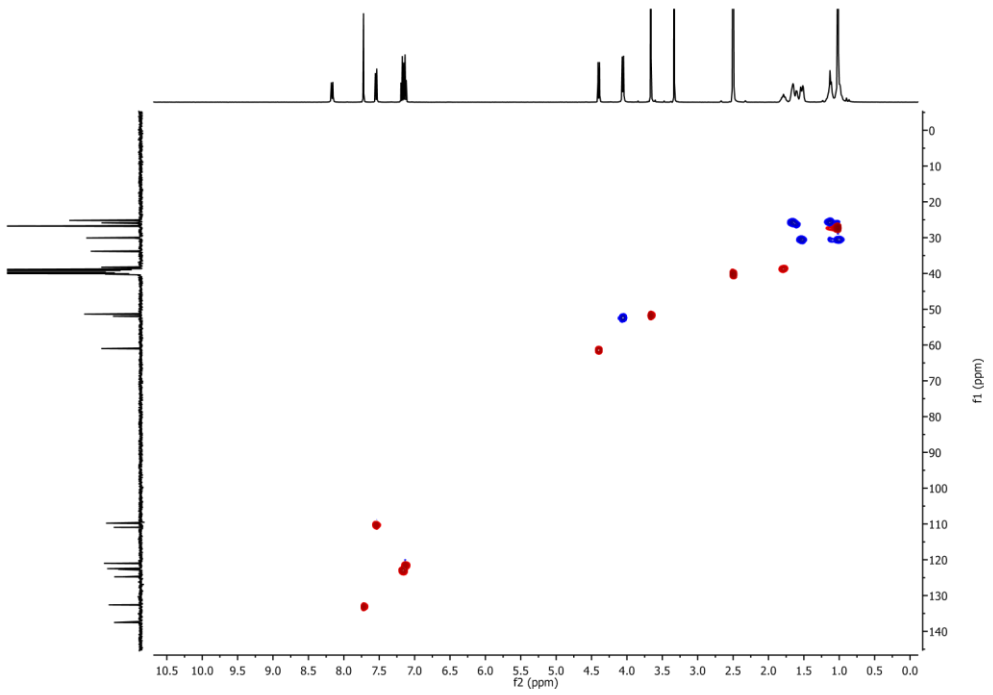

Figure S-69: HSQC of 3 (100.6 MHz, DMSO-d_6)

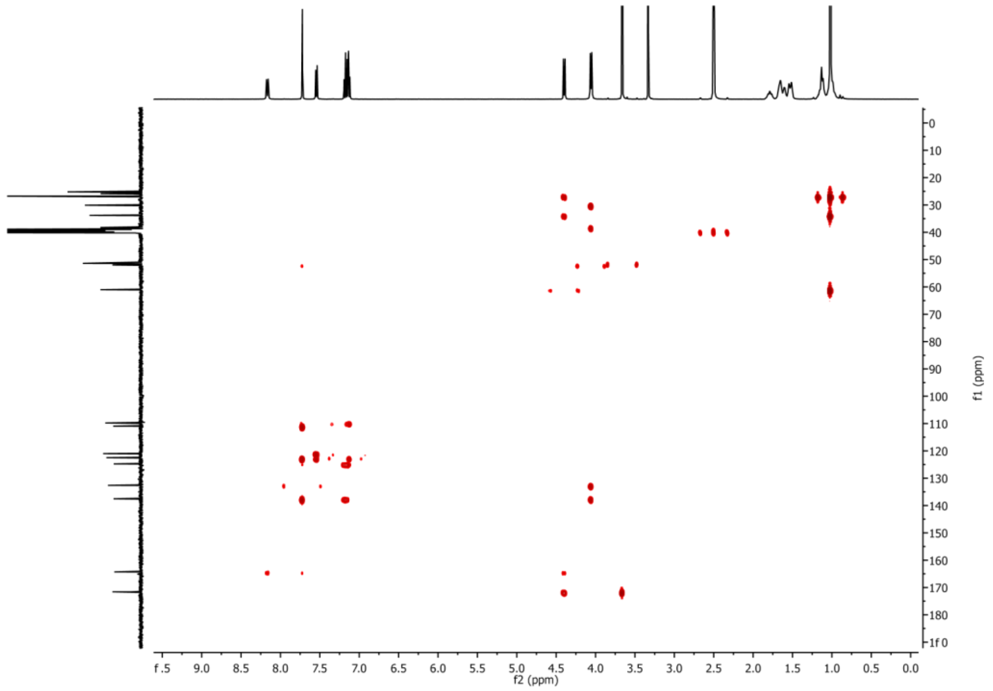

Figure S-70: HMBC of 3 (100.6 MHz, DMSO-d_6)

Methyl (*S*)-2-(5-chloro-1-(cyclohexylmethyl)-1*H*-indole-3-carboxamido)-3,3-dimethylbutanoate (5-Chloro-MDMB-CHMICA) (4)

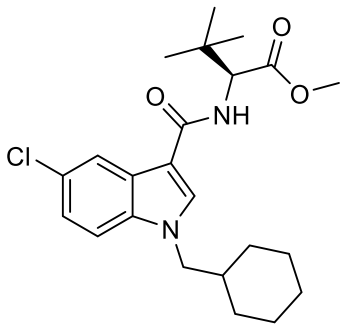

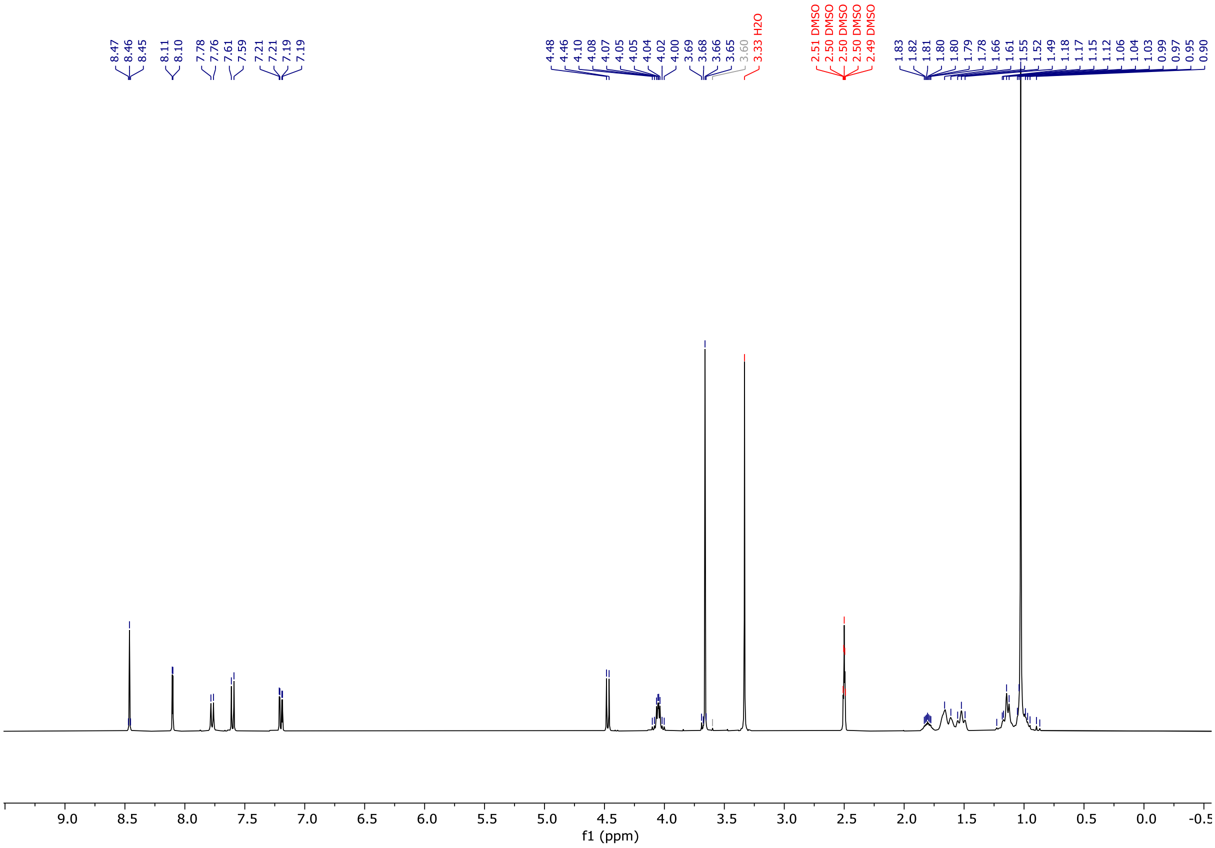

Figure S-71: ^{1}H-NMR of 4 (400 MHz, DMSO-d_6)

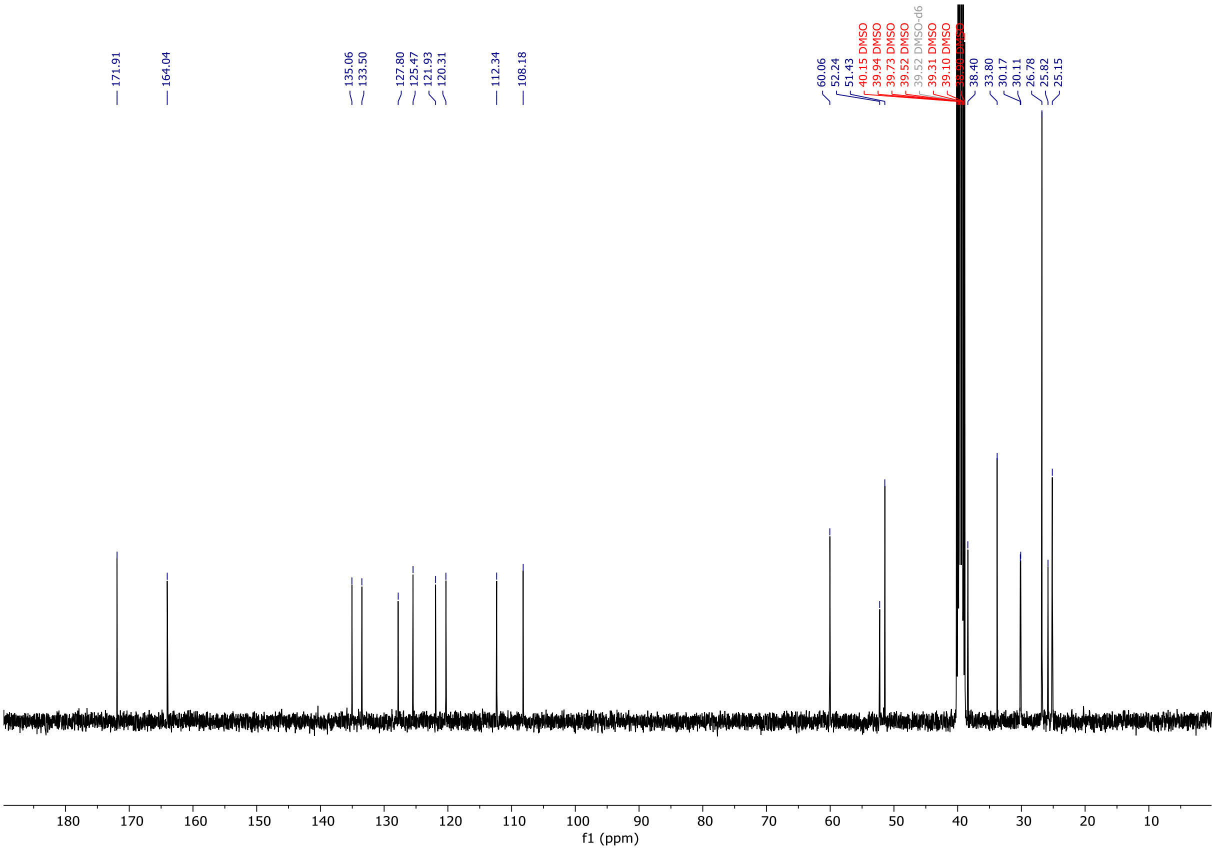

Figure S-72: ^{13}C-NMR of 4 (100.6 MHz, DMSO-*d6*)

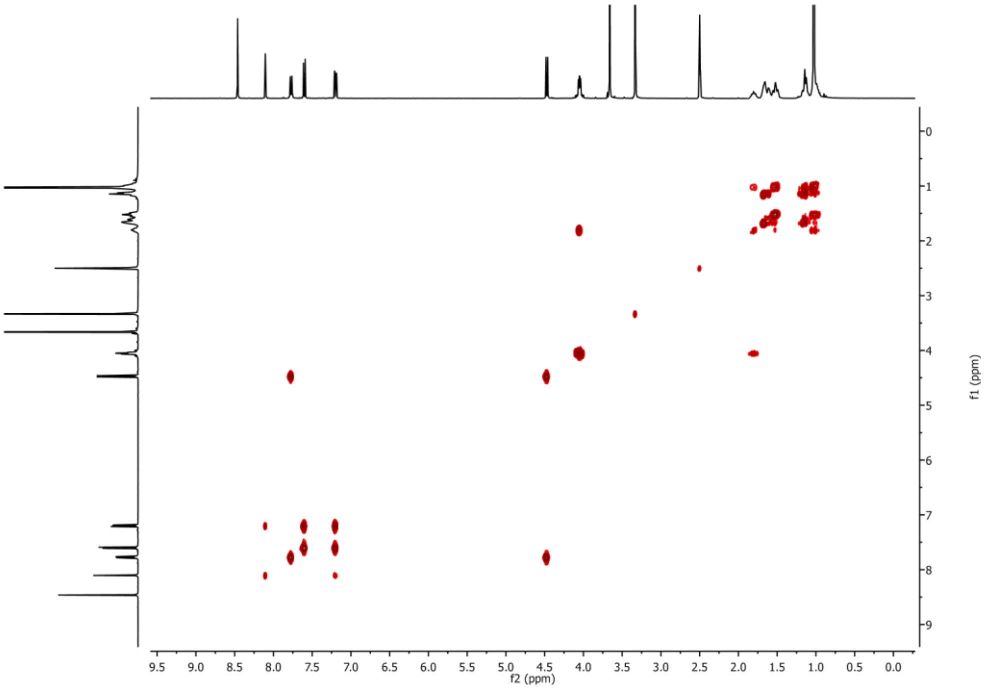

Figure S-73: COSY of 4 (400 MHz, DMSO-d_6)

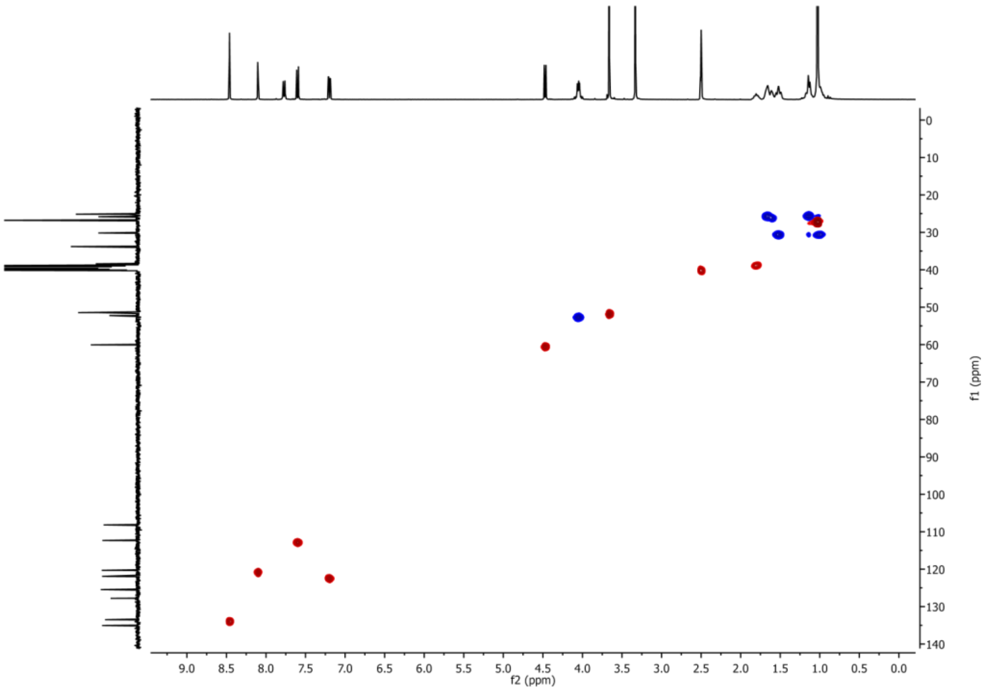

Figure S-74: HSQC of 4 (100.6 MHz, DMSO-*d*$_6$)

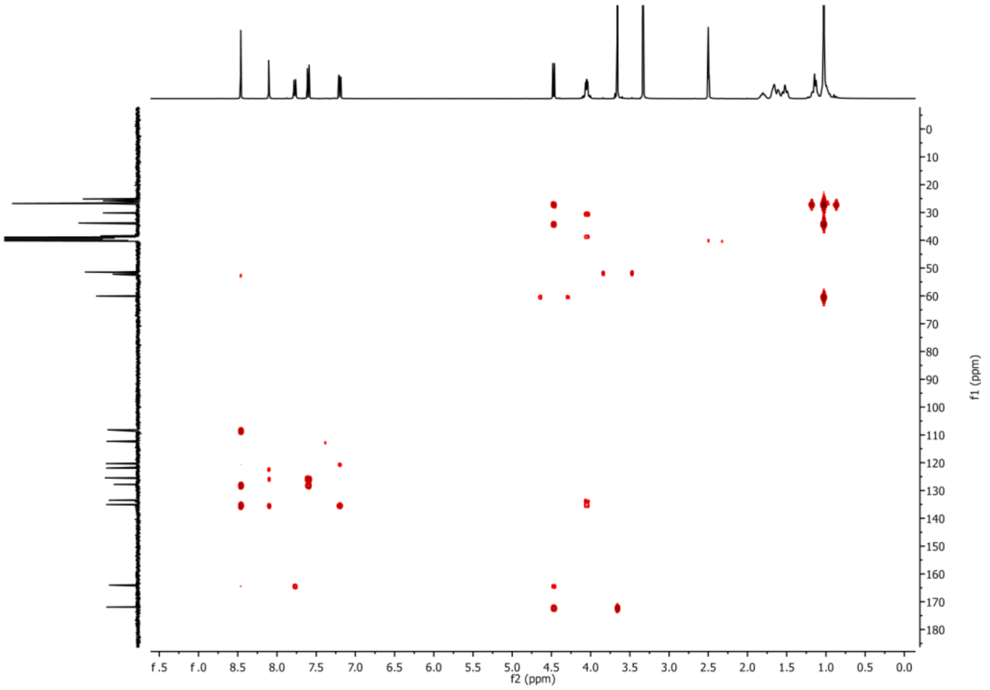

Figure S-75: HMBC of 4 (100.6 MHz, DMSO-*d*$_6$)

Methyl (*S*)-2-(6-chloro-1-(cyclohexylmethyl)-1*H*-indole-3-carboxamido)-3,3-dimethylbutanoate (6-Chloro-MDMB-CHMICA) (5)

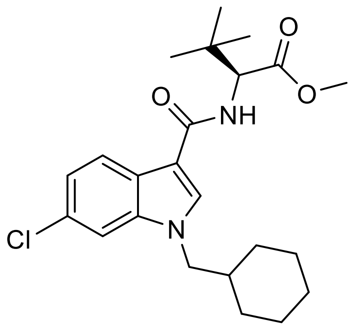

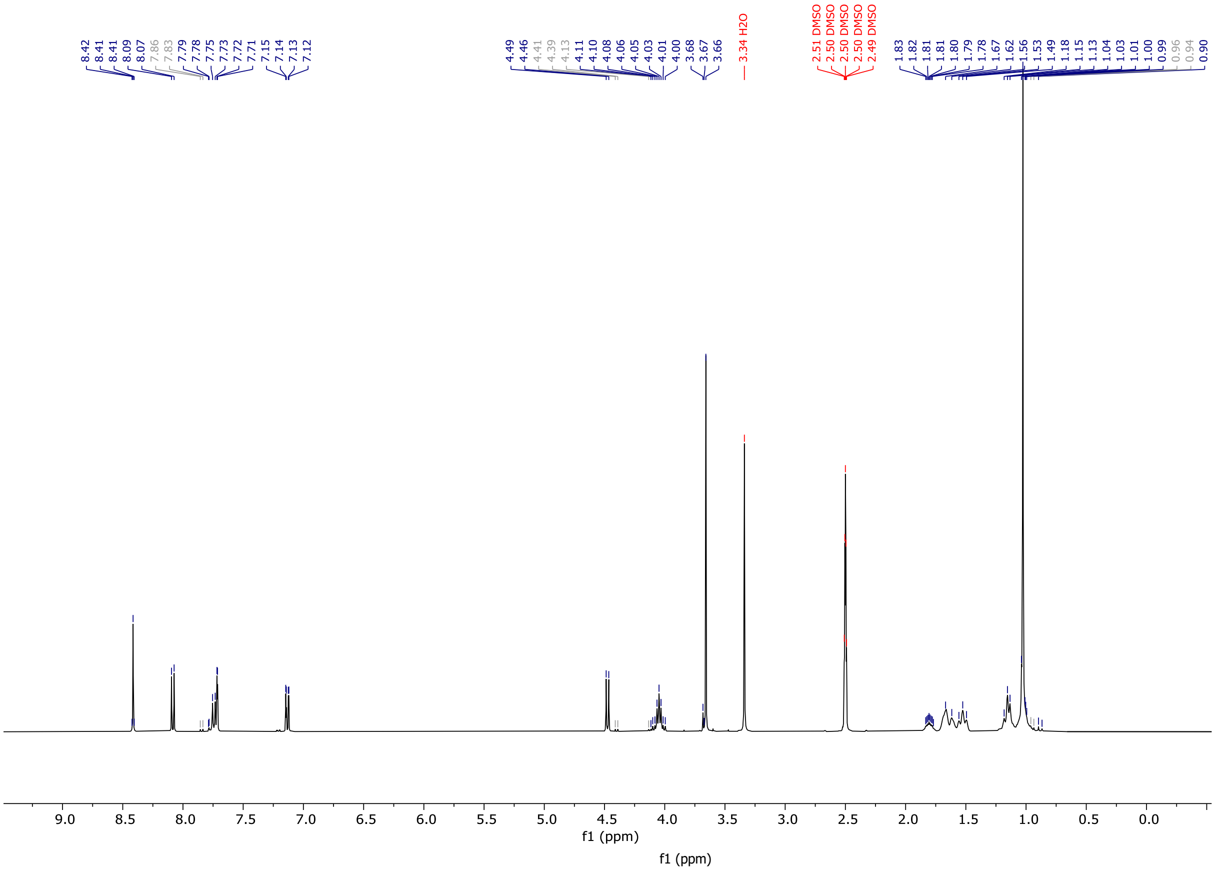

Figure S-76: ^{1}H-NMR of 5 (400 MHz, DMSO-d_6)

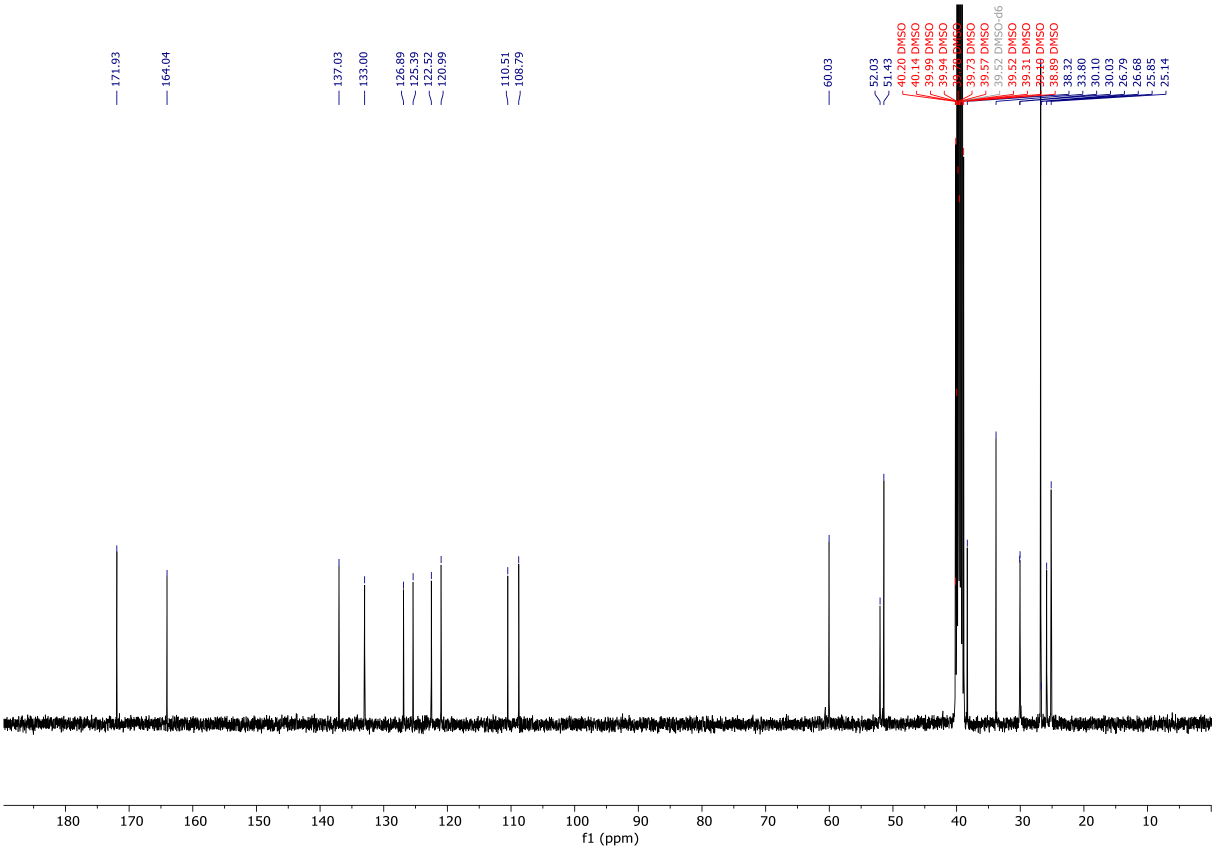

Figure S-77: ^{13}C-NMR of 5 (100.6 MHz, DMSO-*d6*)

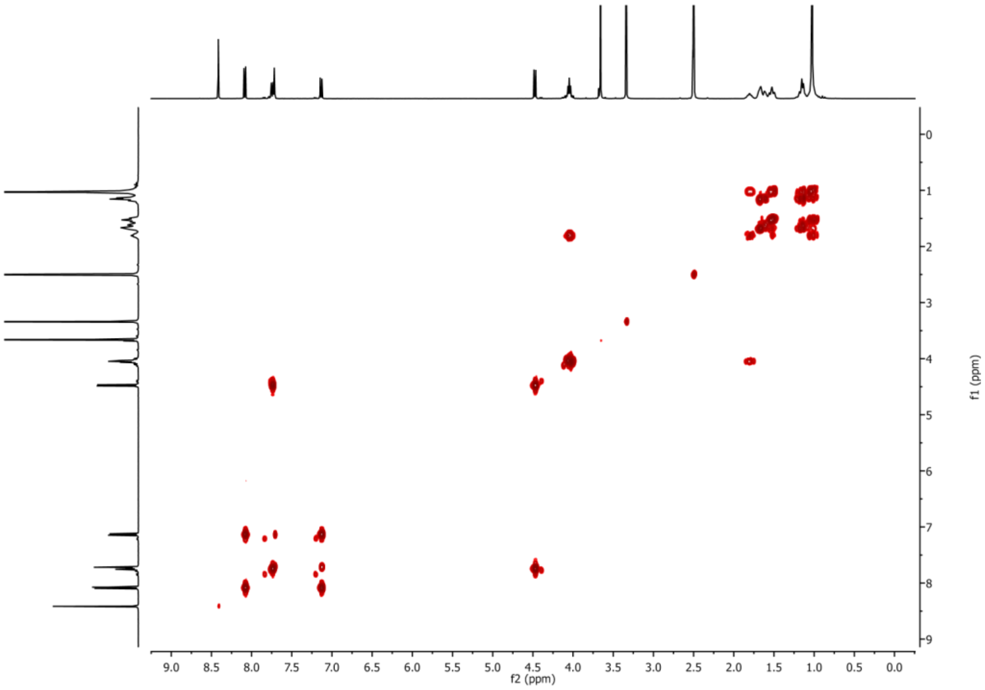

Figure S-78: COSY of 5 (400 MHz, DMSO-d_6)

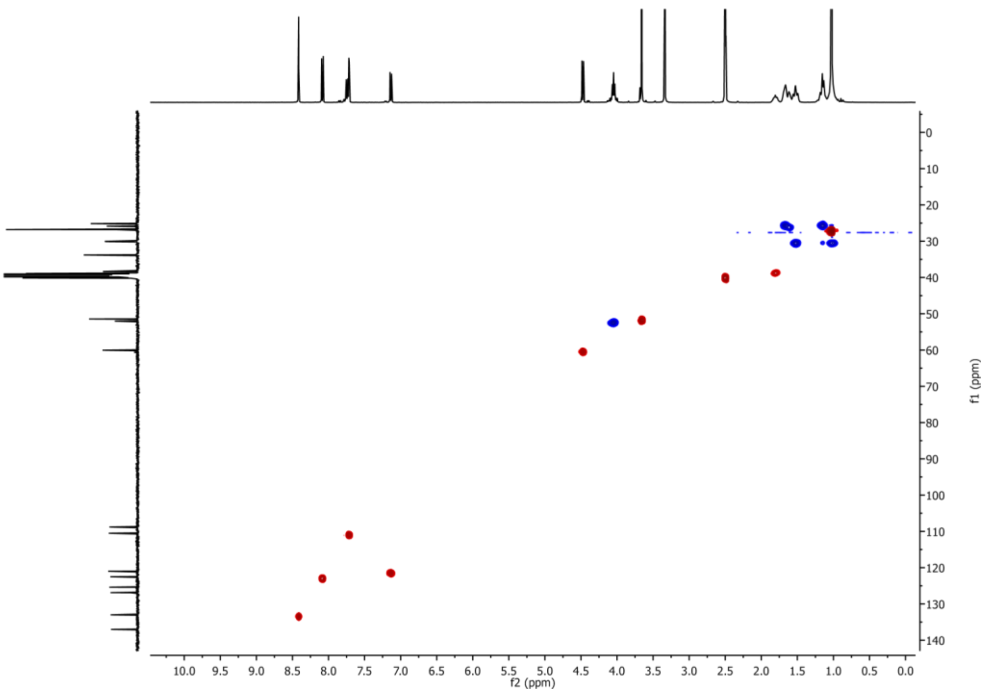

Figure S-79: HSQC of 5 (100.6 MHz, DMSO-d_6)

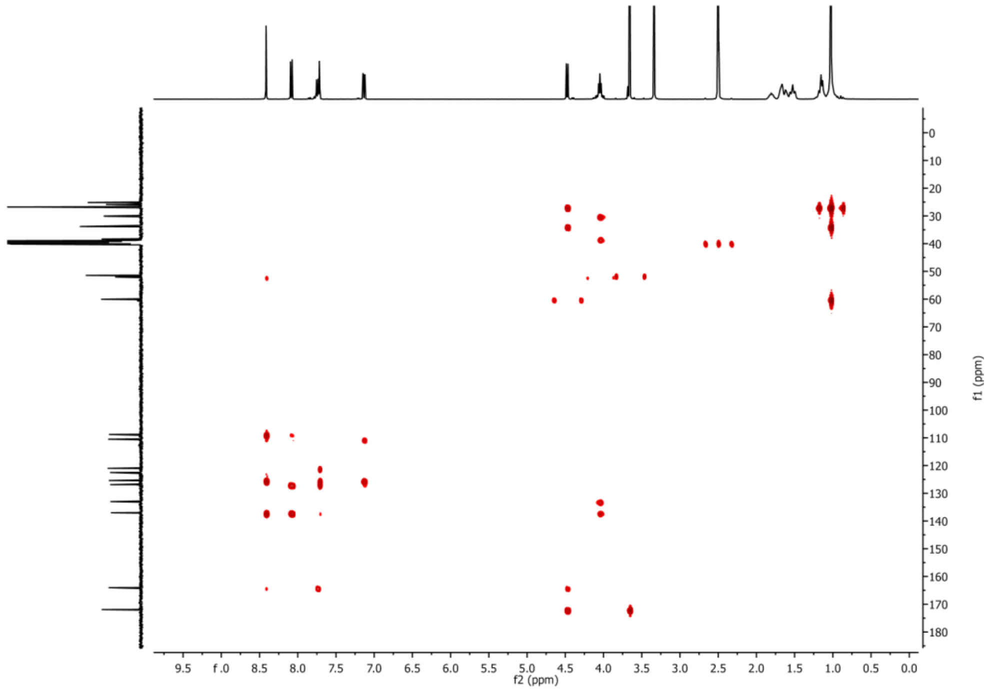

Figure S-80: HMBC of 5 (100.6 MHz, DMSO-d_6)

Methyl (*S*)-2-(7-chloro-1-(cyclohexylmethyl)-1*H*-indole-3-carboxamido)-3,3-dimethylbutanoate (7-Chloro-MDMB-CHMICA) (6)

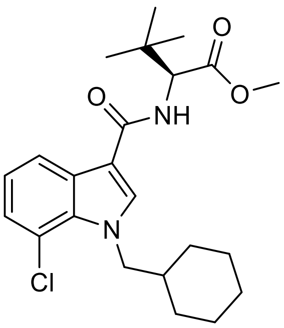

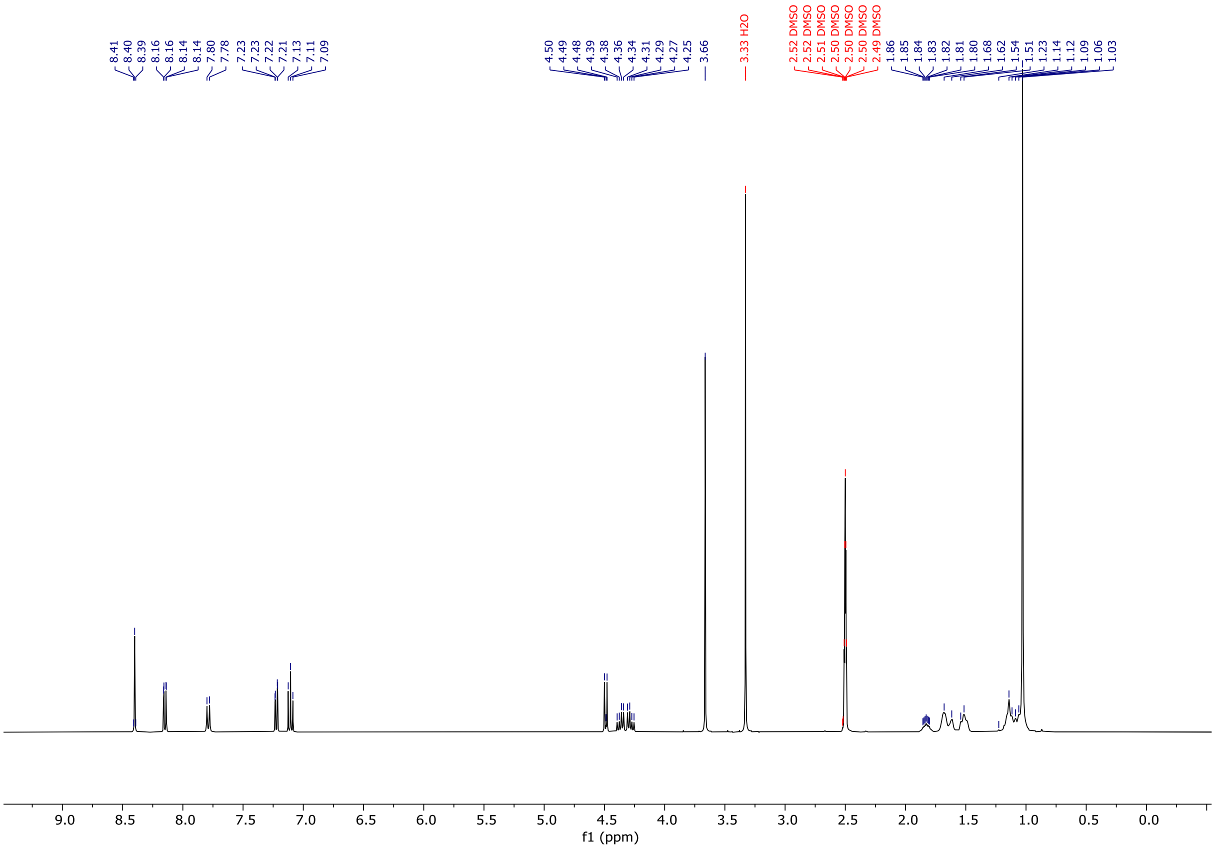

Figure S-81: ^{1}H-NMR of 6 (400 MHz, DMSO-d_6)

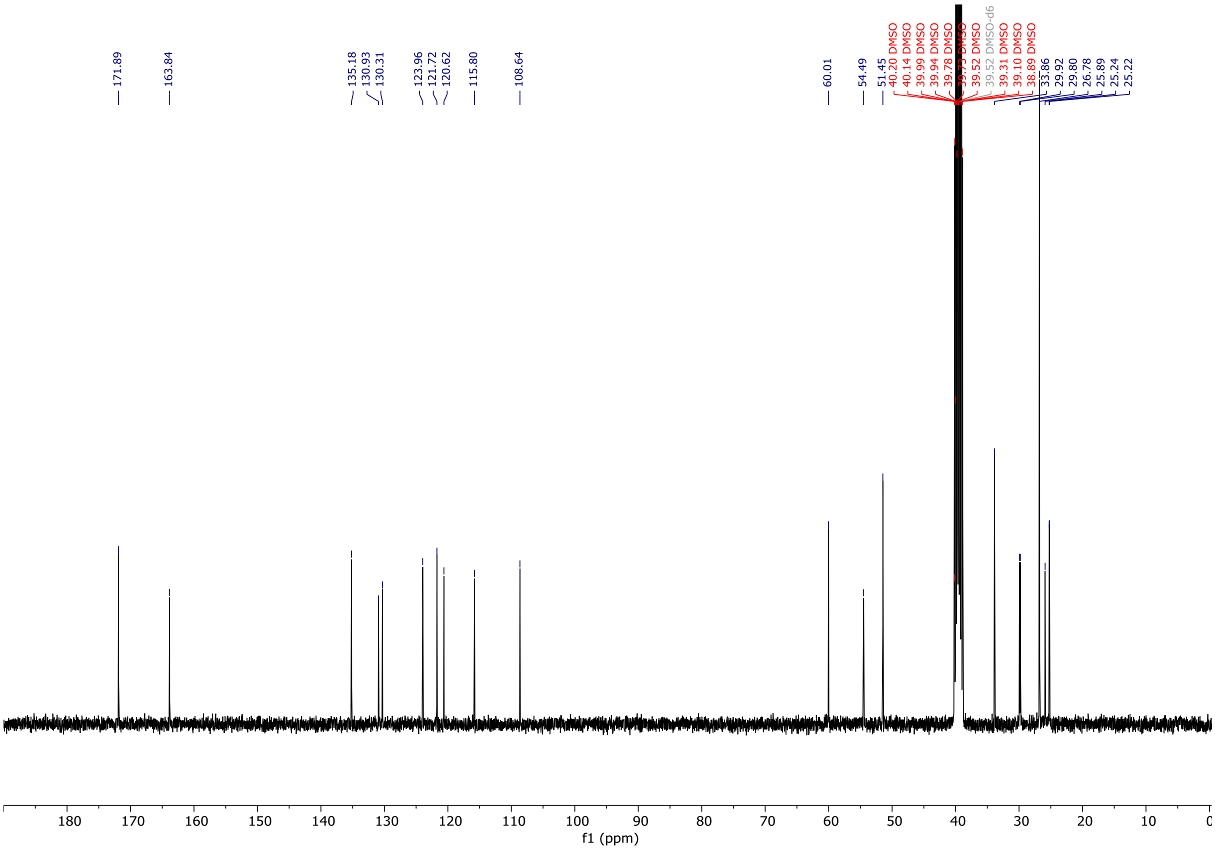

Figure S-82: ^{13}C-NMR of 6 (100.6 MHz, DMSO-*d6*)

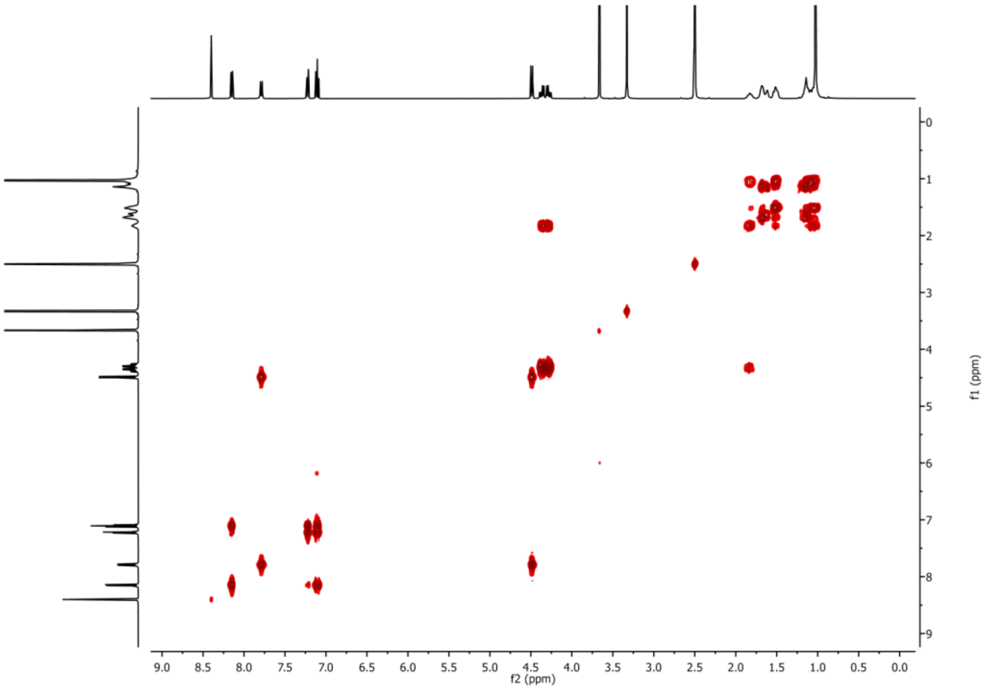

Figure S-83: COSY of 6 (400 MHz, DMSO-d_6)

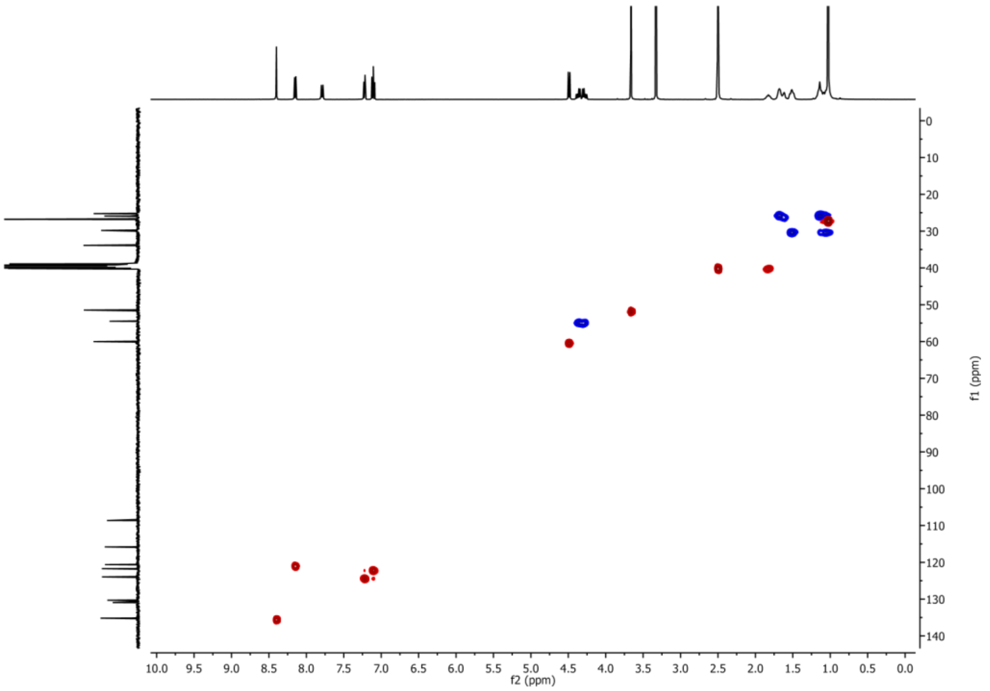

Figure S-84: HSQC of 6 (100.6 MHz, DMSO-d_6)

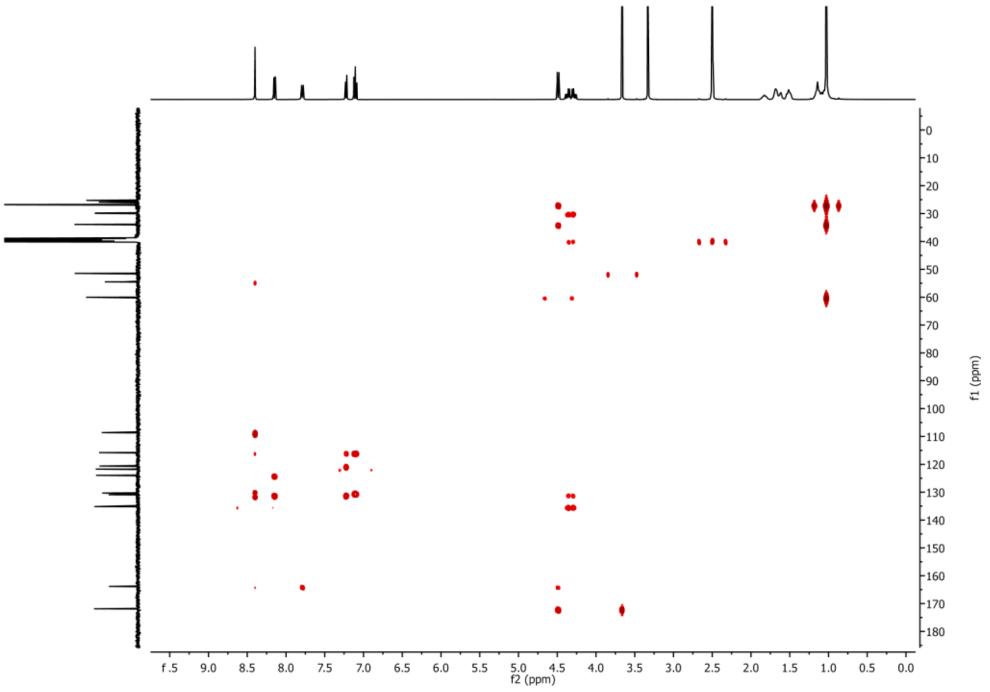

Figure S-85: HMBC of 6 (100.6 MHz, DMSO-d_6)

GC-MS spectra

Figure S-86 to S-90 show the EI-GC-MS spectra of 2-Cl-, 4-Cl-, 5-Cl-, 6-Cl- and 7-Cl-MDMB-CHMICA.

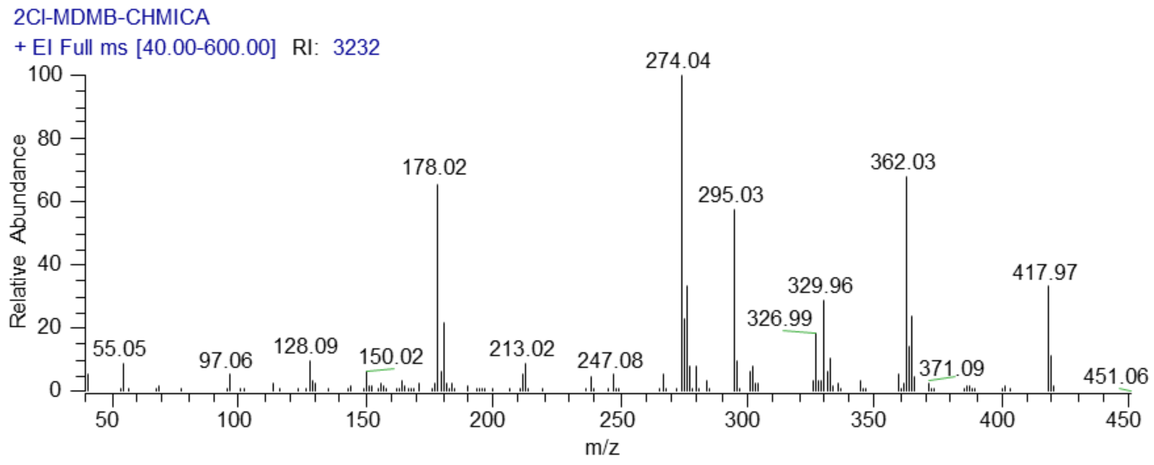

Figure S-86: EI-GC-MS spectrum of 2-Cl-MDMB-CHMICA

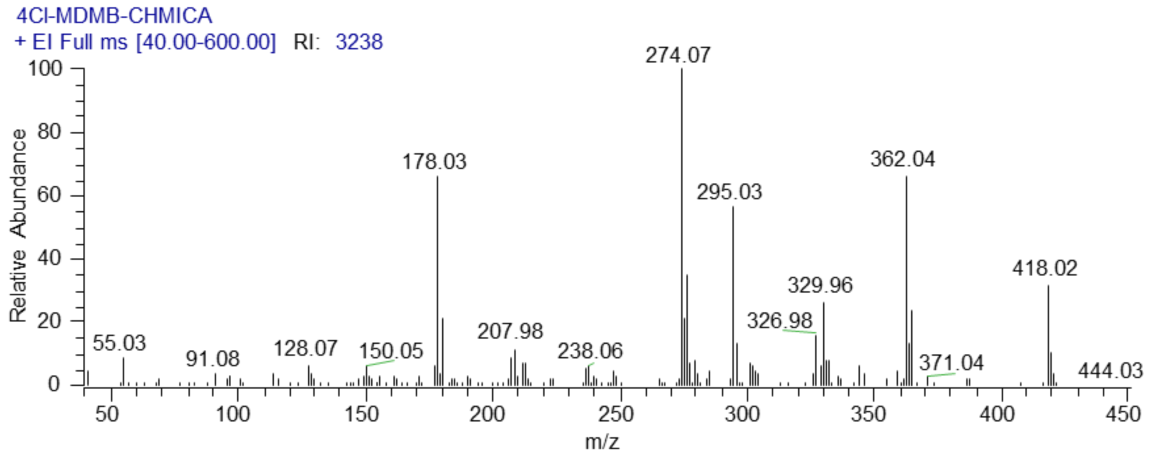

Figure S-87: EI-GC-MS spectrum of 4-Cl-MDMB-CHMICA

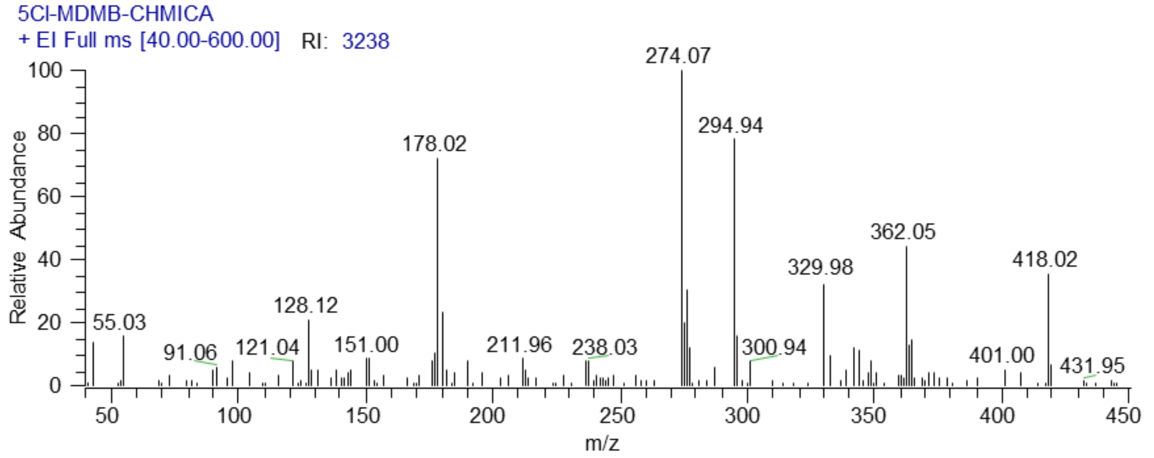

Figure S-88: EI-GC-MS spectrum of 5-Cl-MDMB-CHMICA

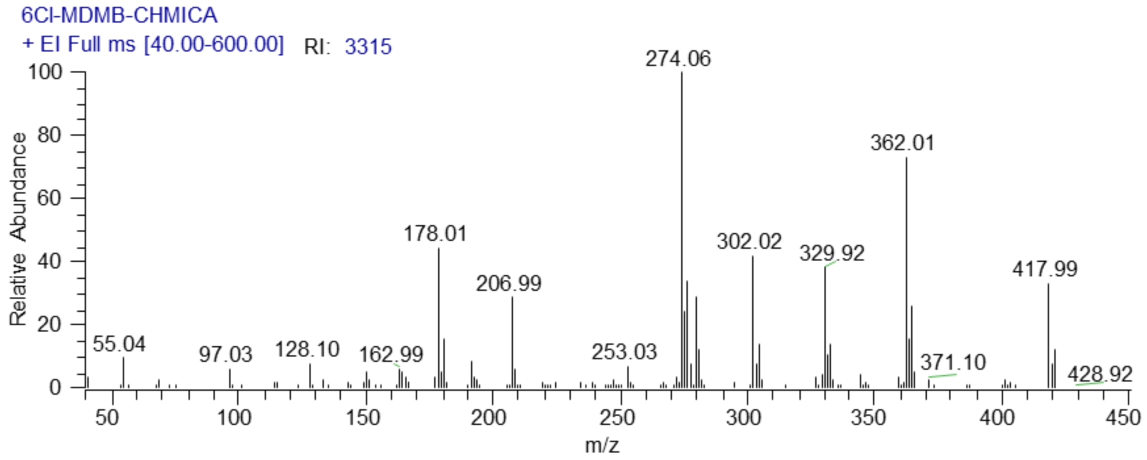

Figure S-89: EI-GC-MS spectrum of 6-Cl-MDMB-CHMICA

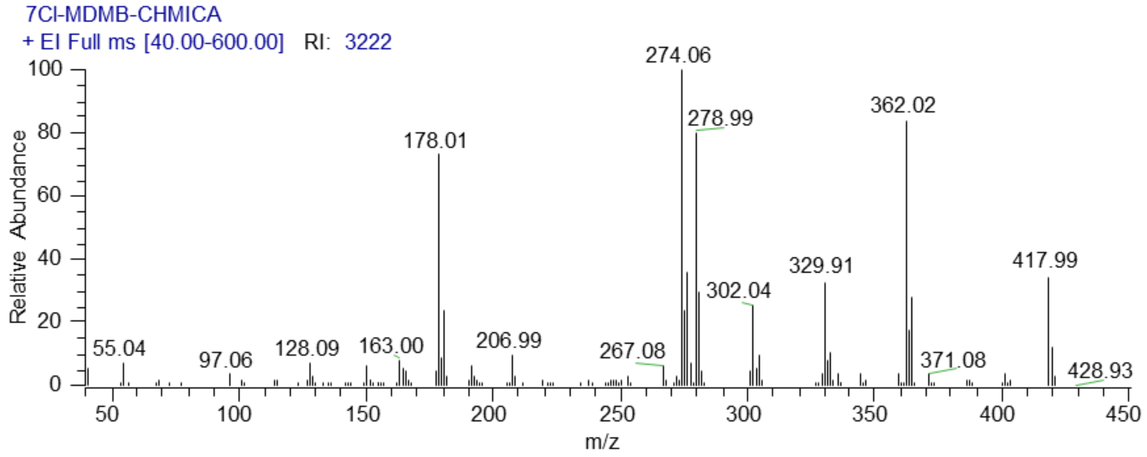

Figure S-90: EI-GC-MS spectrum of 7-Cl-MDMB-CHMICA

IR Spectra

Figure S-91 to S-97 show the GC-sIR spectra (overview and fingerprint area) of 2-Cl-, 4-Cl-, 5-Cl-, 6-Cl- and 7Cl-MDMB-CHMICA.

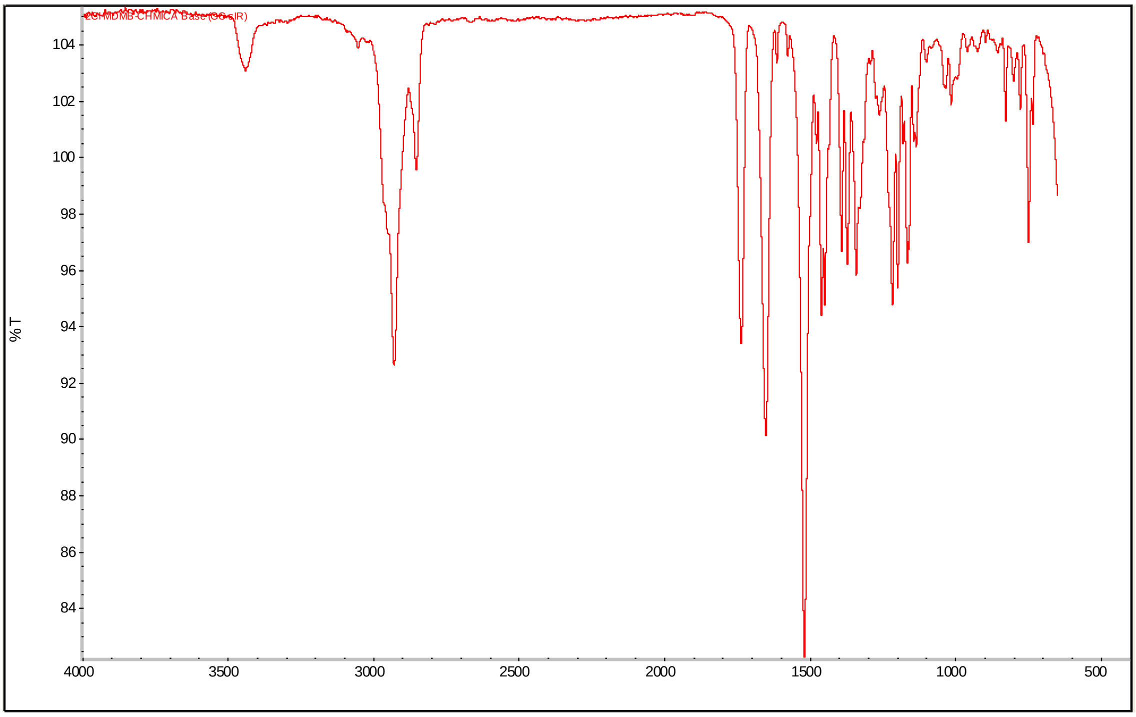

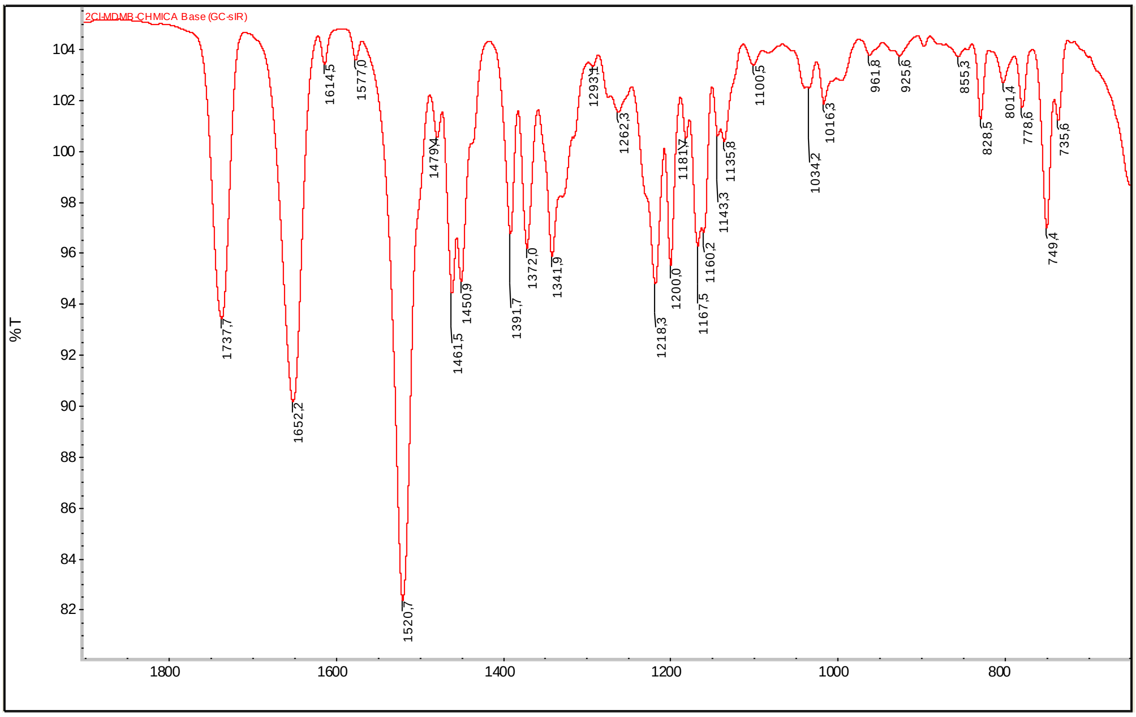

Figure S-91: (Top) GC-sIR spectrum of 2-Cl-MDMB-CHMICA. (Bottom) Zoom into the corresponding fingerprint area

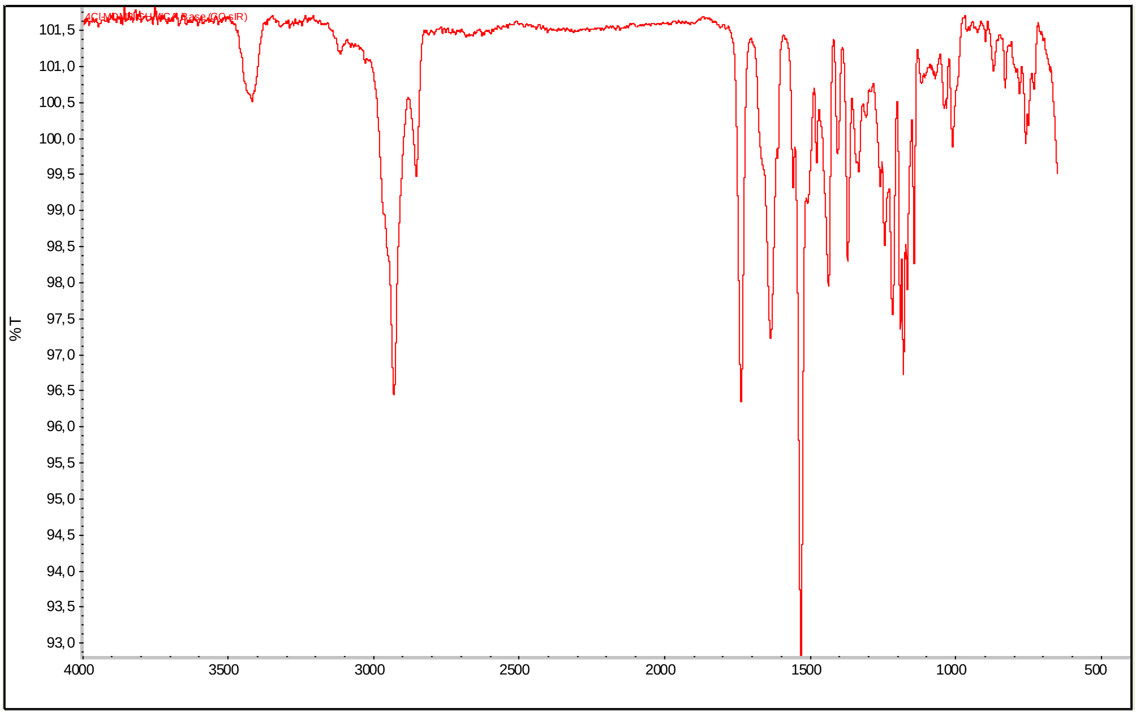

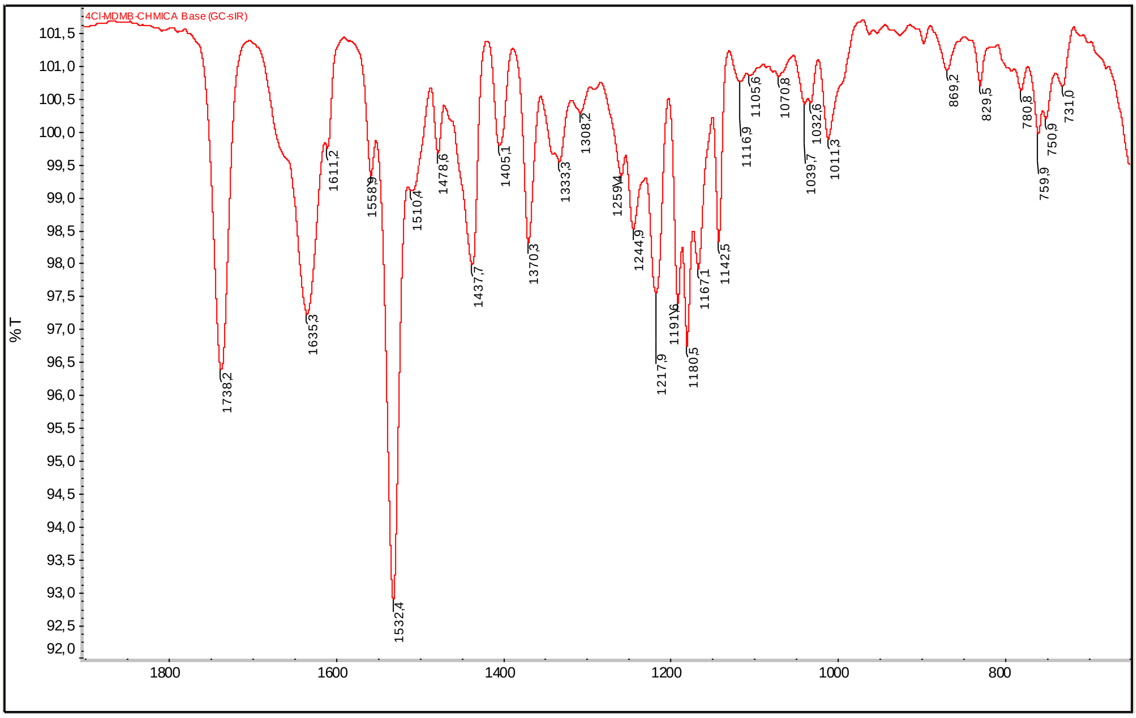

Figure S-92: (Top) GC-sIR spectrum of 4-Cl-MDMB-CHMICA. (Bottom) Zoom into the corresponding fingerprint area

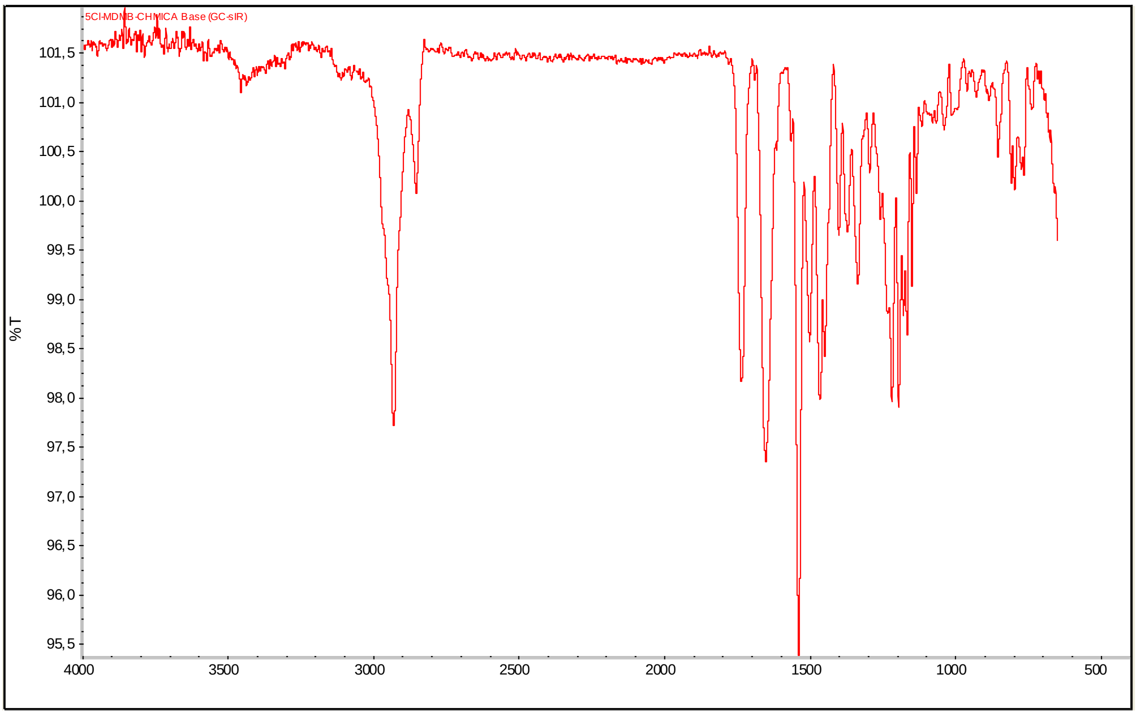

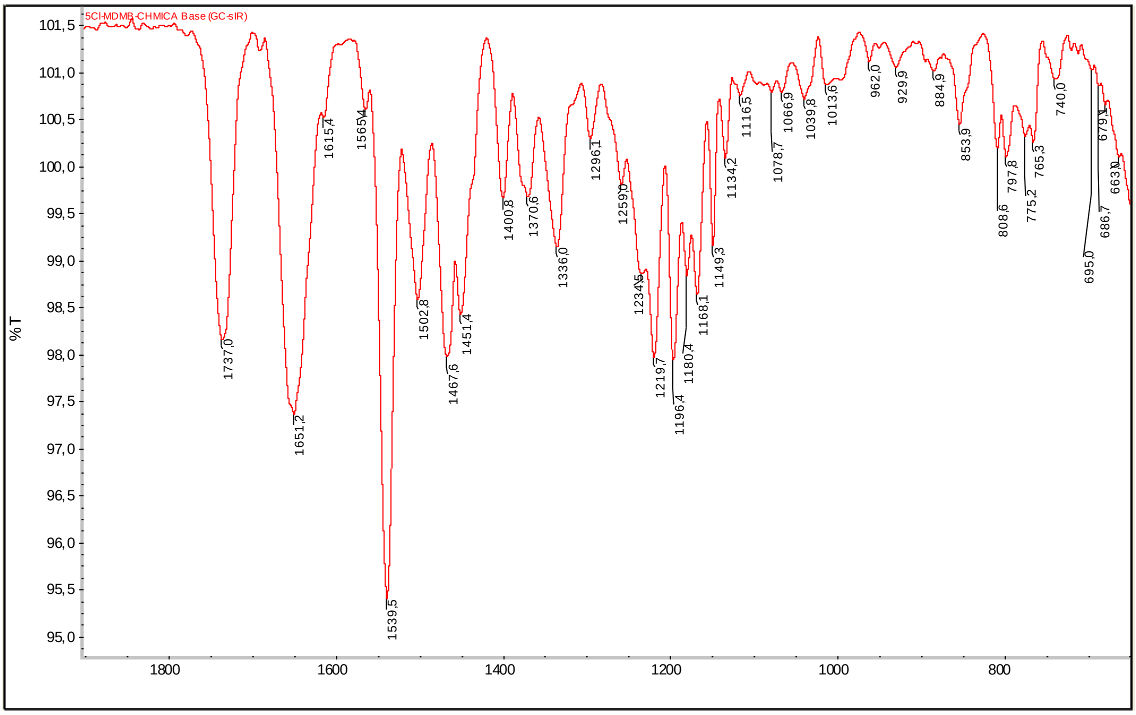

Figure S-93: (Top) GC-sIR spectrum of 5-Cl-MDMB-CHMICA. (Bottom) Zoom into the corresponding fingerprint area

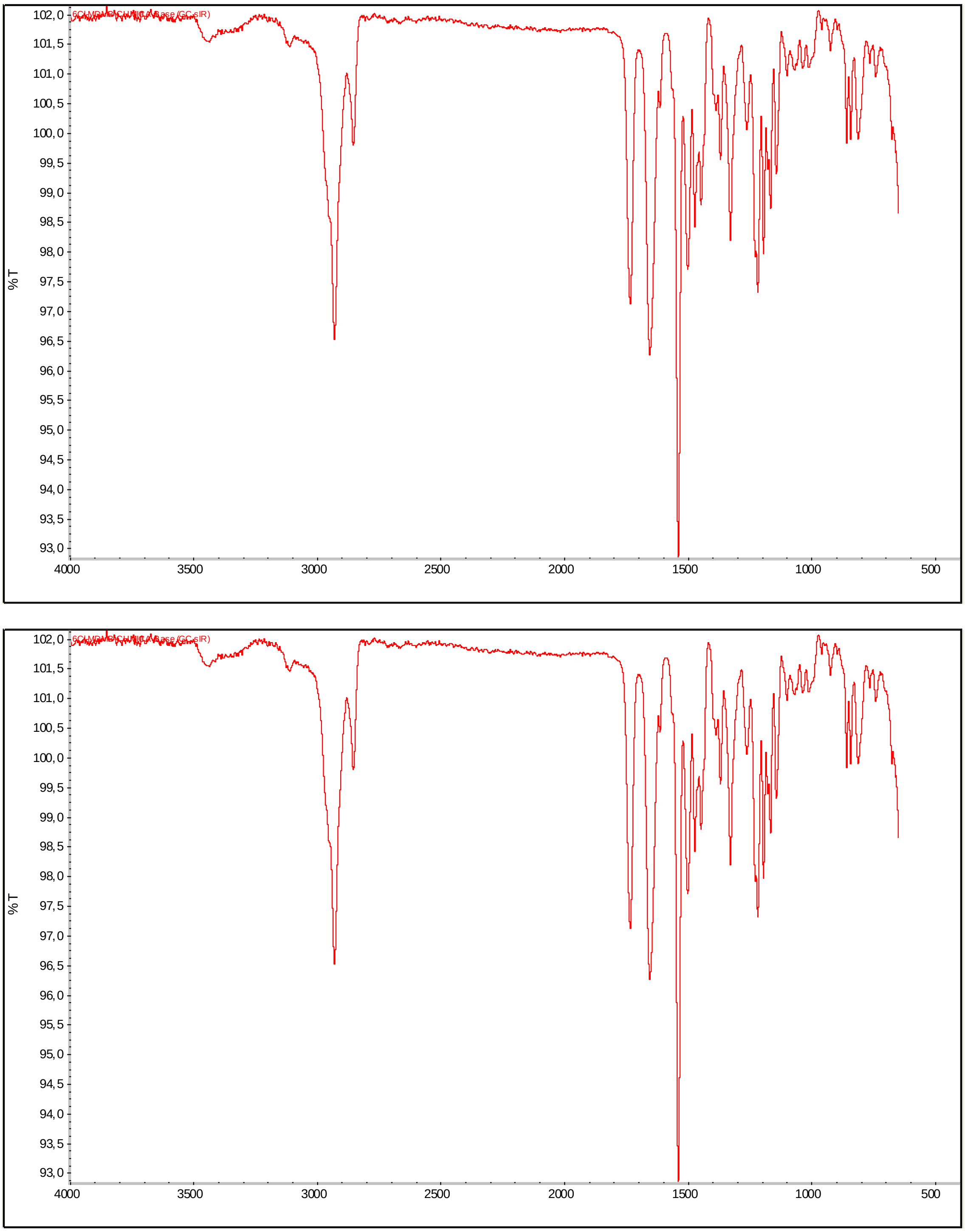

Figure S-94: (Top) GC-sIR spectrum of 6-Cl-MDMB-CHMICA. (Bottom) Zoom into the corresponding fingerprint area

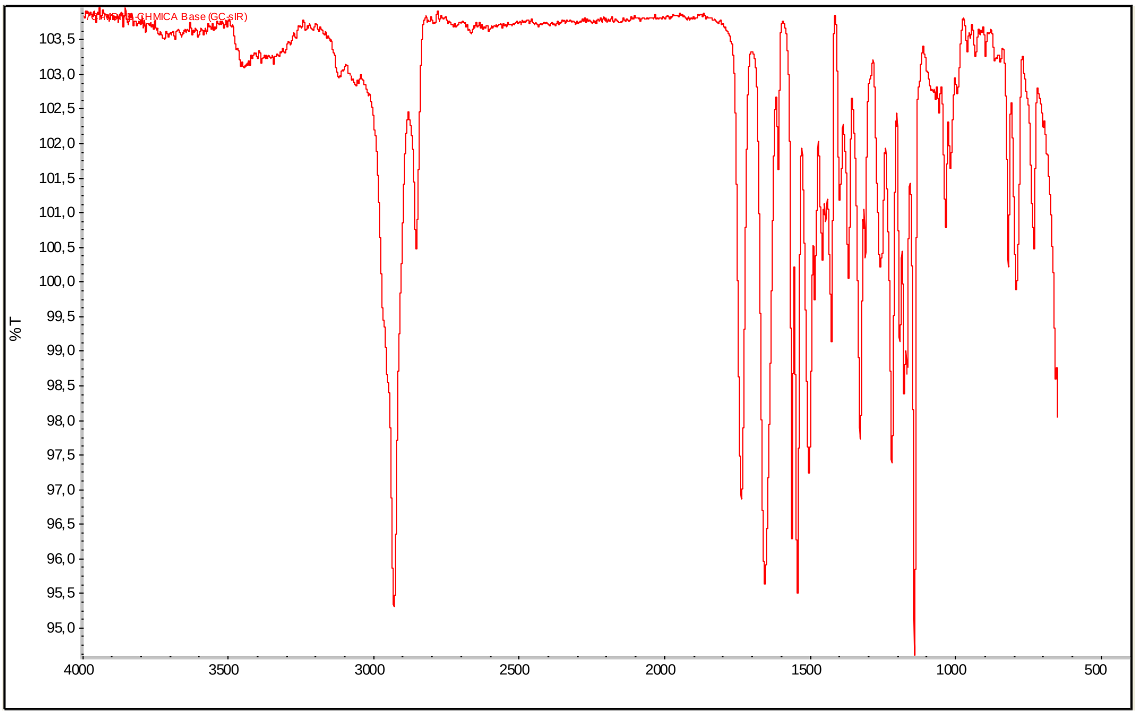

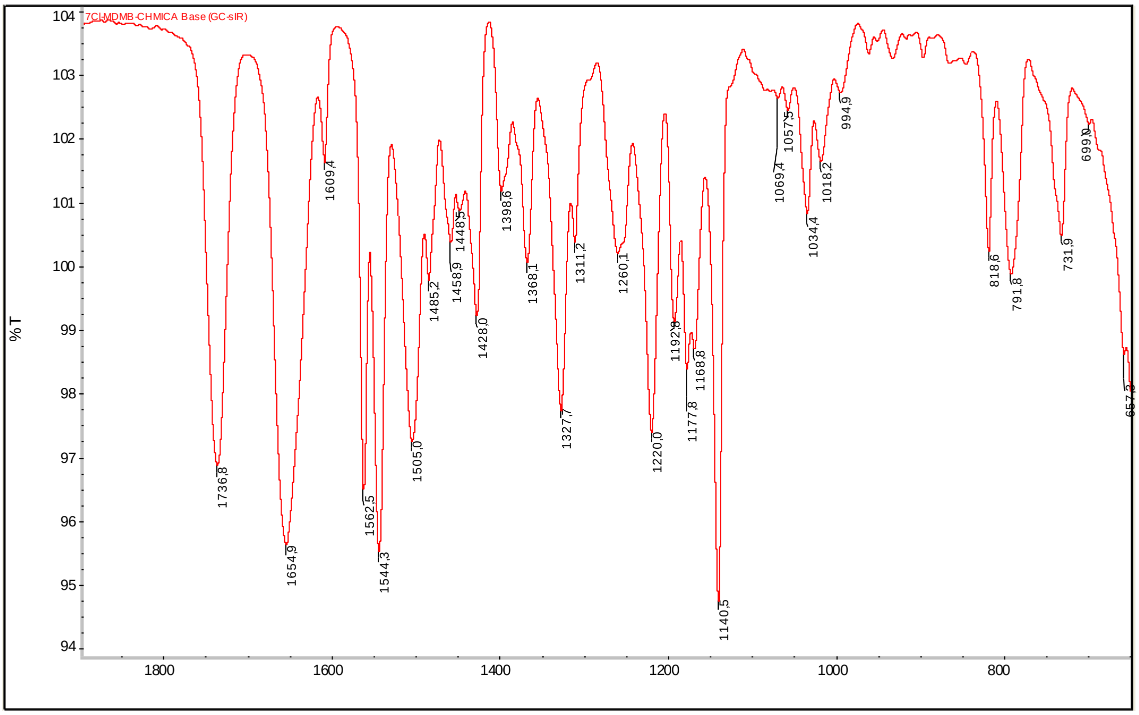

Figure S-95: (Top) GC-sIR spectrum of 7-Cl-MDMB-CHMICA. (Bottom) Zoom into the corresponding fingerprint area

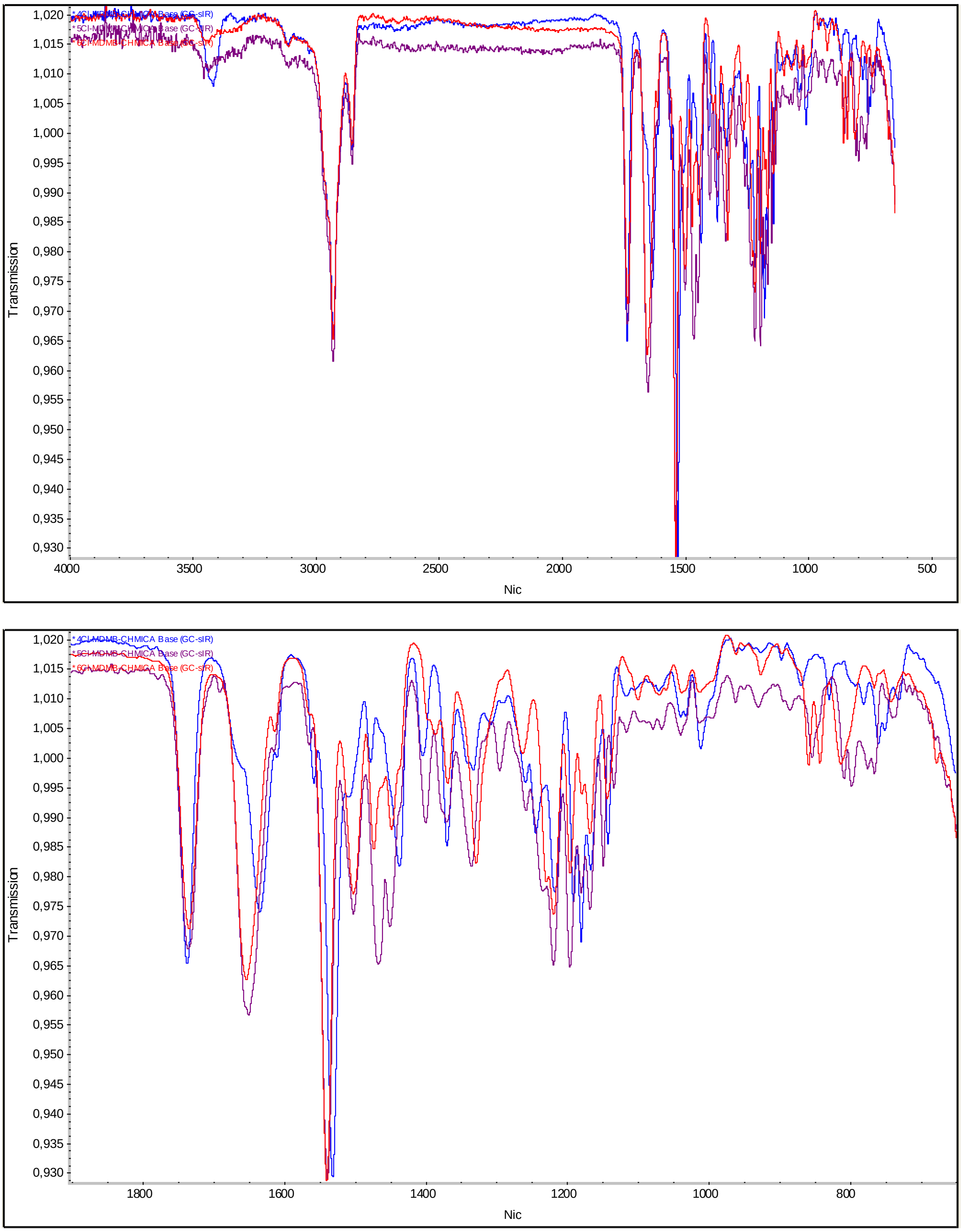

Figure S-96: (Top) stacked GC-sIR spectrum of 4-Cl- (blue), 5-Cl (purple) and 6-Cl-MDMB-CHMICA (red). (Bottom) Zoom into the corresponding fingerprint area

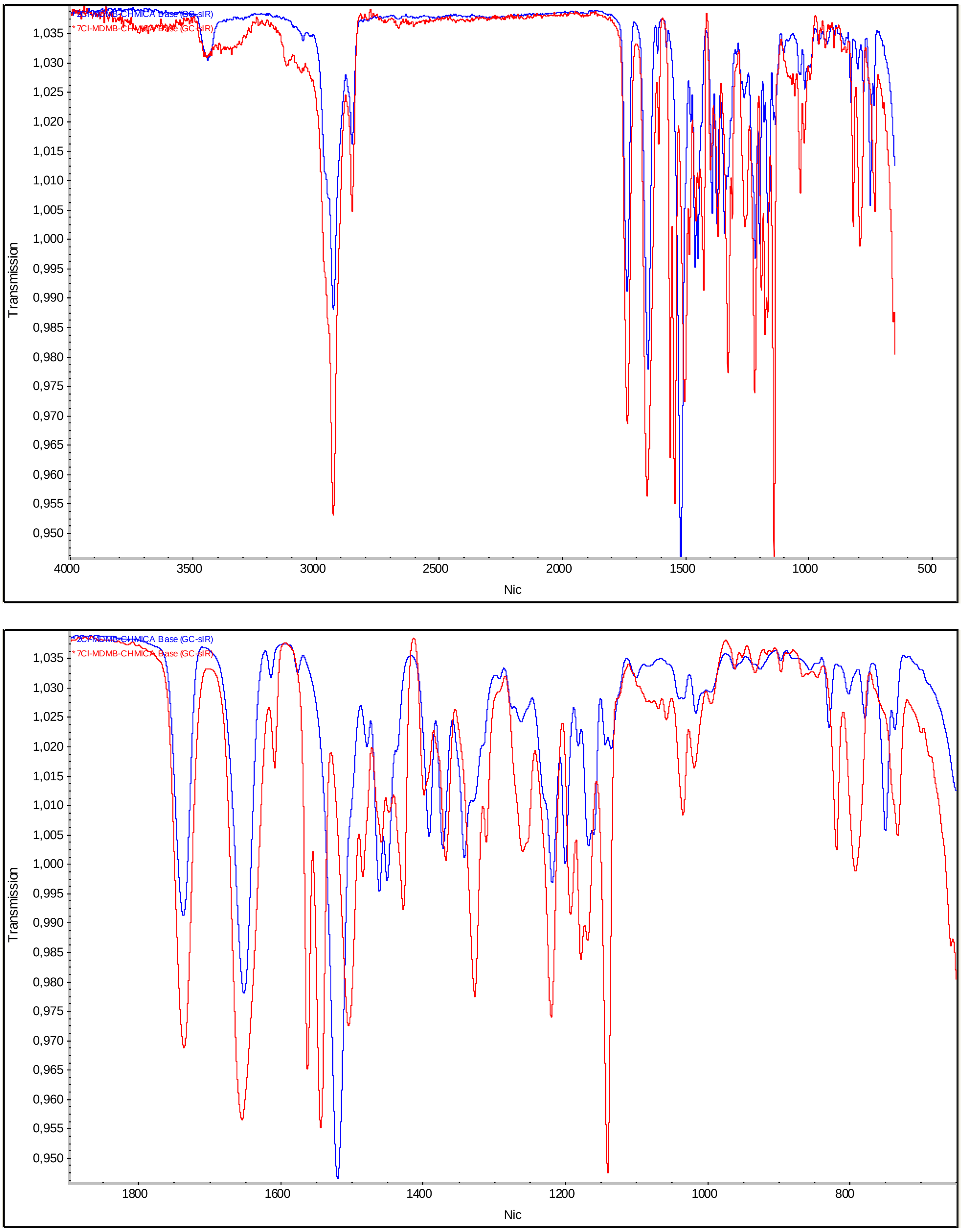

Figure S-97: (Top) stacked GC-sIR spectrum of 2-Cl (blue) and 7-Cl-MDMB-CHMICA (red). (Bottom) Zoom into the corresponding fingerprint area

UV Spectra

Figure S-98 to S-102 show the UV/VIS spectra of 2-Cl-, 4-Cl-, 5-Cl-, 6-Cl- and 7-Cl-MDMB-CHMICA.

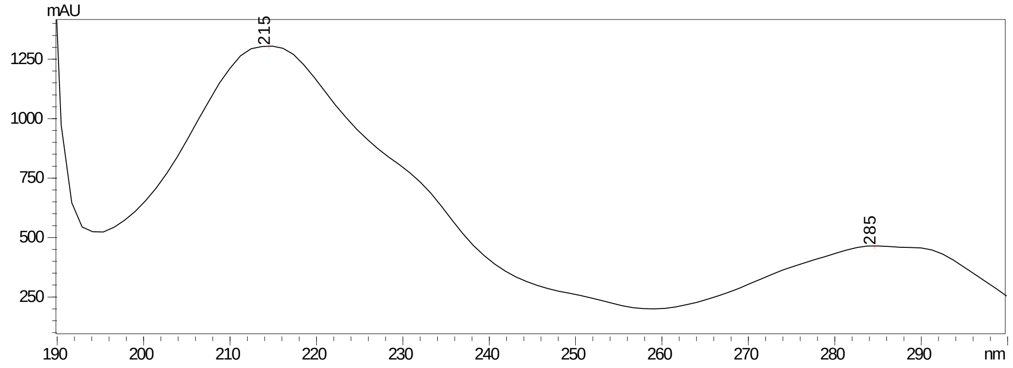

Figure S-98: UV/VIS spectrum of 2-Cl-MDMB-CHMICA

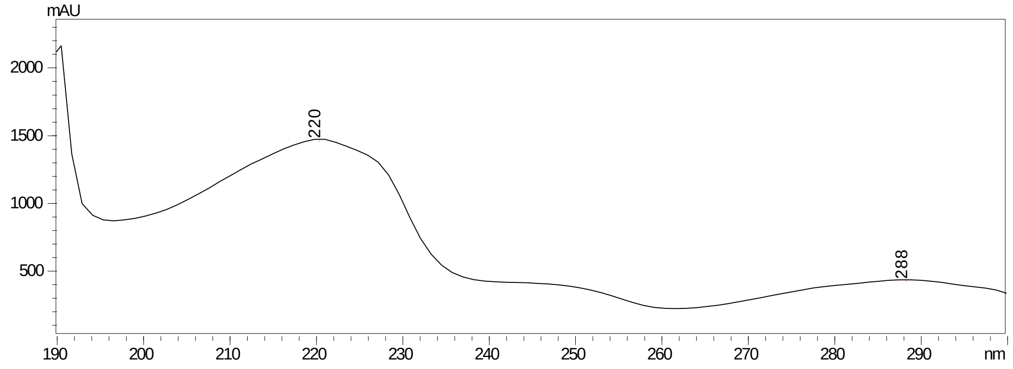

Figure S-99: UV/VIS spectrum of 4-Cl-MDMB-CHMICA

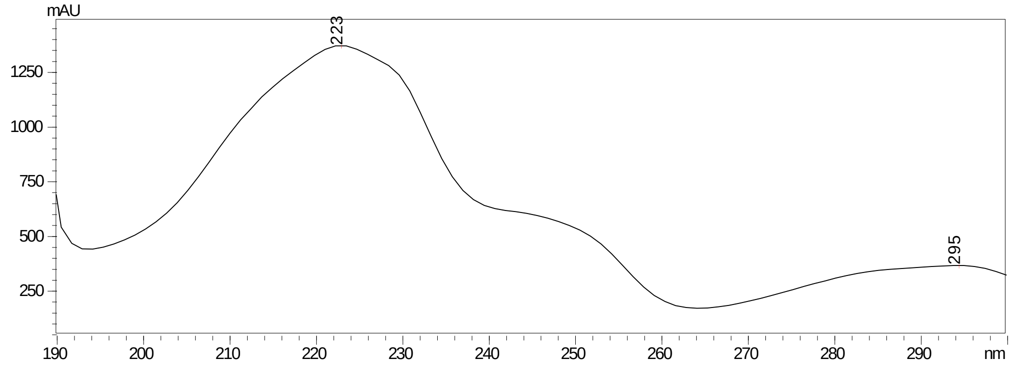

Figure S-100: UV/VIS spectrum of 5-Cl-MDMB-CHMICA

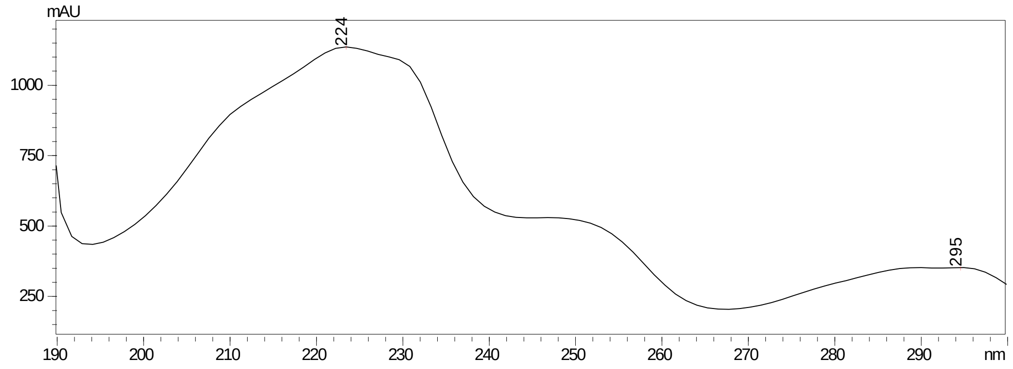

Figure S-101: UV/VIS spectrum of 6-Cl-MDMB-CHMICA

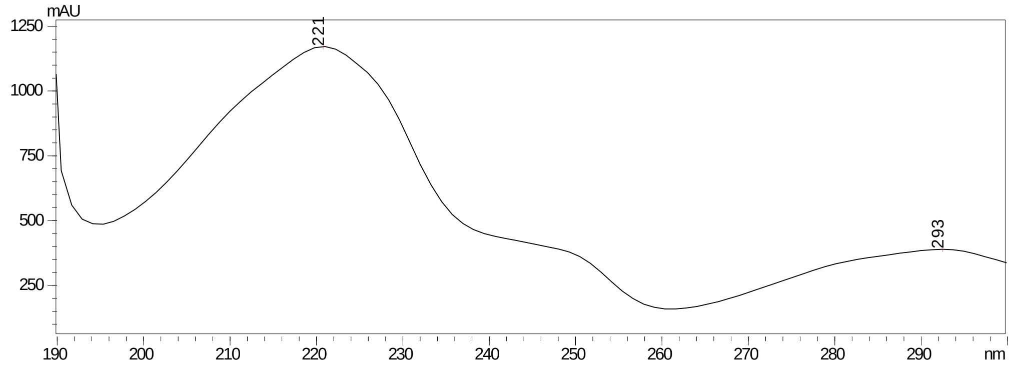

Figure S-102: UV/VIS spectrum of 7-Cl-MDMB-CHMICA

CB_1 receptor binding assay

Figure S-103 to S-107 show CB_1 receptor binding assay of 2-Cl-, 4-Cl-, 5-Cl-, 6-Cl- and 7-Cl-MDMB-CHMICA.

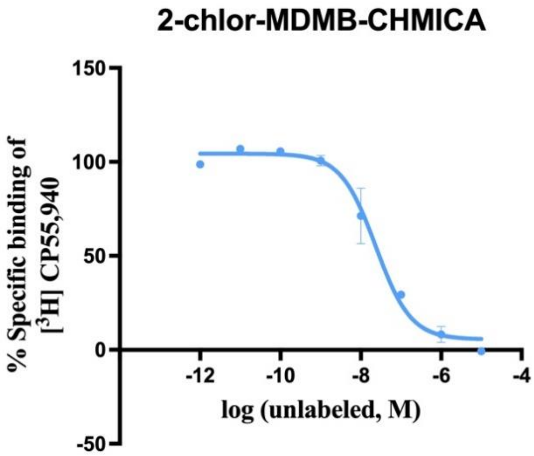

Figure S-103: CB_1 receptor binding assay of 2-Cl-MDMB-CHMICA: calculated K_i (nM) 0.58, Fit K_i (R_2) 0.973

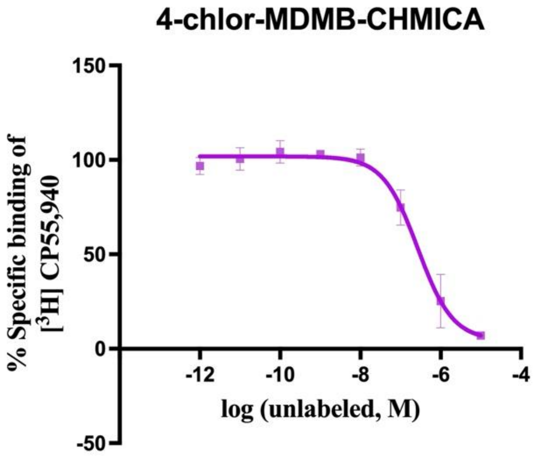

Figure S-104: CB_1 receptor binding assay of 4-Cl-MDMB-CHMICA: calculated K_i (nM) 6.55, Fit K_i (R_2) 0.970

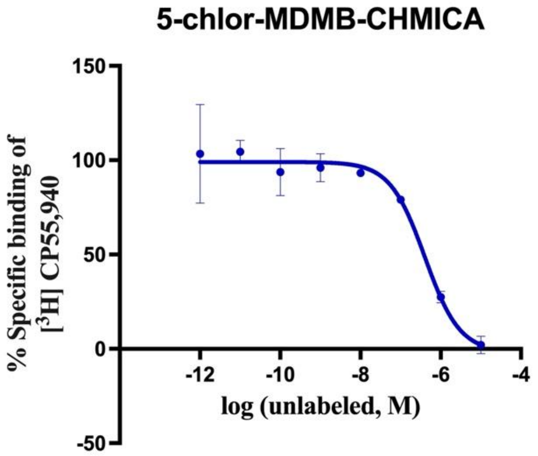

Figure S-105: CB_1 receptor binding assay of 5-Cl-MDMB-CHMICA: calculated K_i (nM) 9.81, Fit K_i (R_2) 0.947

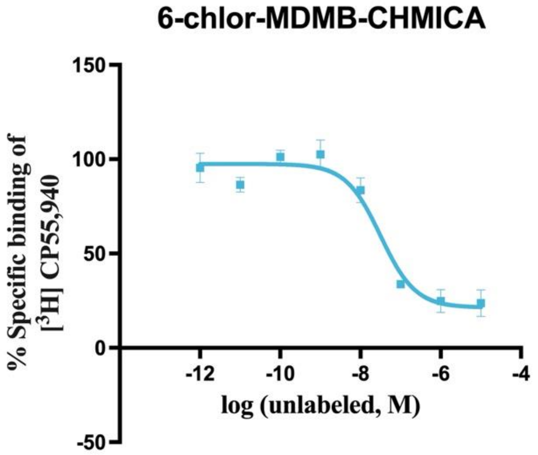

Figure S-106: CB_1 receptor binding assay of 6-Cl-MDMB-CHMICA: calculated K_i (nM) 0.77, Fit K_i (R_2) 0.955

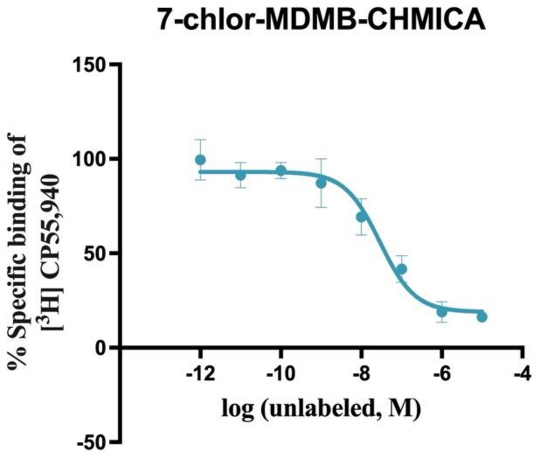

Figure S-107: CB_1 receptor binding assay of 7-Cl-MDMB-CHMICA: calculated K_i (nM) 0.70, Fit K_i (R_2) 0.947
